# Supplementary figures and images for: Antibacterial T6SS effectors with a VRR-Nuc domain are structure-specific nucleases
Source: eLife. 2022 Oct 13;11:e82437. doi: 10.7554/eLife.82437 (PMC9635880; doi:10.7554/eLife.82437)

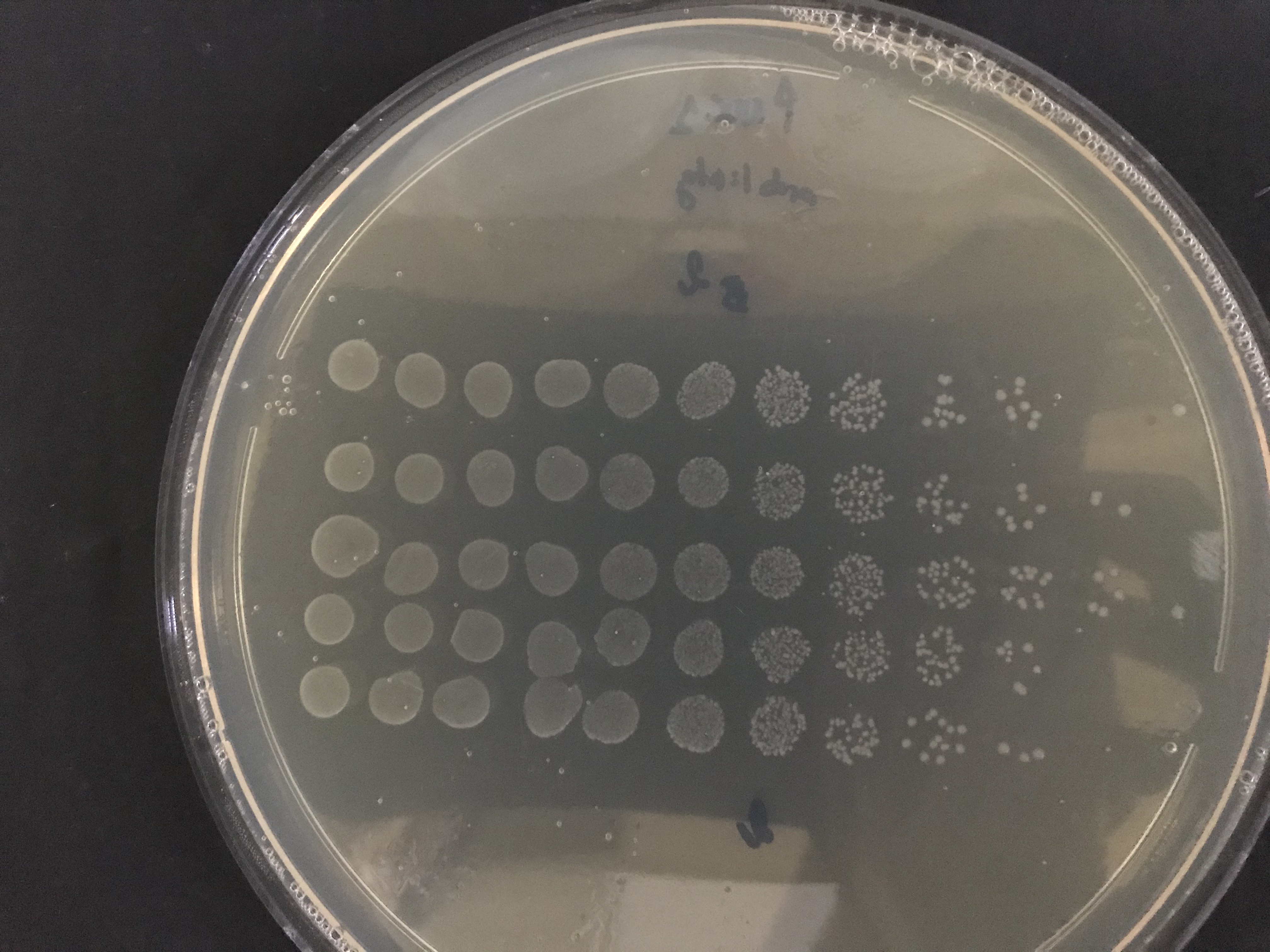

Supplement: Figure 2—source data 1. [file elife-82437-fig2-data1.zip › Figure 2- source data 1/TseV1 arab_iptg.jpg]

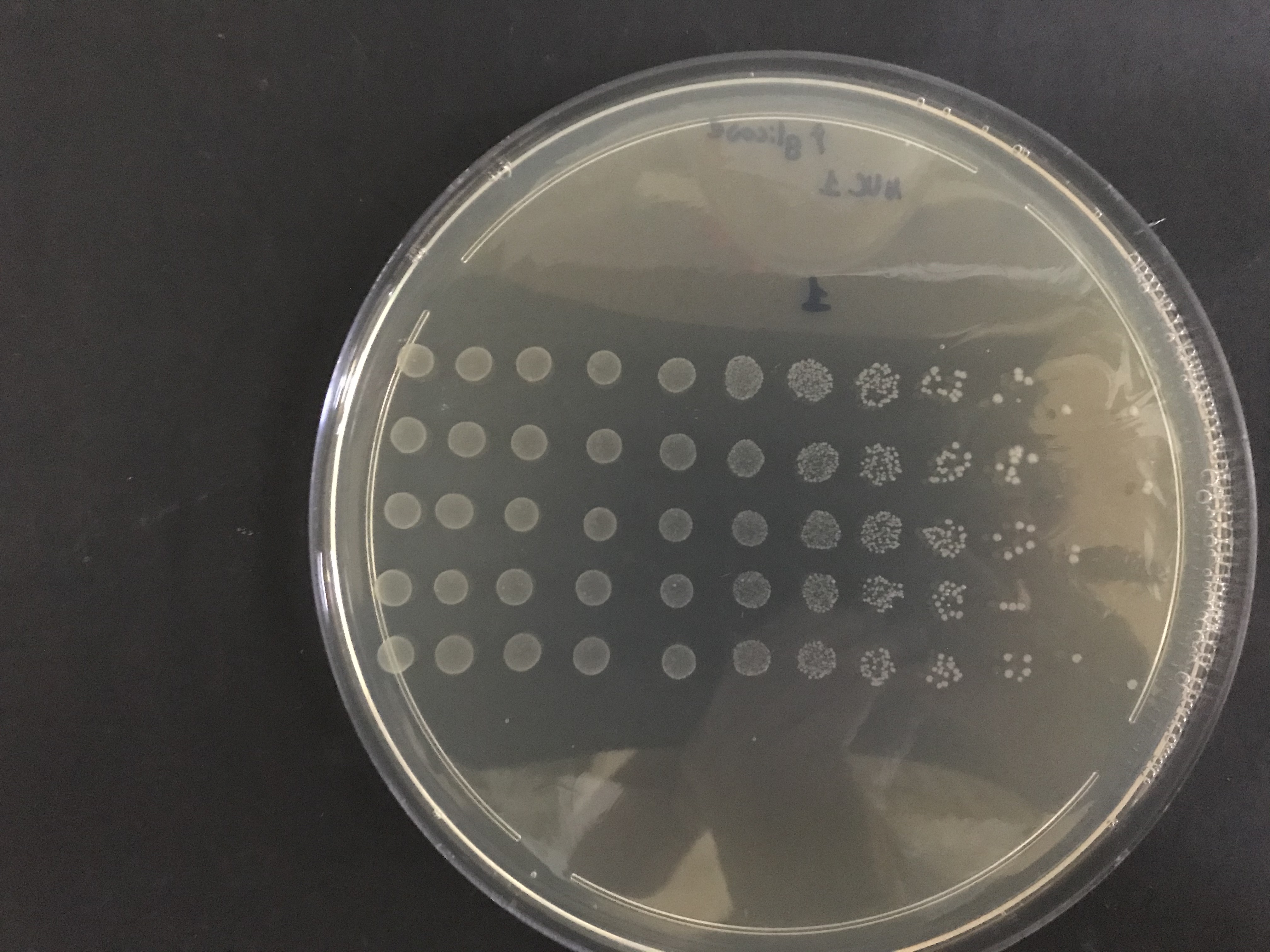

Supplement: Figure 2—source data 1. [file elife-82437-fig2-data1.zip › Figure 2- source data 1/TseV1 gluc.jpg]

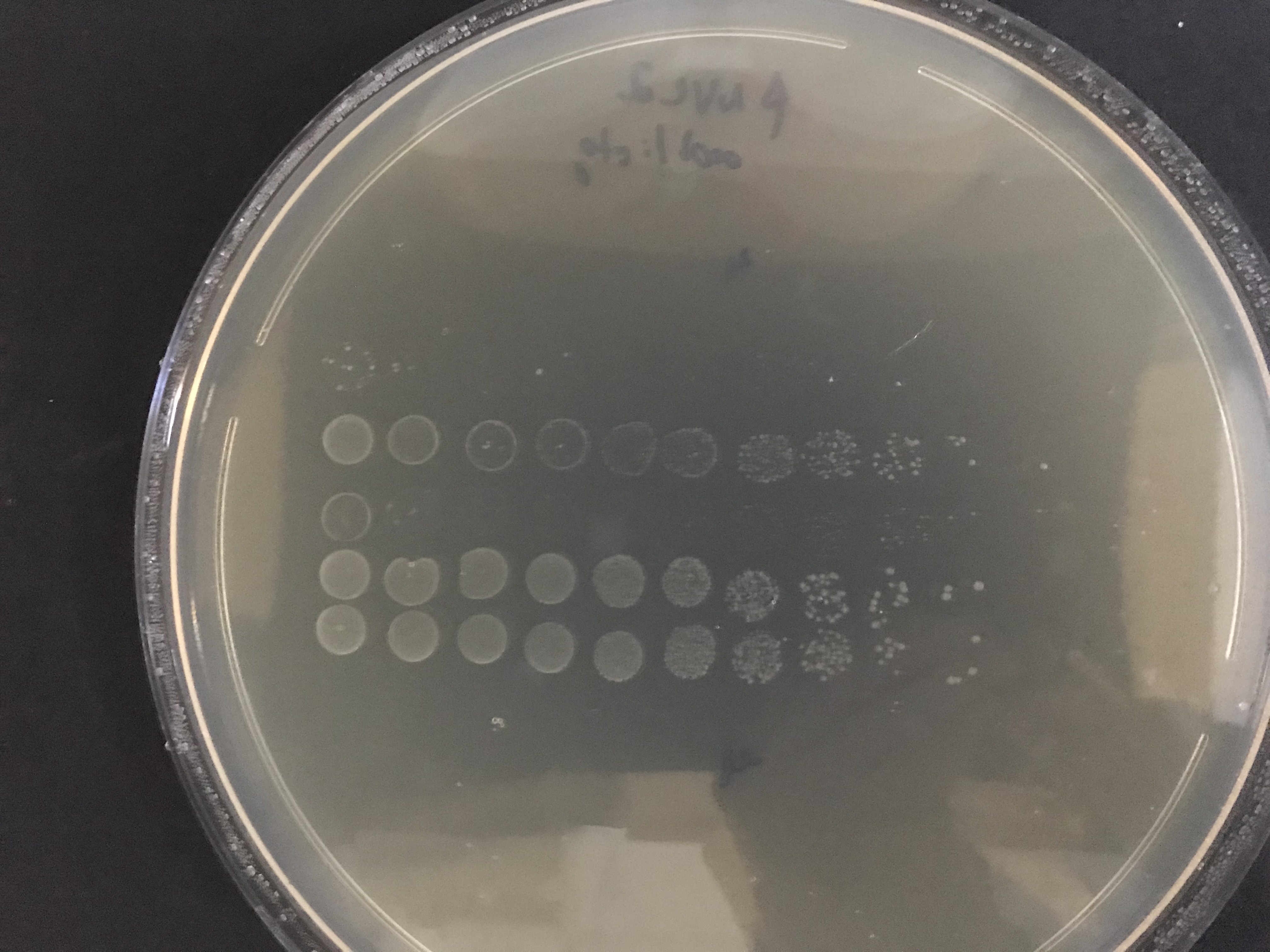

Supplement: Figure 2—source data 1. [file elife-82437-fig2-data1.zip › Figure 2- source data 1/TseV2 arab_iptg.jpg]

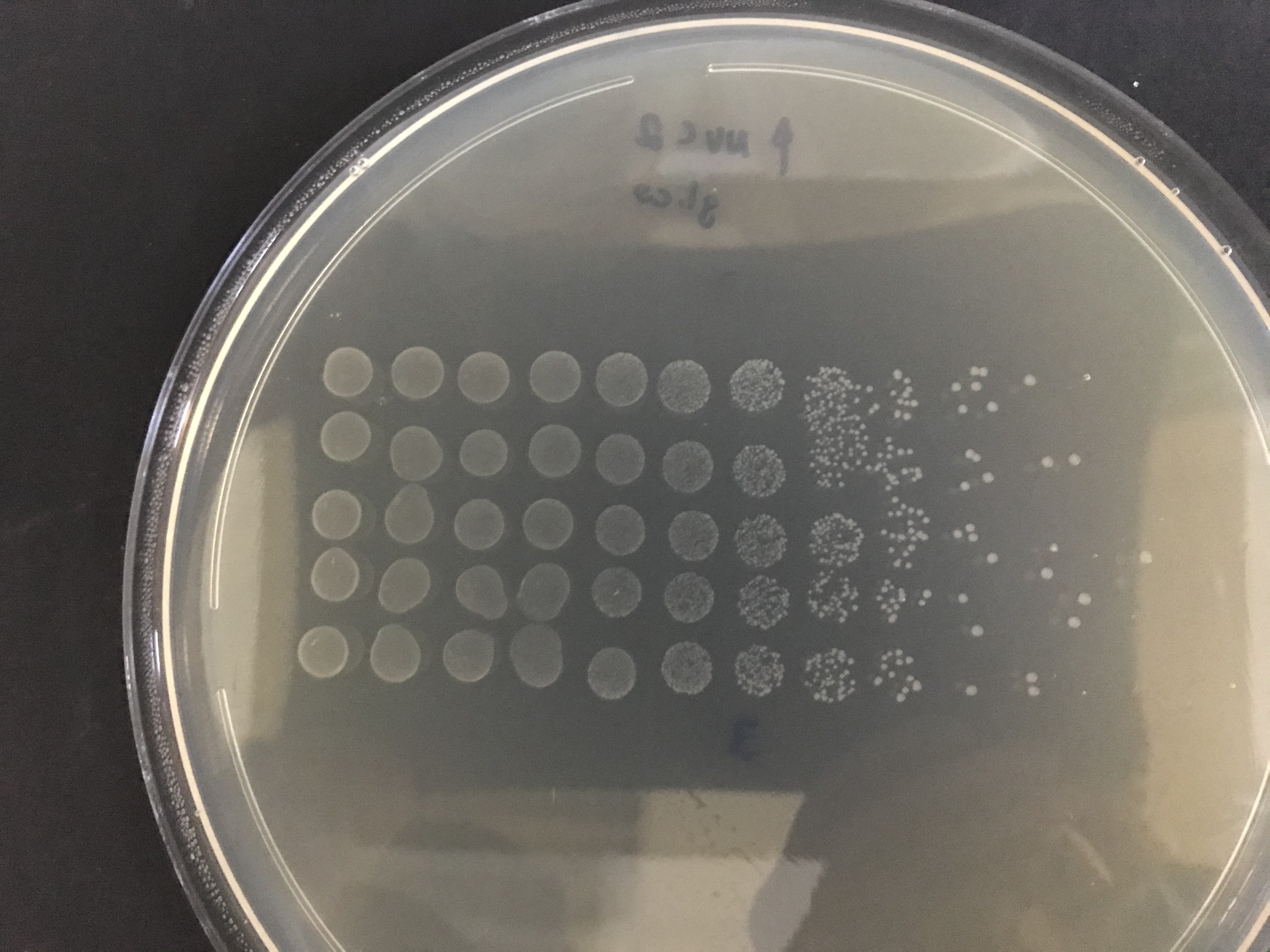

Supplement: Figure 2—source data 1. [file elife-82437-fig2-data1.zip › Figure 2- source data 1/TseV2 gluc.jpg]

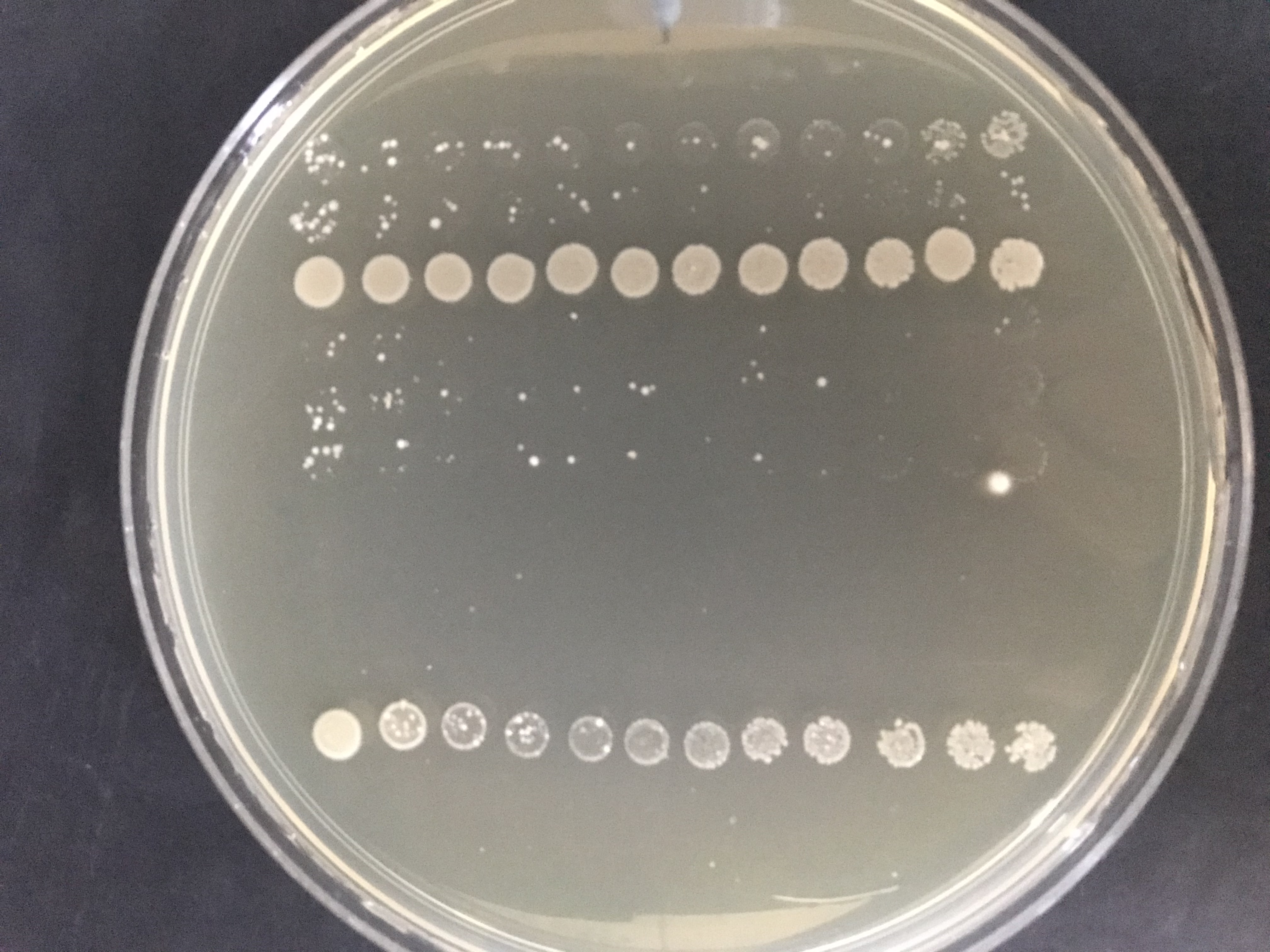

Supplement: Figure 2—source data 1. [file elife-82437-fig2-data1.zip › Figure 2- source data 1/TseV2_3 all imm arab_iptg.jpg]

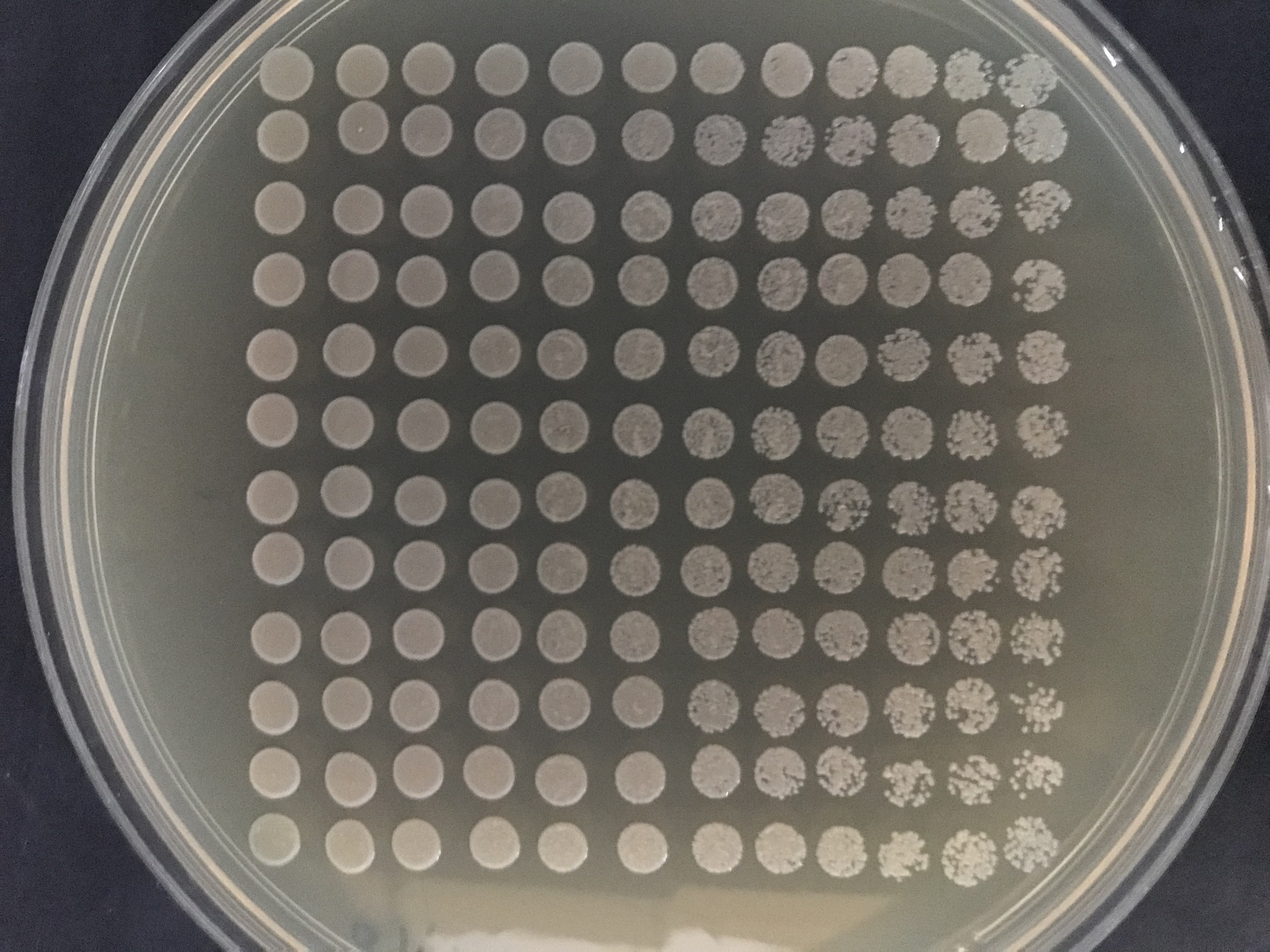

Supplement: Figure 2—source data 1. [file elife-82437-fig2-data1.zip › Figure 2- source data 1/TseV2_3 all imm gluc.jpg]

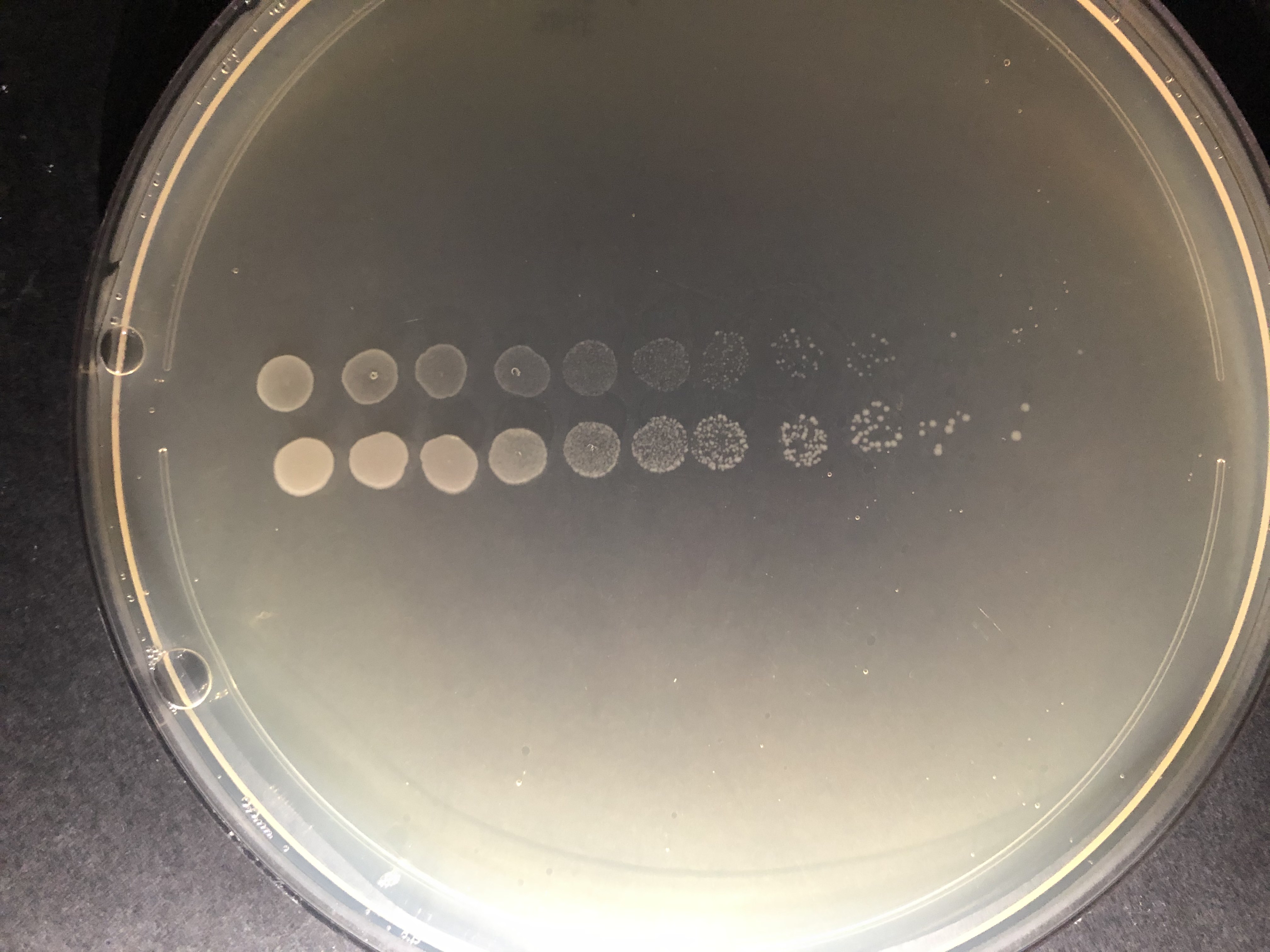

Supplement: Figure 2—source data 1. [file elife-82437-fig2-data1.zip › Figure 2- source data 1/TseV3 arab_iptg.jpg]

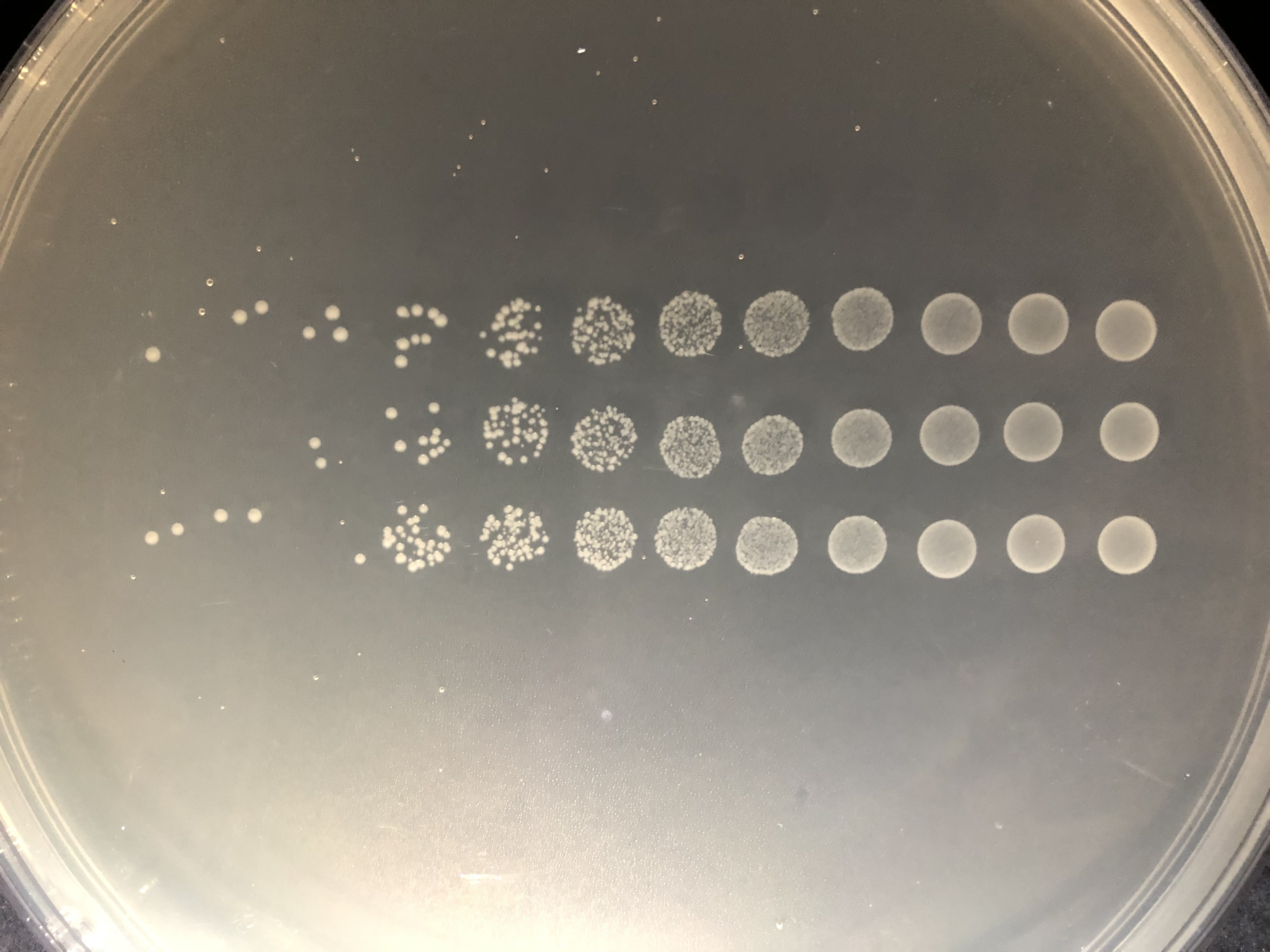

Supplement: Figure 2—source data 1. [file elife-82437-fig2-data1.zip › Figure 2- source data 1/TseV3 gluc.jpg]

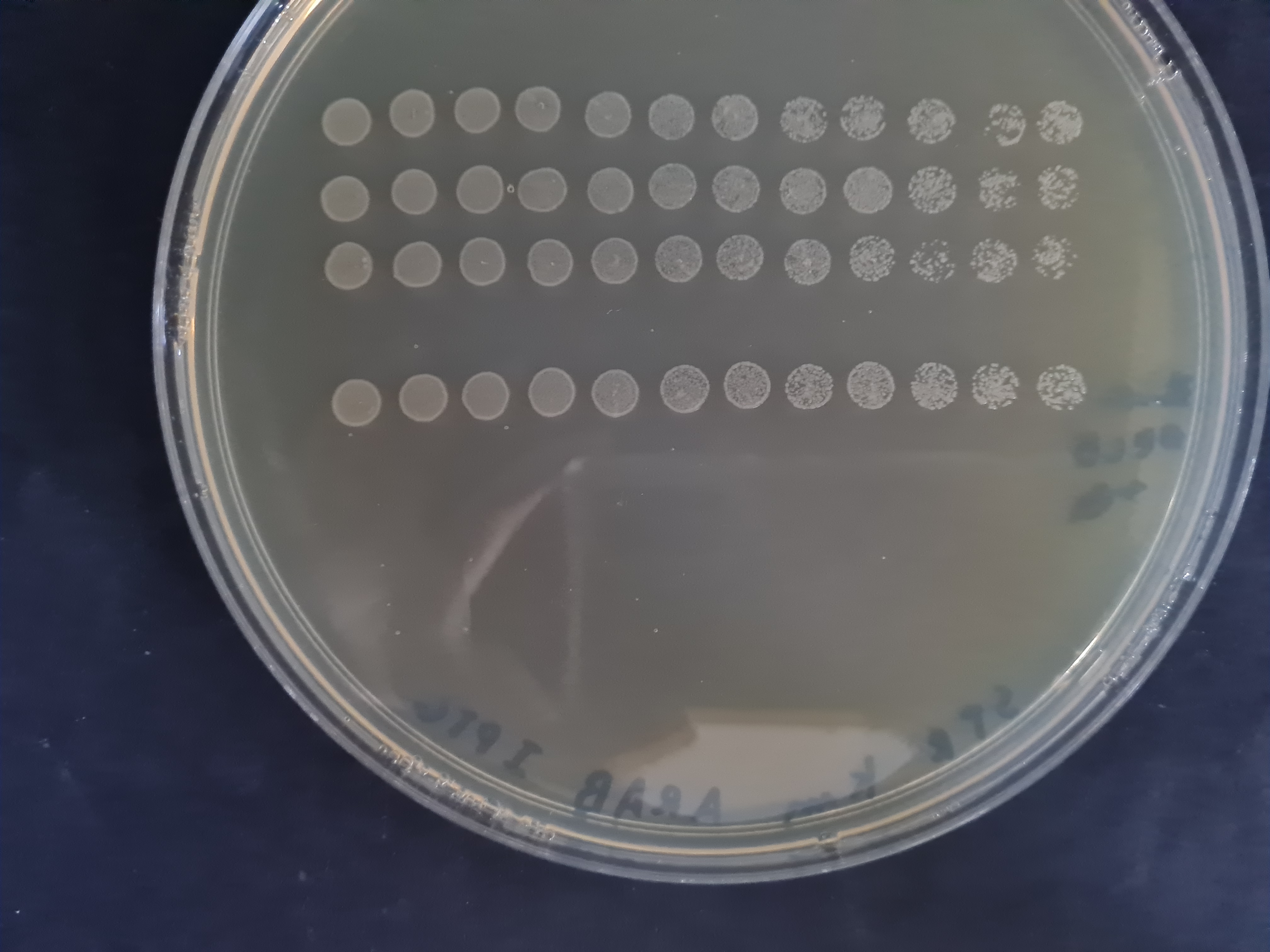

Supplement: Figure 2—source data 1. [file elife-82437-fig2-data1.zip › Figure 2- source data 1/TseV4 arab_iptg.jpg]

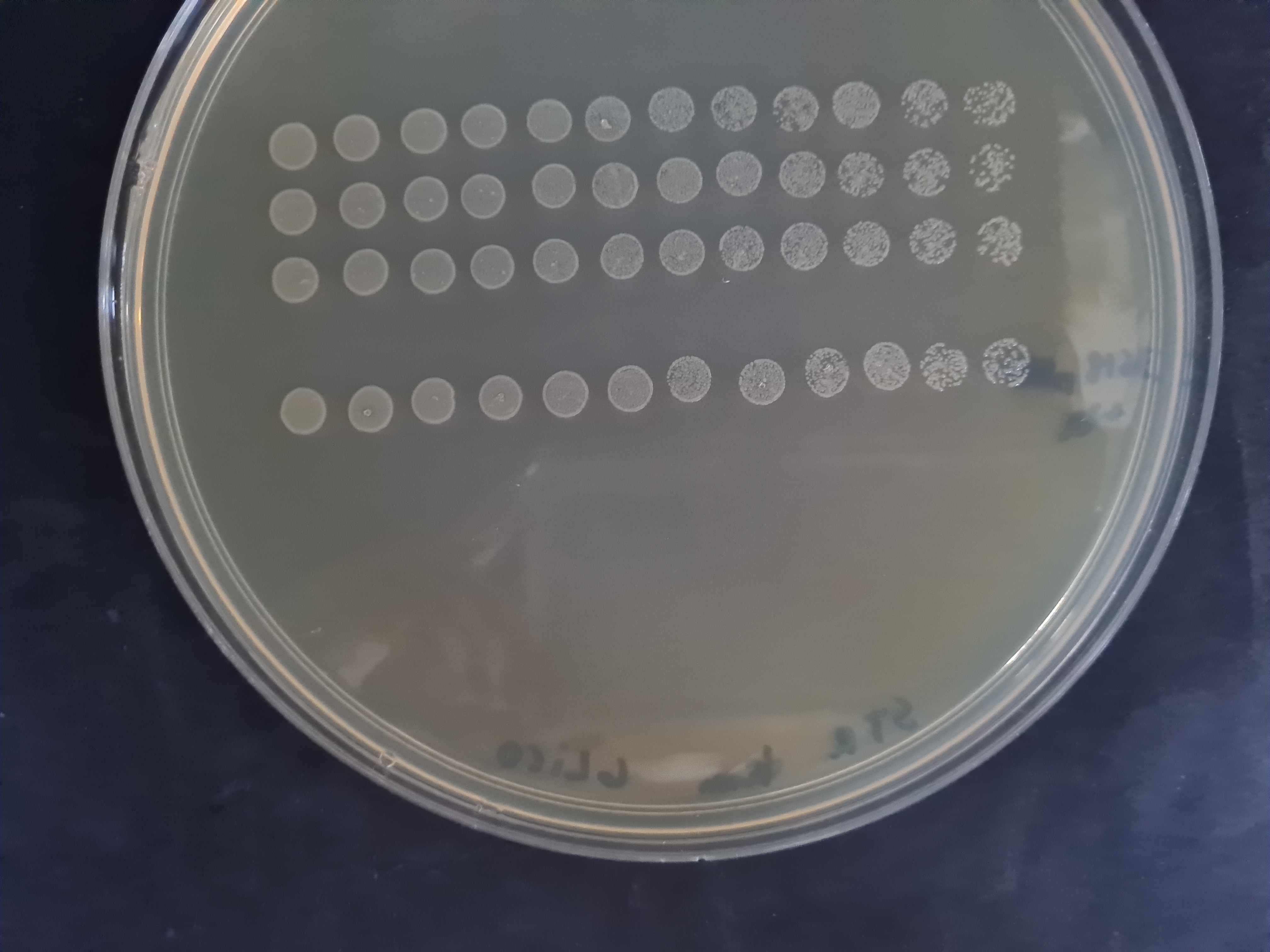

Supplement: Figure 2—source data 1. [file elife-82437-fig2-data1.zip › Figure 2- source data 1/TseV4 gluc.jpg]

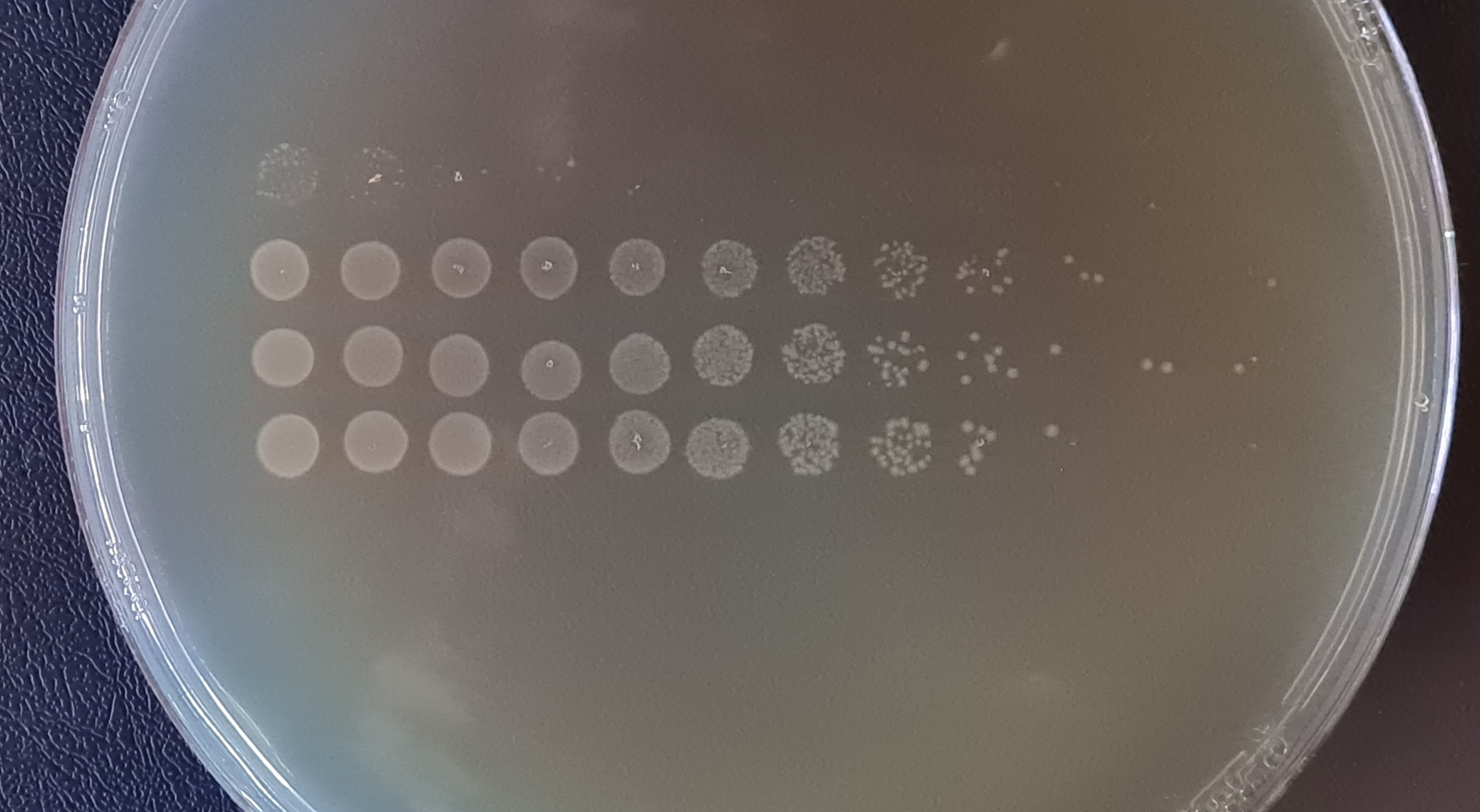

Supplement: Figure 3—source data 2. [file elife-82437-fig3-data2.zip › Figure 3- source data 2/TseV2 arab-iptg.jpg]

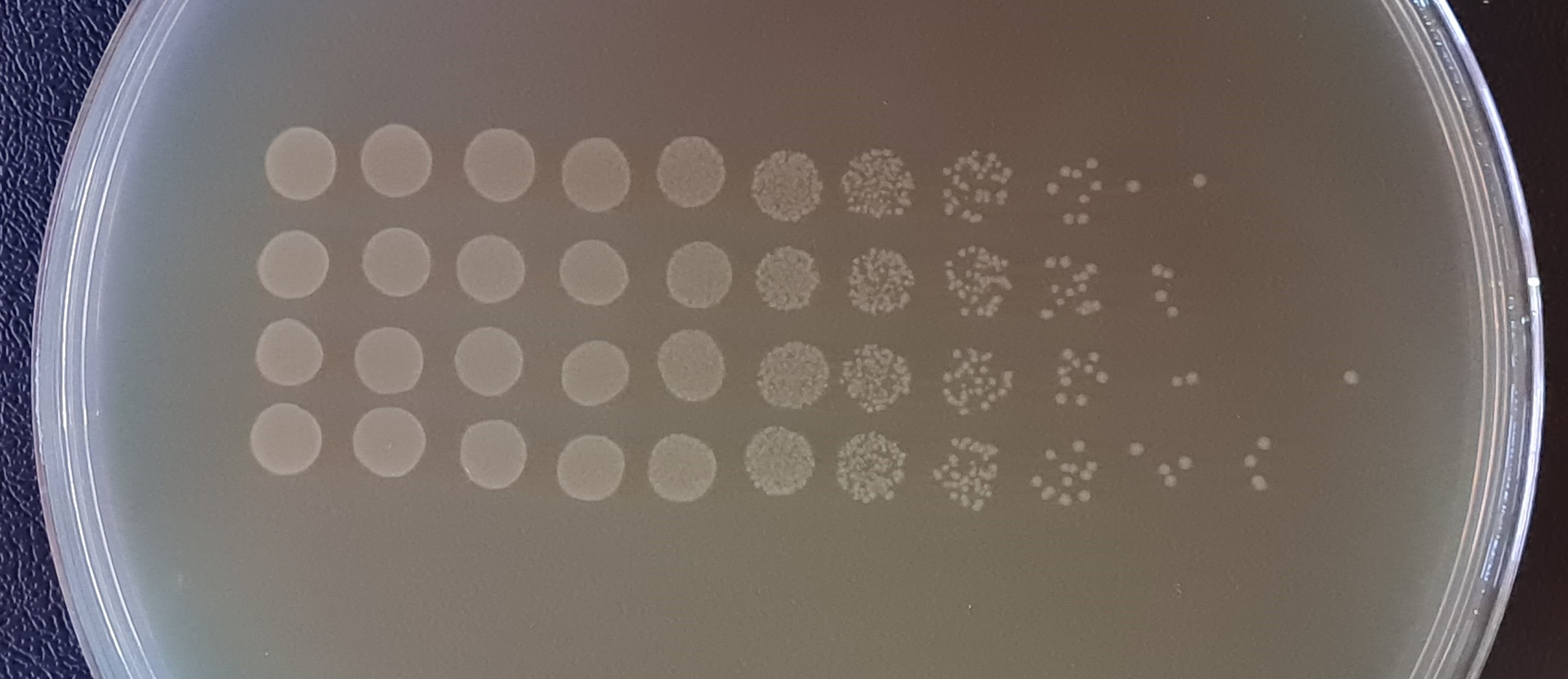

Supplement: Figure 3—source data 2. [file elife-82437-fig3-data2.zip › Figure 3- source data 2/TseV2 gluc.jpg]

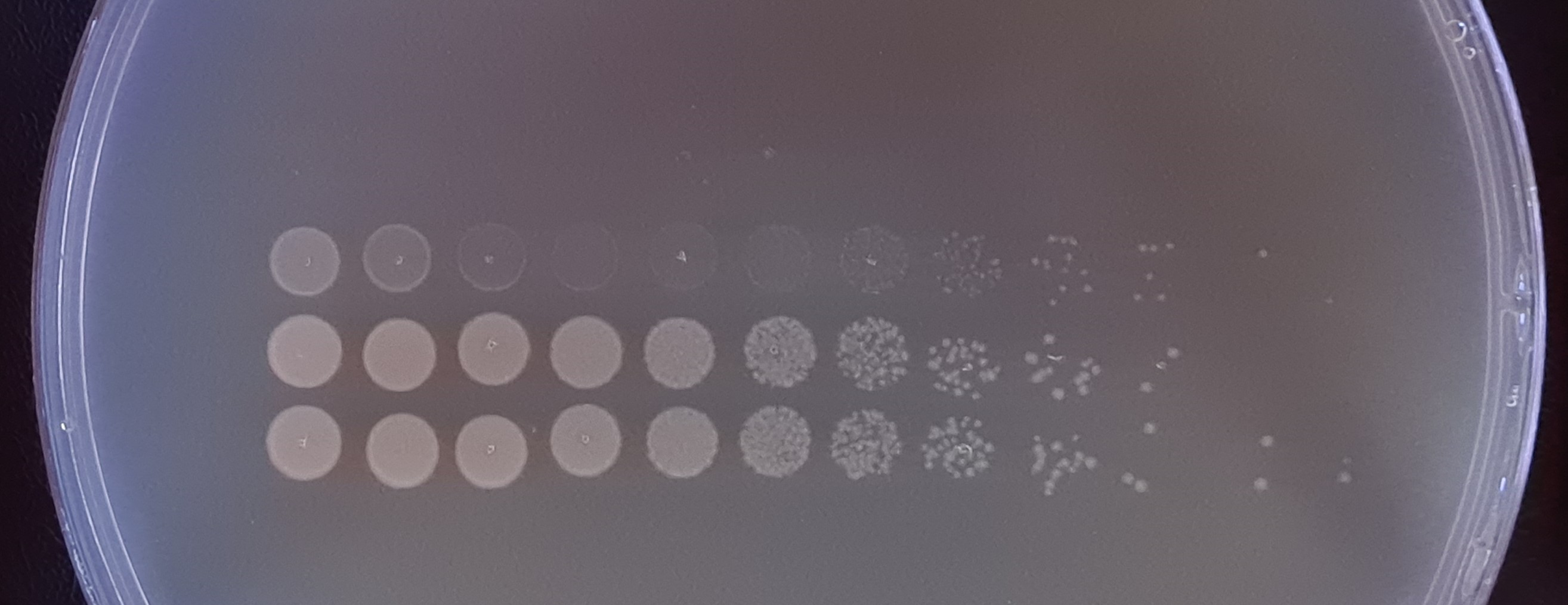

Supplement: Figure 3—source data 2. [file elife-82437-fig3-data2.zip › Figure 3- source data 2/TseV3 arab-iptg.jpg]

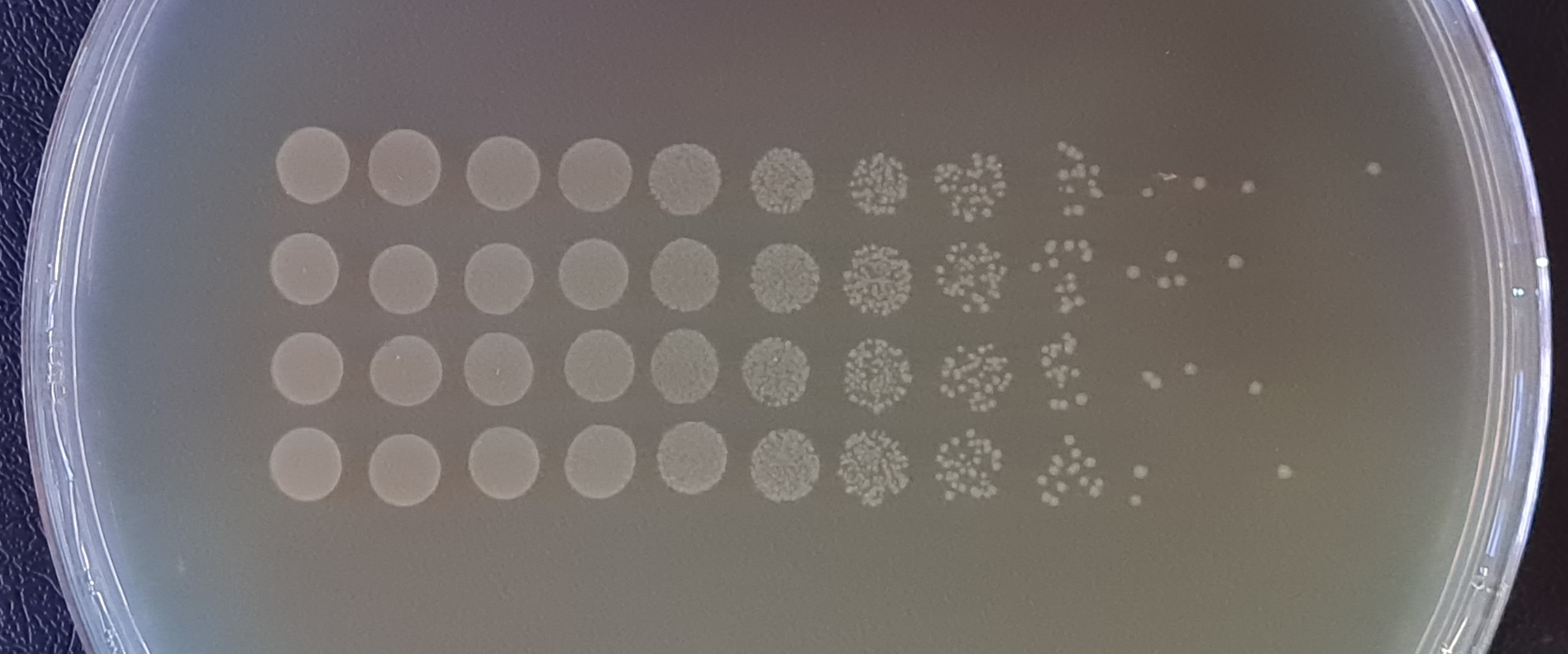

Supplement: Figure 3—source data 2. [file elife-82437-fig3-data2.zip › Figure 3- source data 2/TseV3 gluc.jpg]

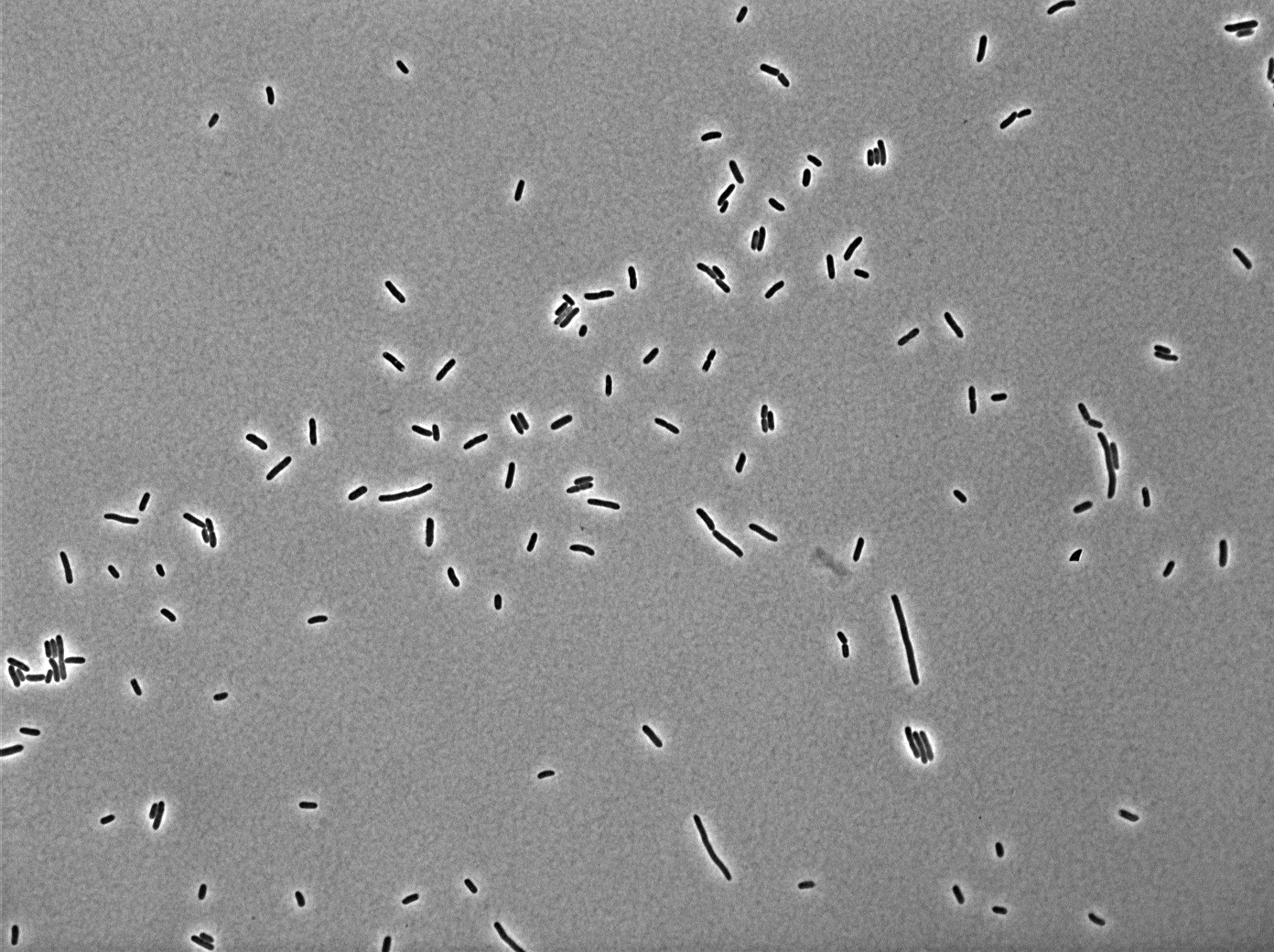

Supplement: Figure 4—source data 2. — The files can be opened in ImageJ. [file elife-82437-fig4-data2.zip › Figure 4- source data 2/TseV2 DAPI images/pBRA_TseV2_Ara.lif - Image001.tif]

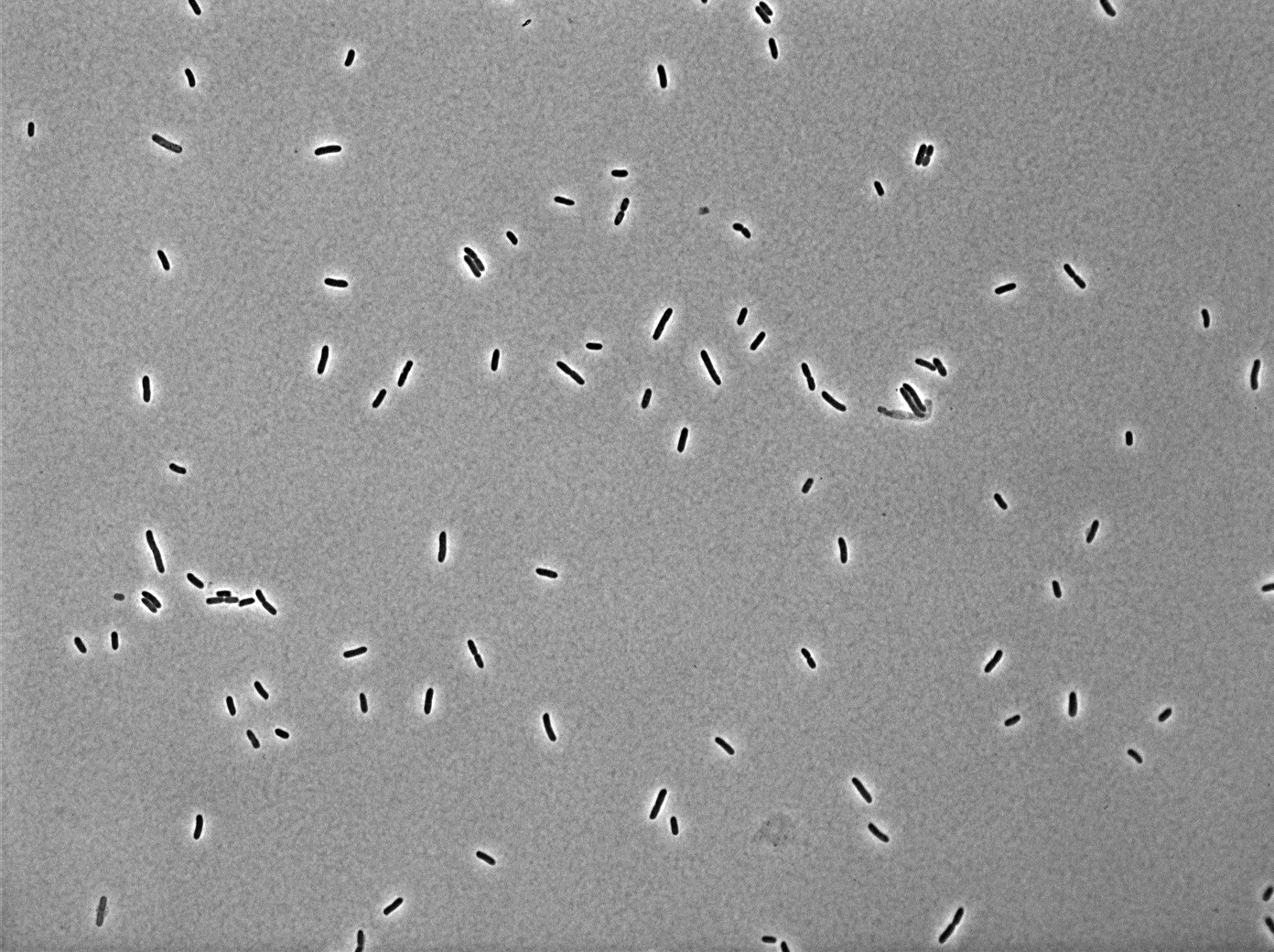

Supplement: Figure 4—source data 2. — The files can be opened in ImageJ. [file elife-82437-fig4-data2.zip › Figure 4- source data 2/TseV2 DAPI images/pBRA_TseV2_Ara.lif - Image002.tif]

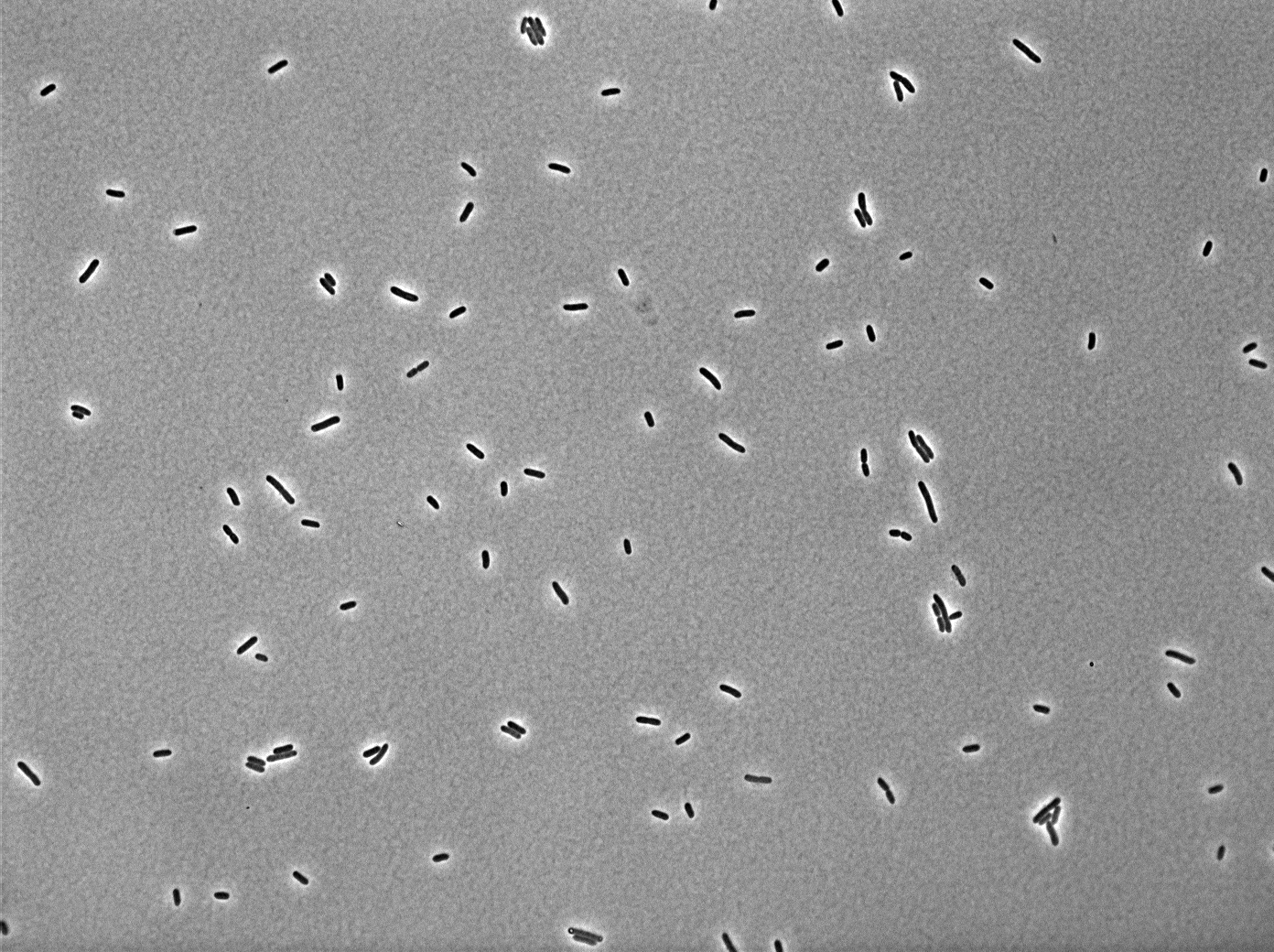

Supplement: Figure 4—source data 2. — The files can be opened in ImageJ. [file elife-82437-fig4-data2.zip › Figure 4- source data 2/TseV2 DAPI images/pBRA_TseV2_Ara.lif - Image003.tif]

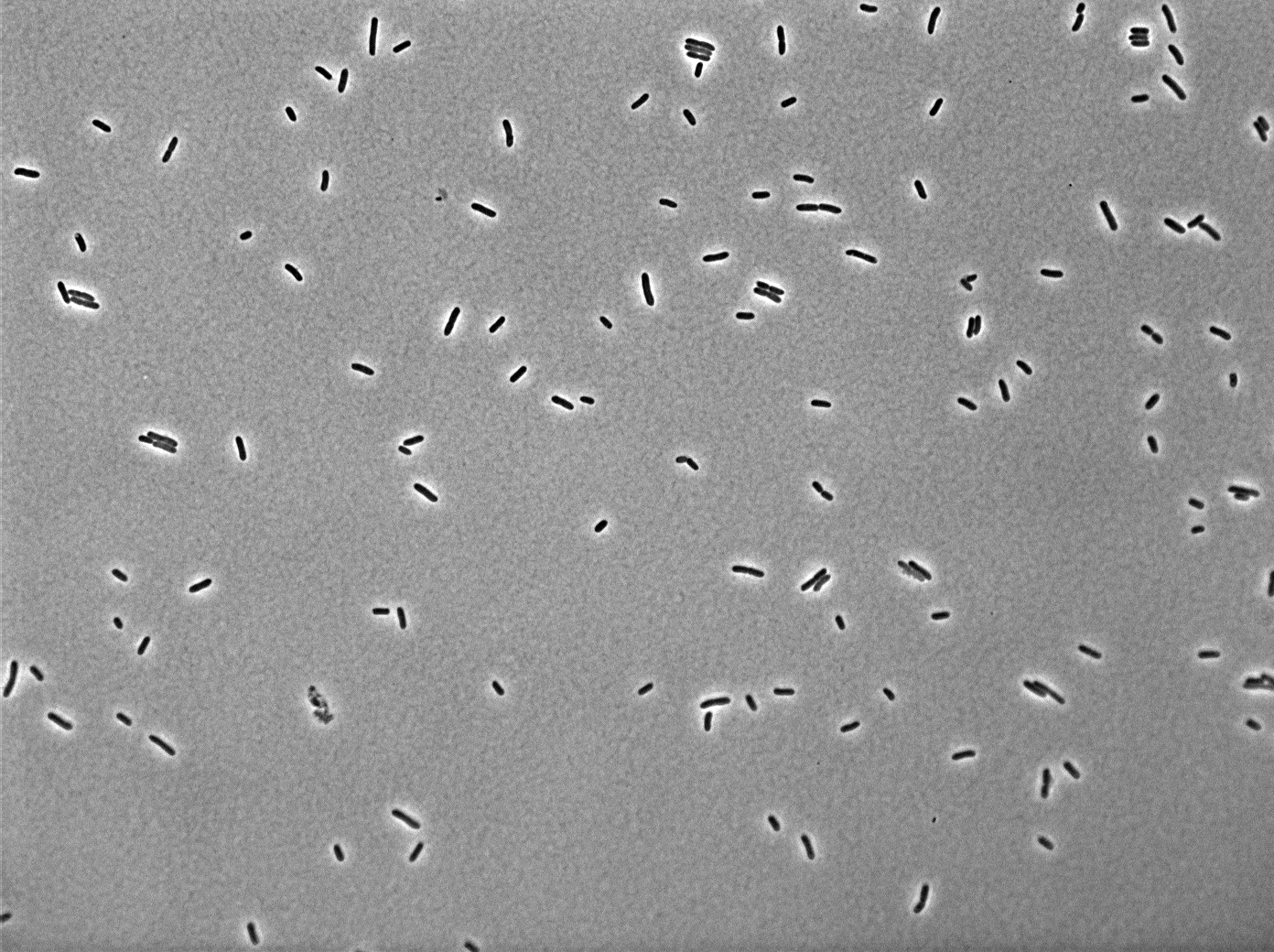

Supplement: Figure 4—source data 2. — The files can be opened in ImageJ. [file elife-82437-fig4-data2.zip › Figure 4- source data 2/TseV2 DAPI images/pBRA_TseV2_Ara.lif - Image004.tif]

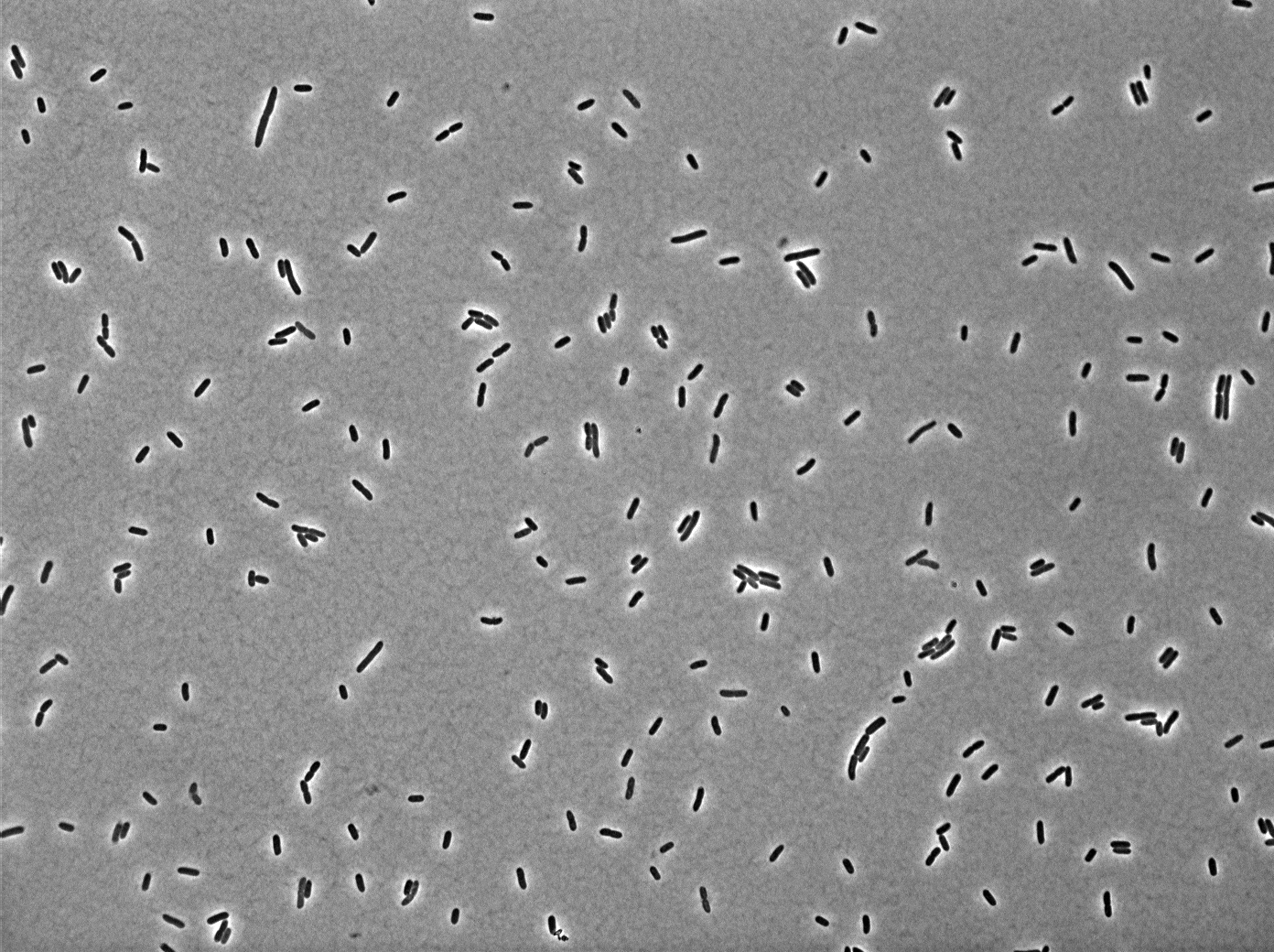

Supplement: Figure 4—source data 2. — The files can be opened in ImageJ. [file elife-82437-fig4-data2.zip › Figure 4- source data 2/TseV2 DAPI images/pBRA_TseV2_Gluc.lif - Image001.tif]

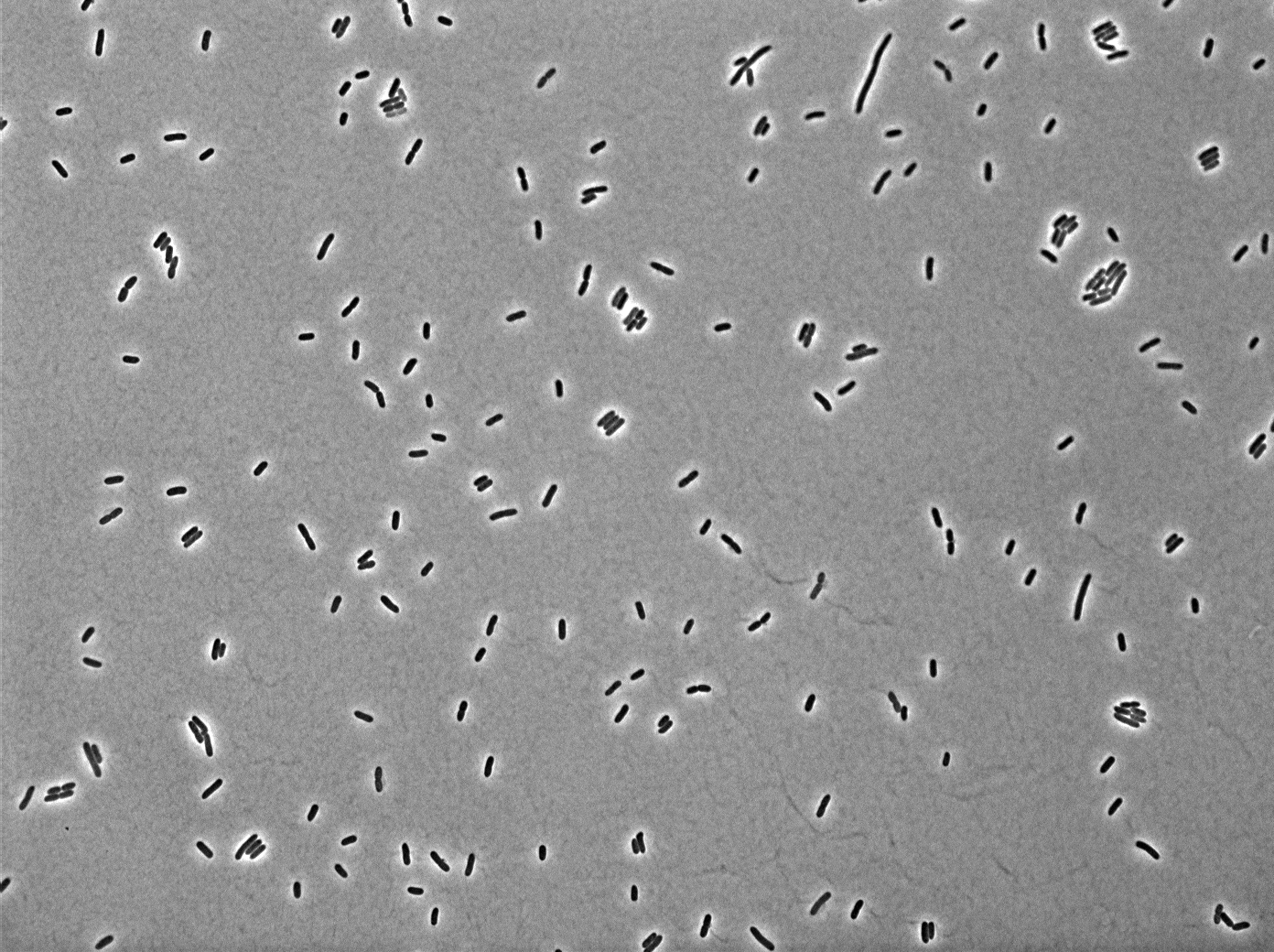

Supplement: Figure 4—source data 2. — The files can be opened in ImageJ. [file elife-82437-fig4-data2.zip › Figure 4- source data 2/TseV2 DAPI images/pBRA_TseV2_Gluc.lif - Image002.tif]

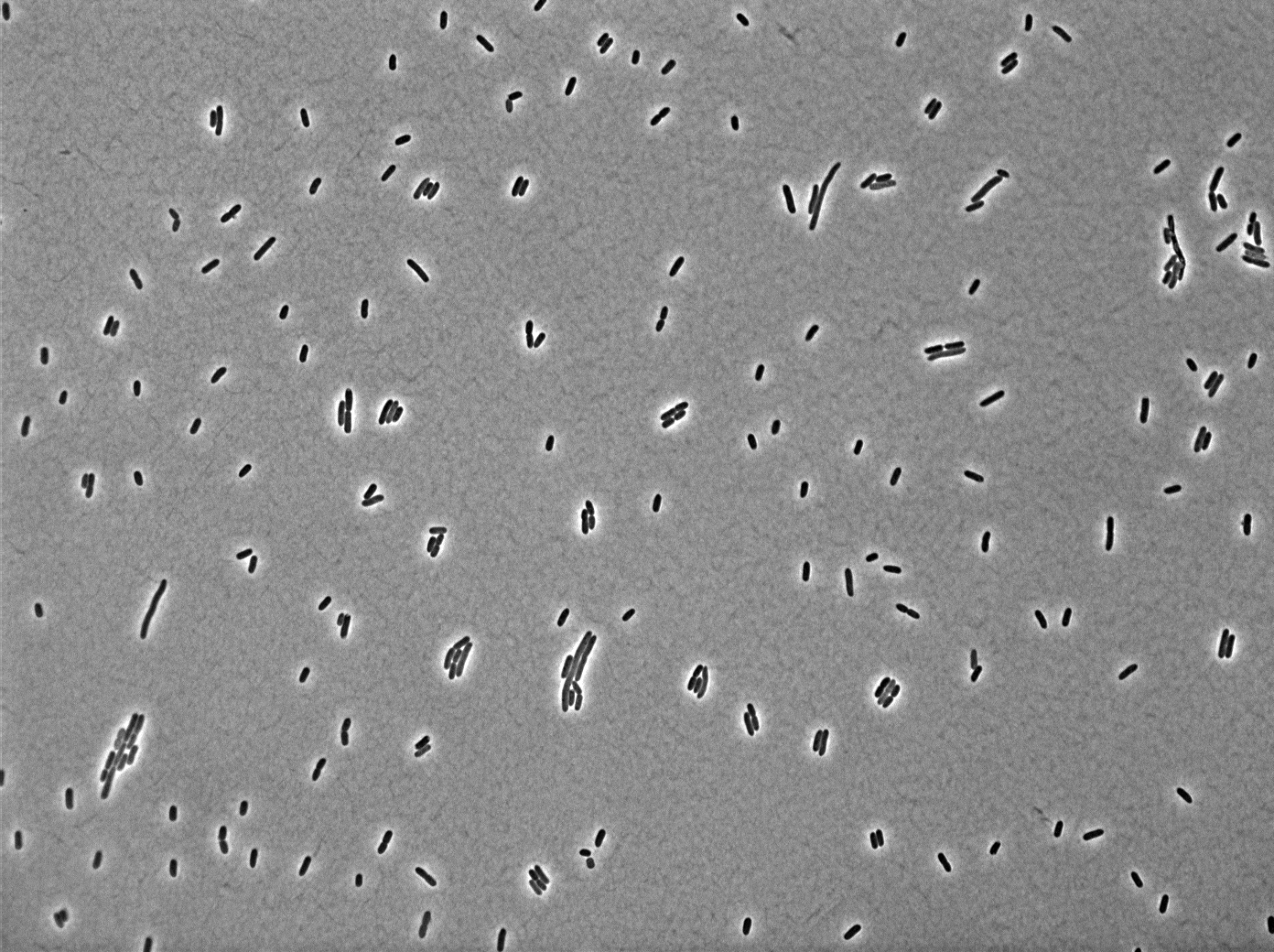

Supplement: Figure 4—source data 2. — The files can be opened in ImageJ. [file elife-82437-fig4-data2.zip › Figure 4- source data 2/TseV2 DAPI images/pBRA_TseV2_Gluc.lif - Image003.tif]

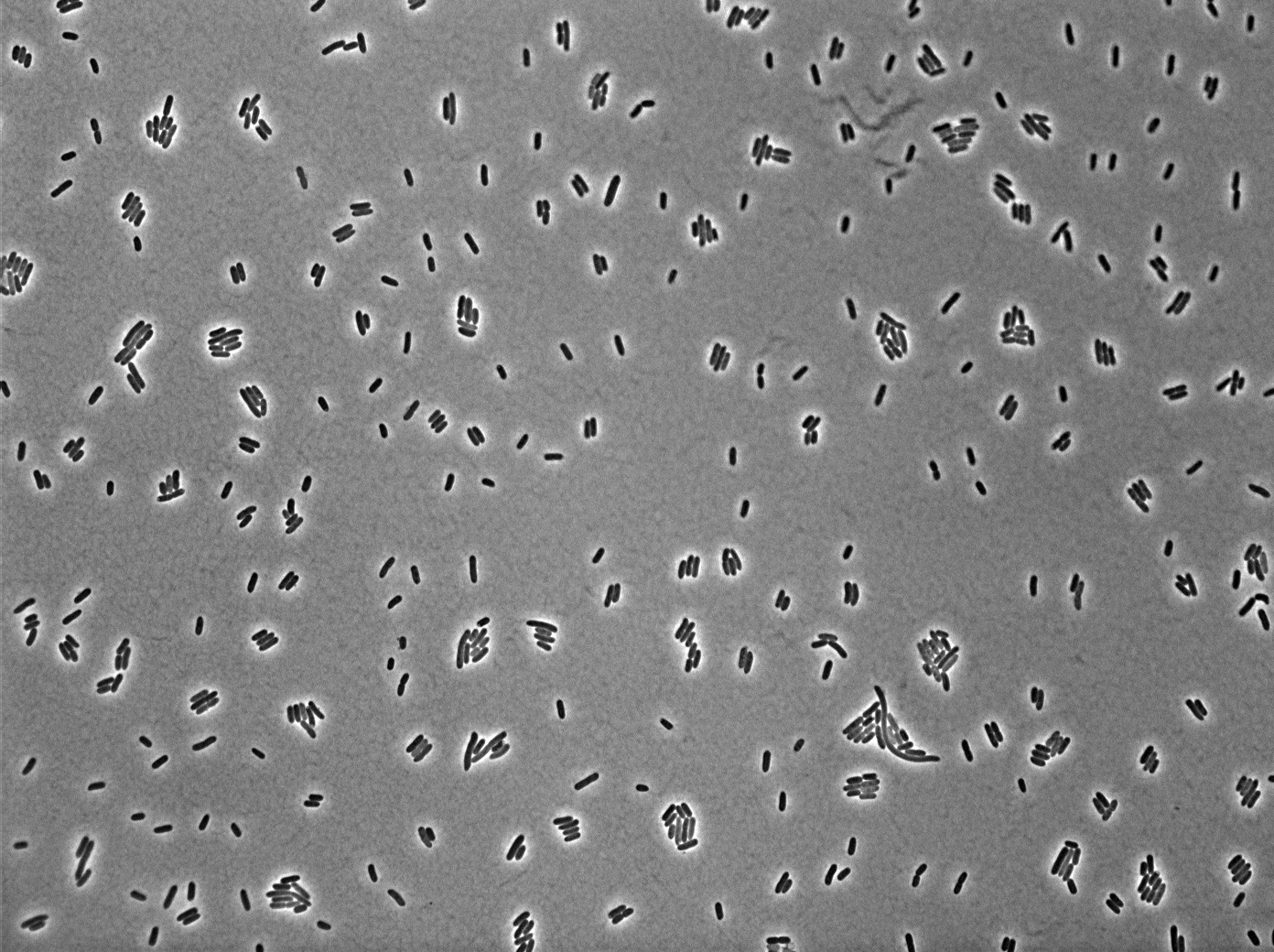

Supplement: Figure 4—source data 2. — The files can be opened in ImageJ. [file elife-82437-fig4-data2.zip › Figure 4- source data 2/TseV2 DAPI images/pBRA_TseV2_Gluc.lif - Image004.tif]

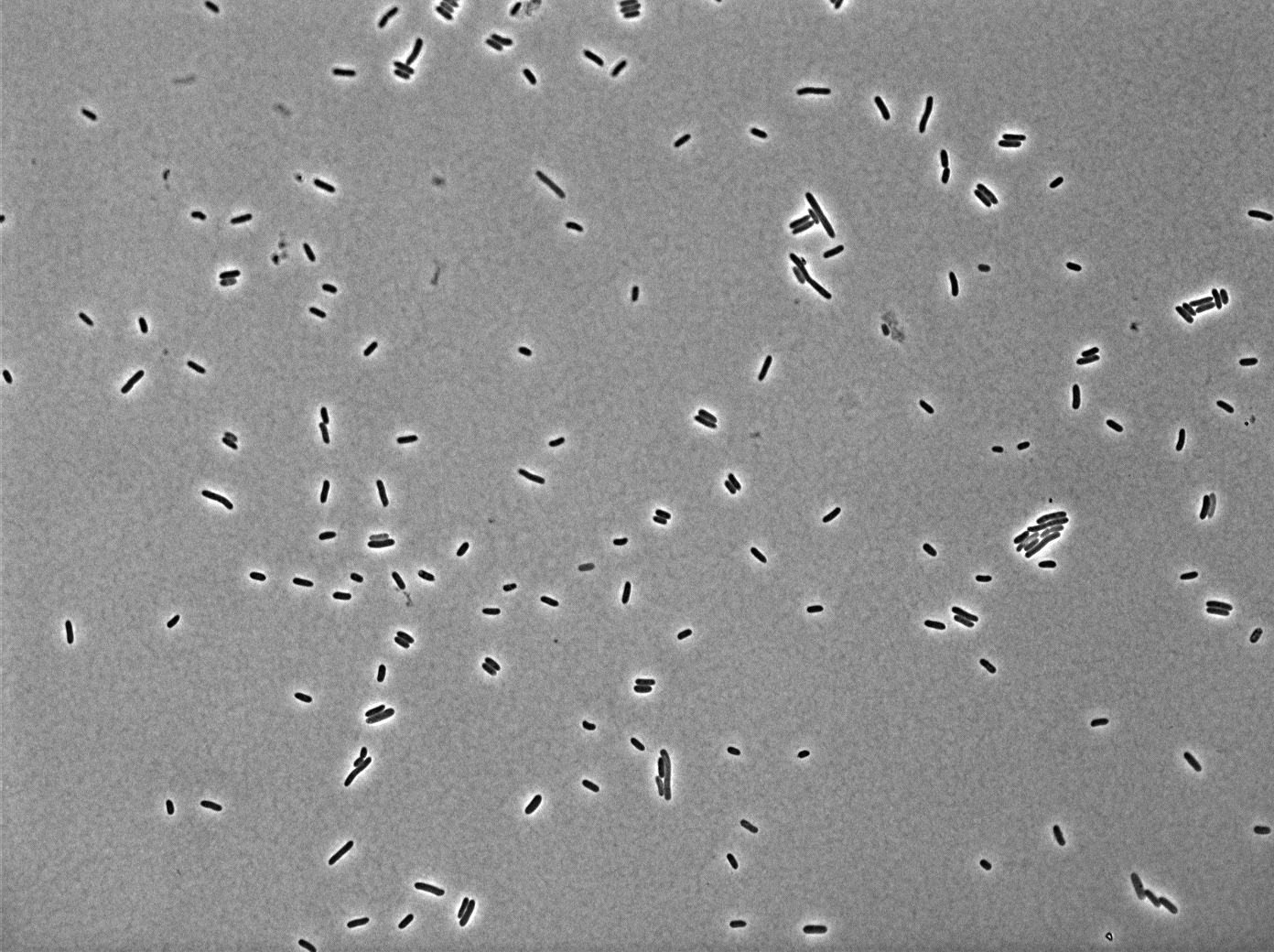

Supplement: Figure 4—source data 2. — The files can be opened in ImageJ. [file elife-82437-fig4-data2.zip › Figure 4- source data 2/TseV3 DAPI images/pBRA_TseV3_Ara.lif - Image001.tif]

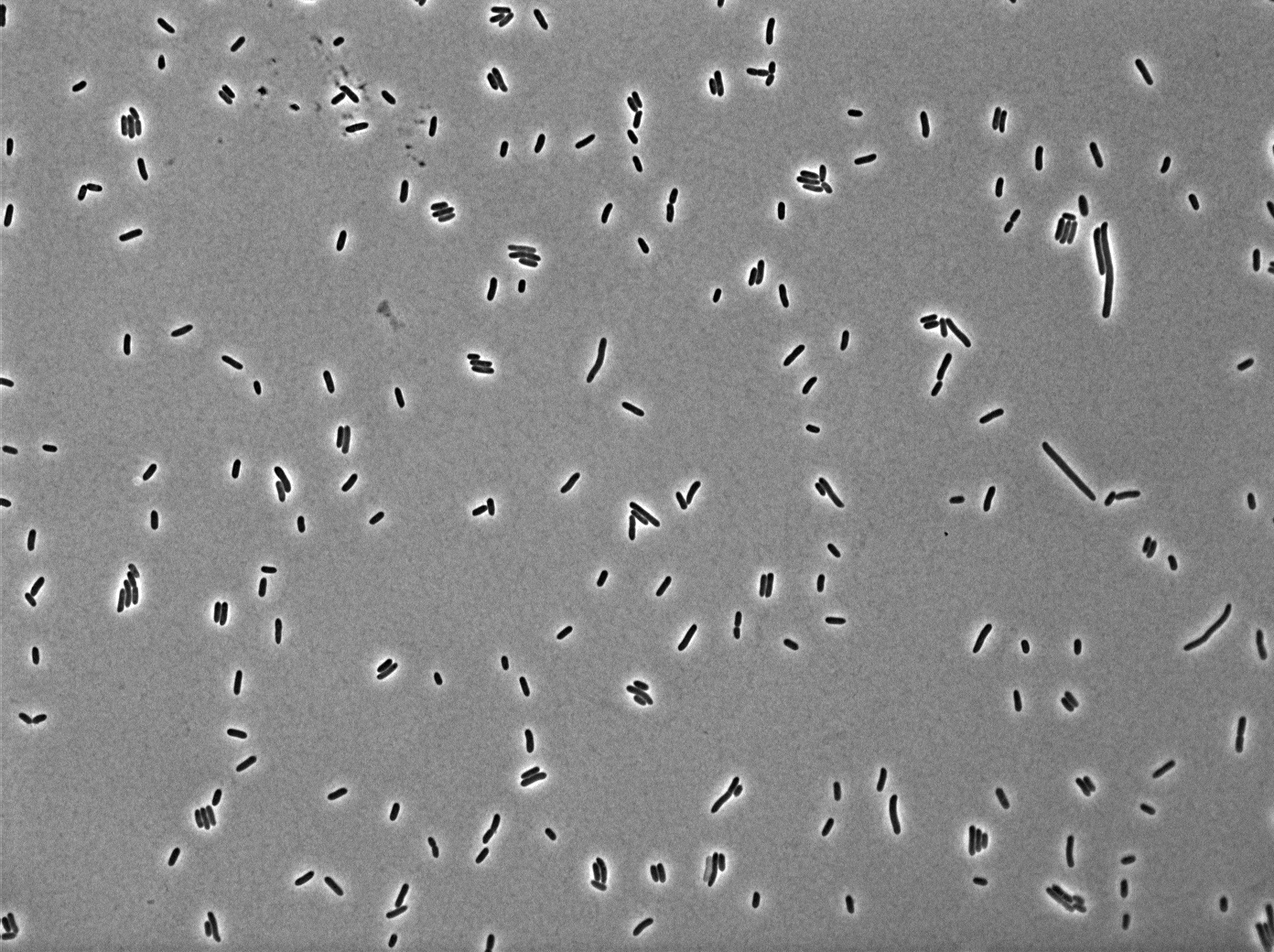

Supplement: Figure 4—source data 2. — The files can be opened in ImageJ. [file elife-82437-fig4-data2.zip › Figure 4- source data 2/TseV3 DAPI images/pBRA_Tsev3_Ara.lif - Image002.tif]

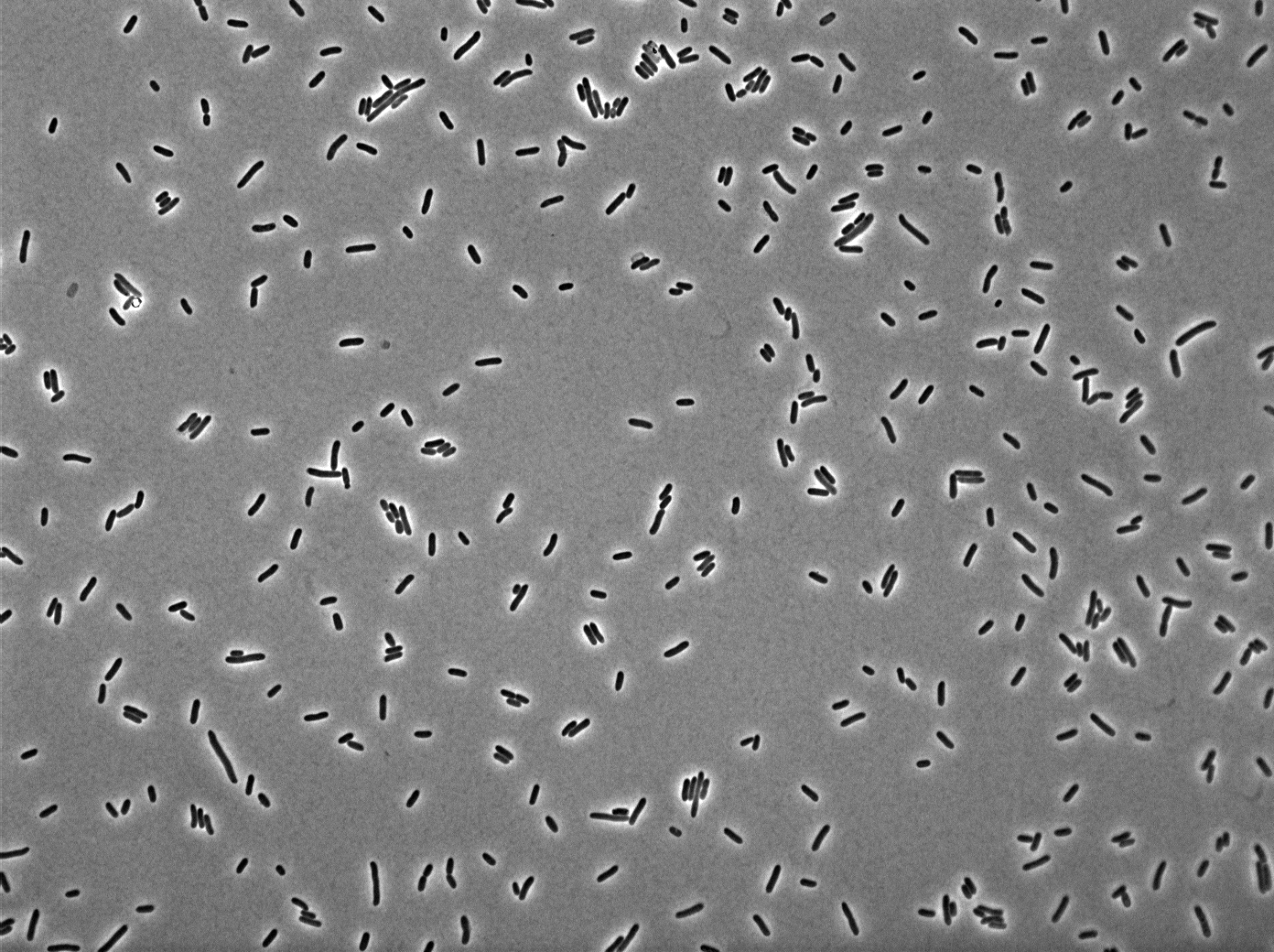

Supplement: Figure 4—source data 2. — The files can be opened in ImageJ. [file elife-82437-fig4-data2.zip › Figure 4- source data 2/TseV3 DAPI images/pBRA_TseV3_Ara.lif - Image003.tif]

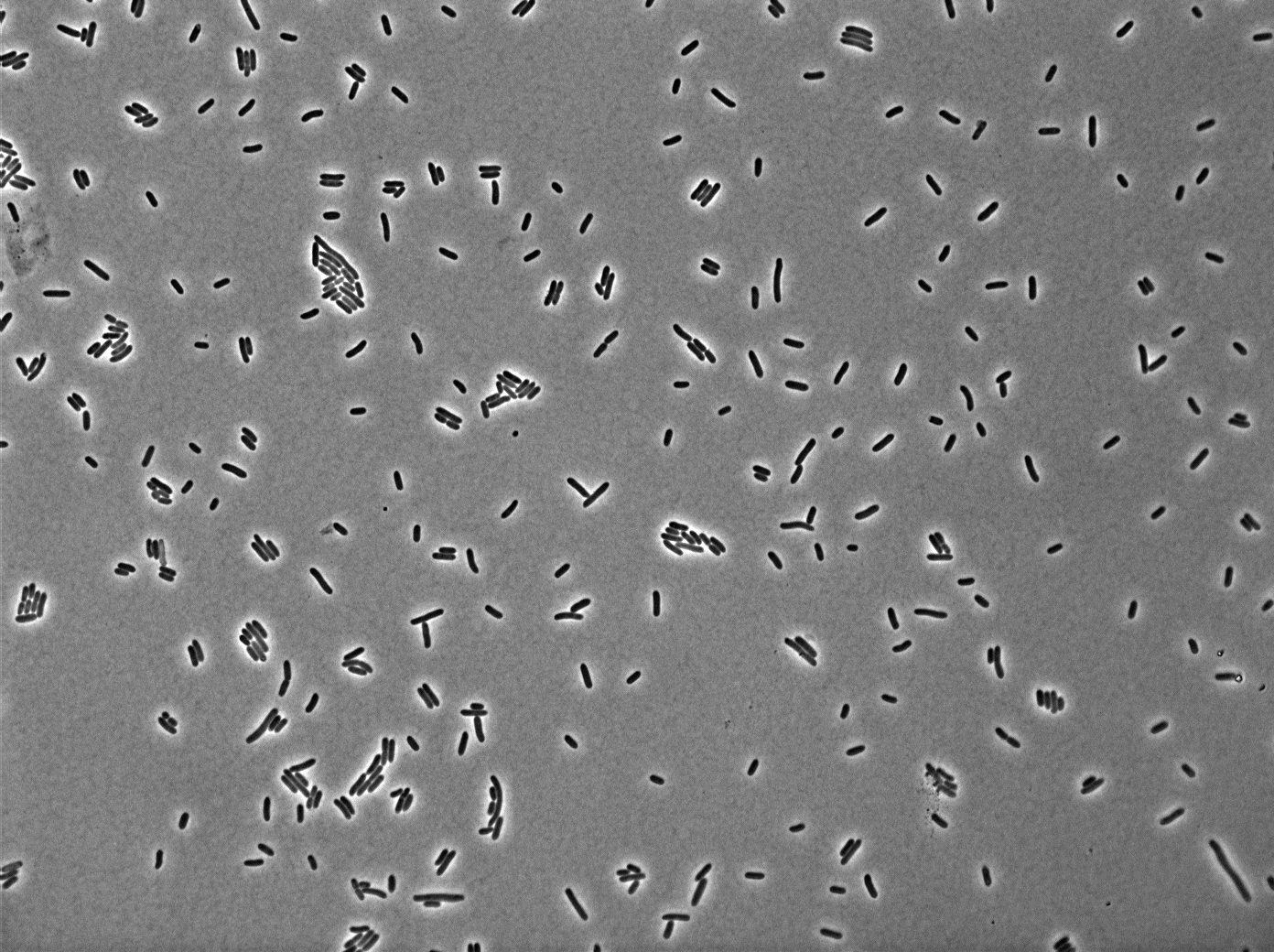

Supplement: Figure 4—source data 2. — The files can be opened in ImageJ. [file elife-82437-fig4-data2.zip › Figure 4- source data 2/TseV3 DAPI images/pBRA_TseV3_Ara.lif - Image004.tif]

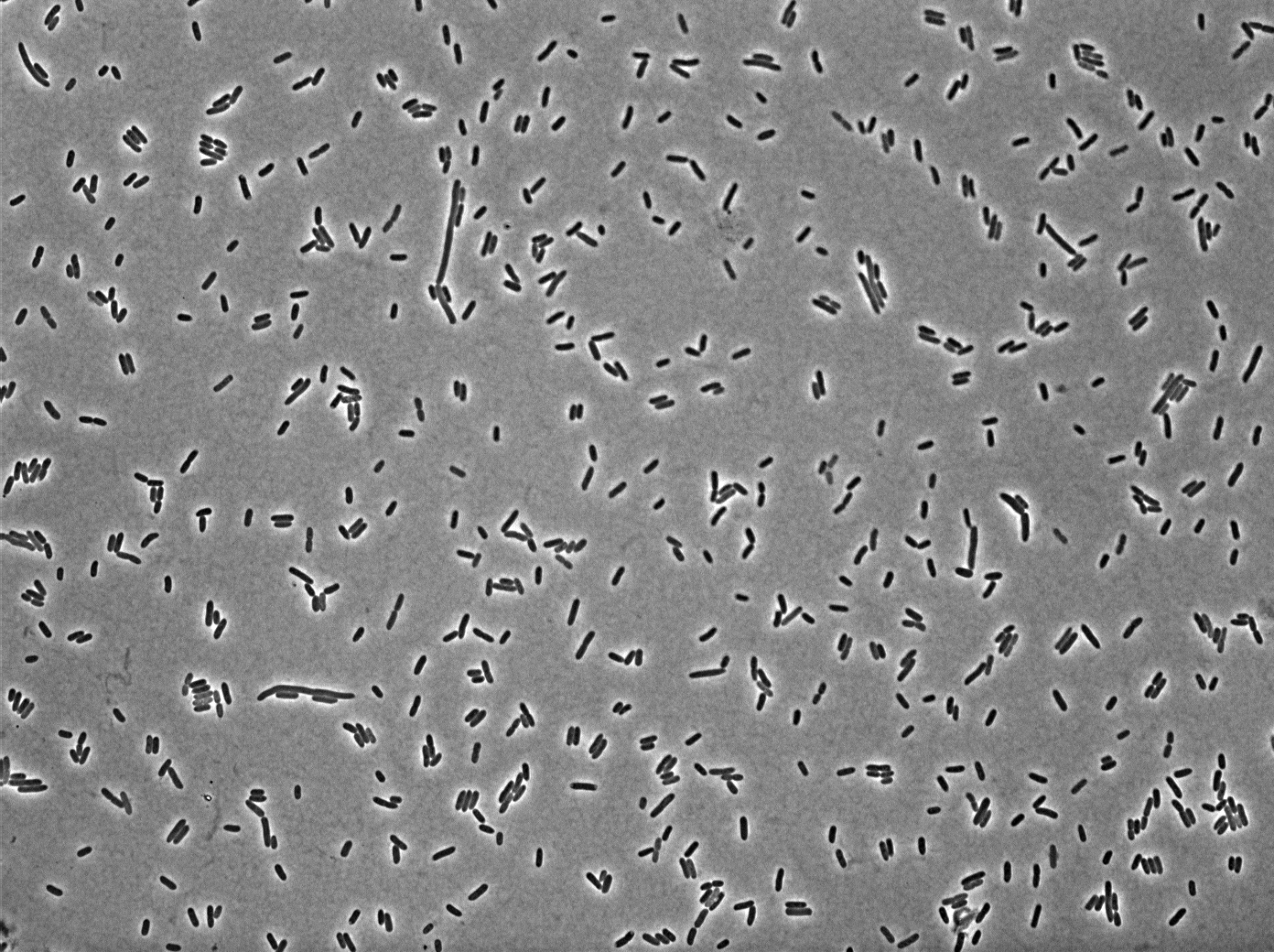

Supplement: Figure 4—source data 2. — The files can be opened in ImageJ. [file elife-82437-fig4-data2.zip › Figure 4- source data 2/TseV3 DAPI images/pBRA_TseV3_Gluc.lif - Image001.tif]

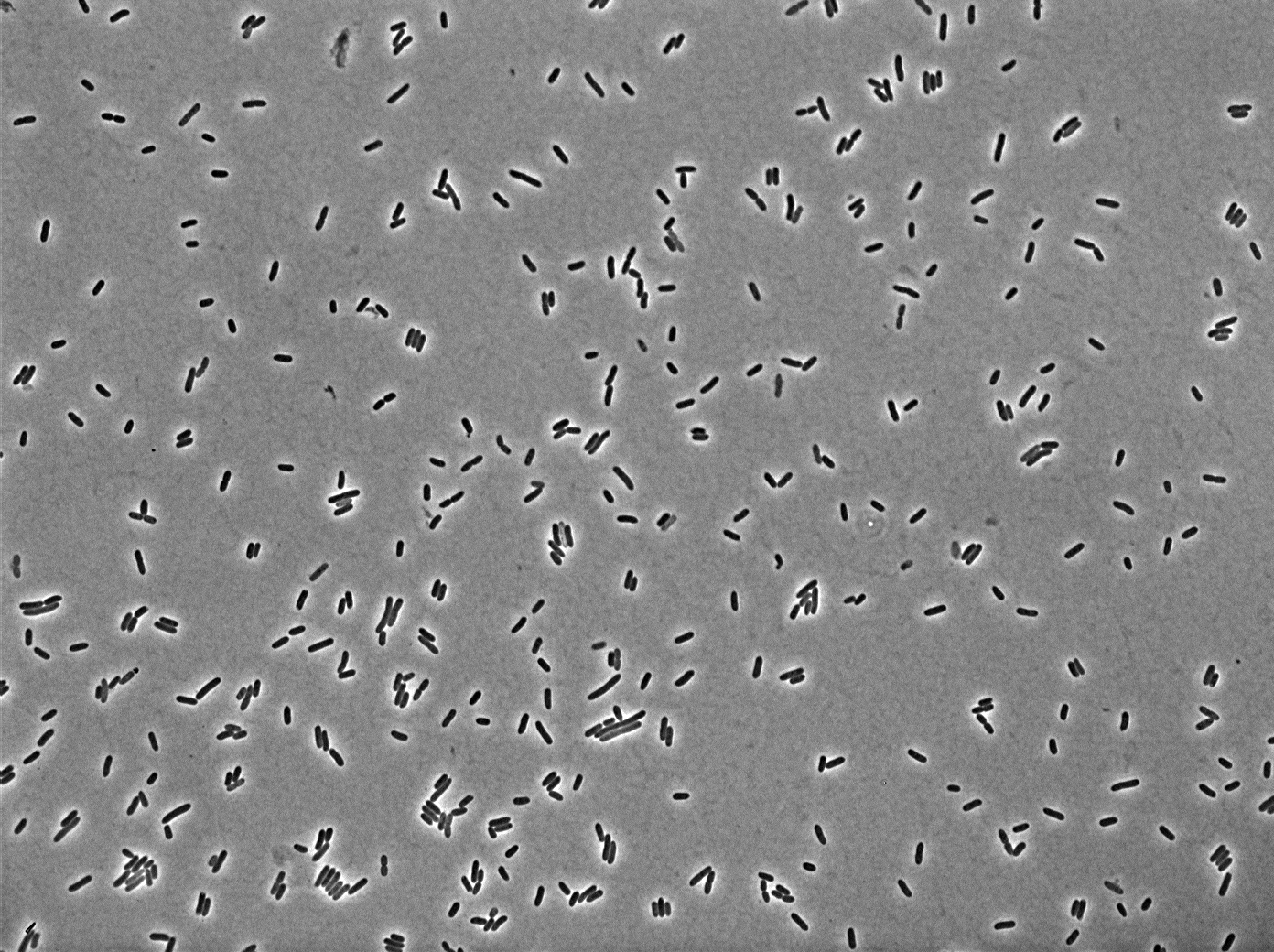

Supplement: Figure 4—source data 2. — The files can be opened in ImageJ. [file elife-82437-fig4-data2.zip › Figure 4- source data 2/TseV3 DAPI images/pBRA_TseV3_Gluc.lif - Image002.tif]

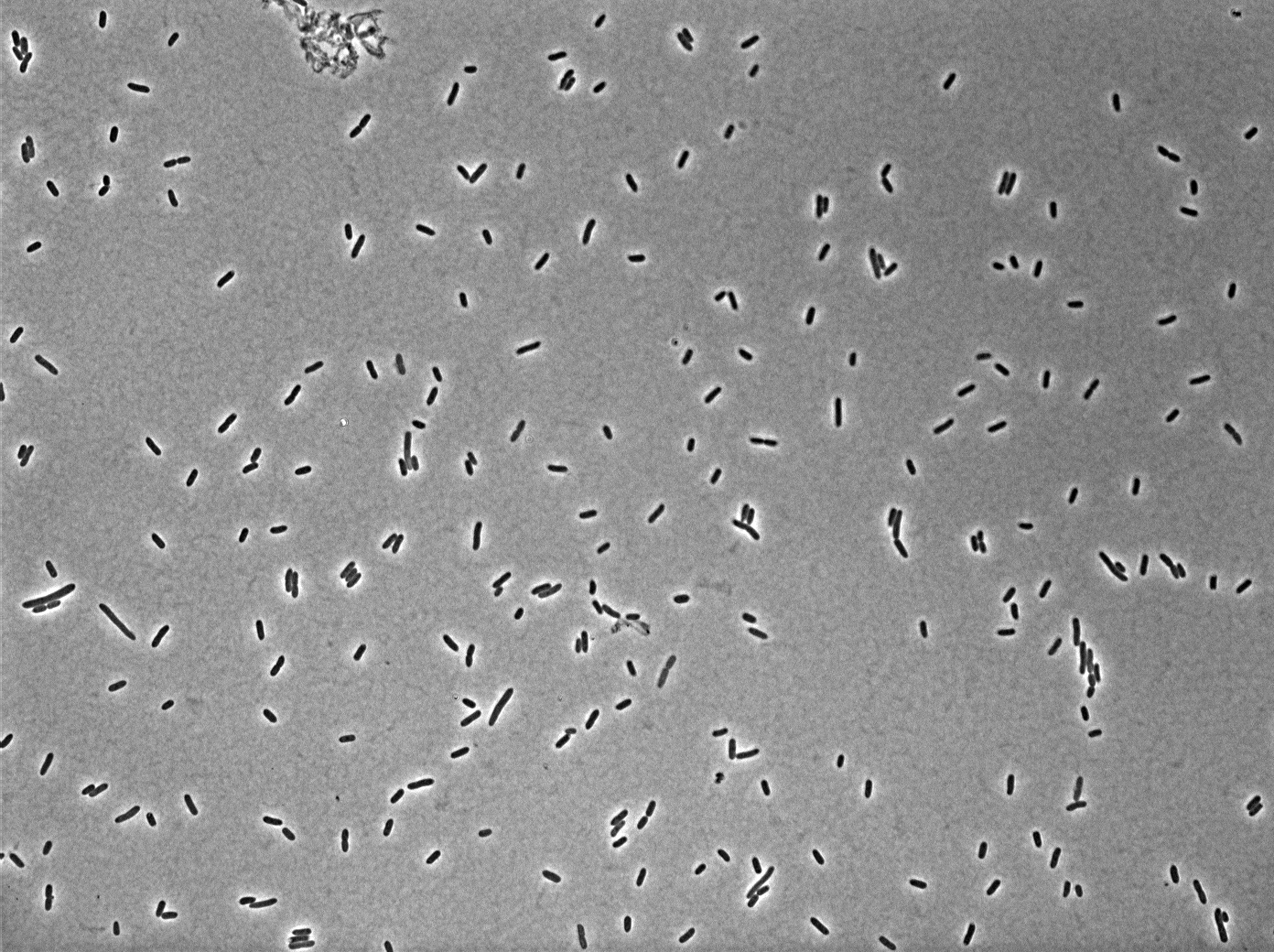

Supplement: Figure 4—source data 2. — The files can be opened in ImageJ. [file elife-82437-fig4-data2.zip › Figure 4- source data 2/TseV3 DAPI images/pBRA_TseV3_Gluc.lif - Image003.tif]

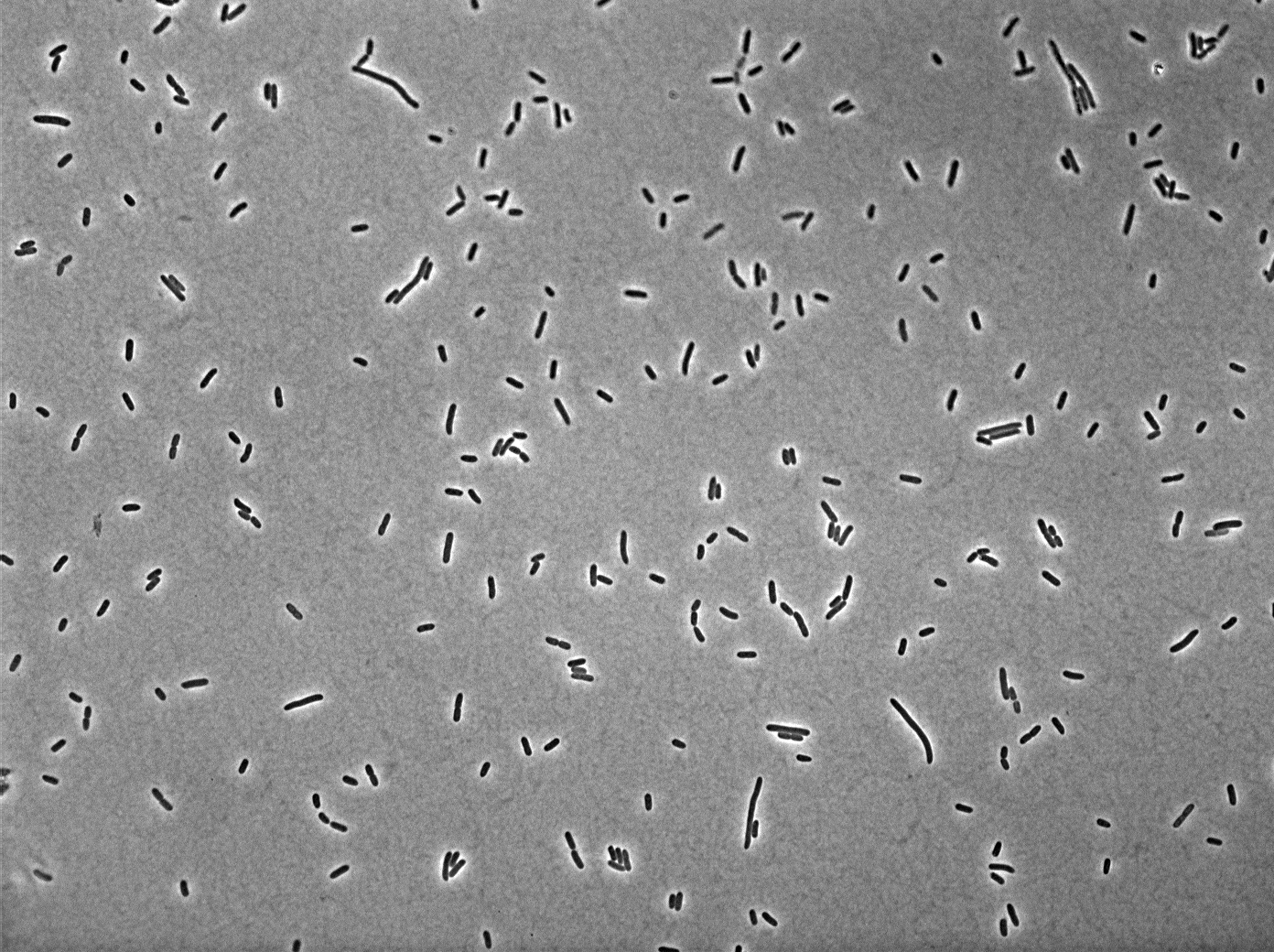

Supplement: Figure 4—source data 2. — The files can be opened in ImageJ. [file elife-82437-fig4-data2.zip › Figure 4- source data 2/TseV3 DAPI images/pBRA_TseV3_Gluc.lif - Image004.tif]

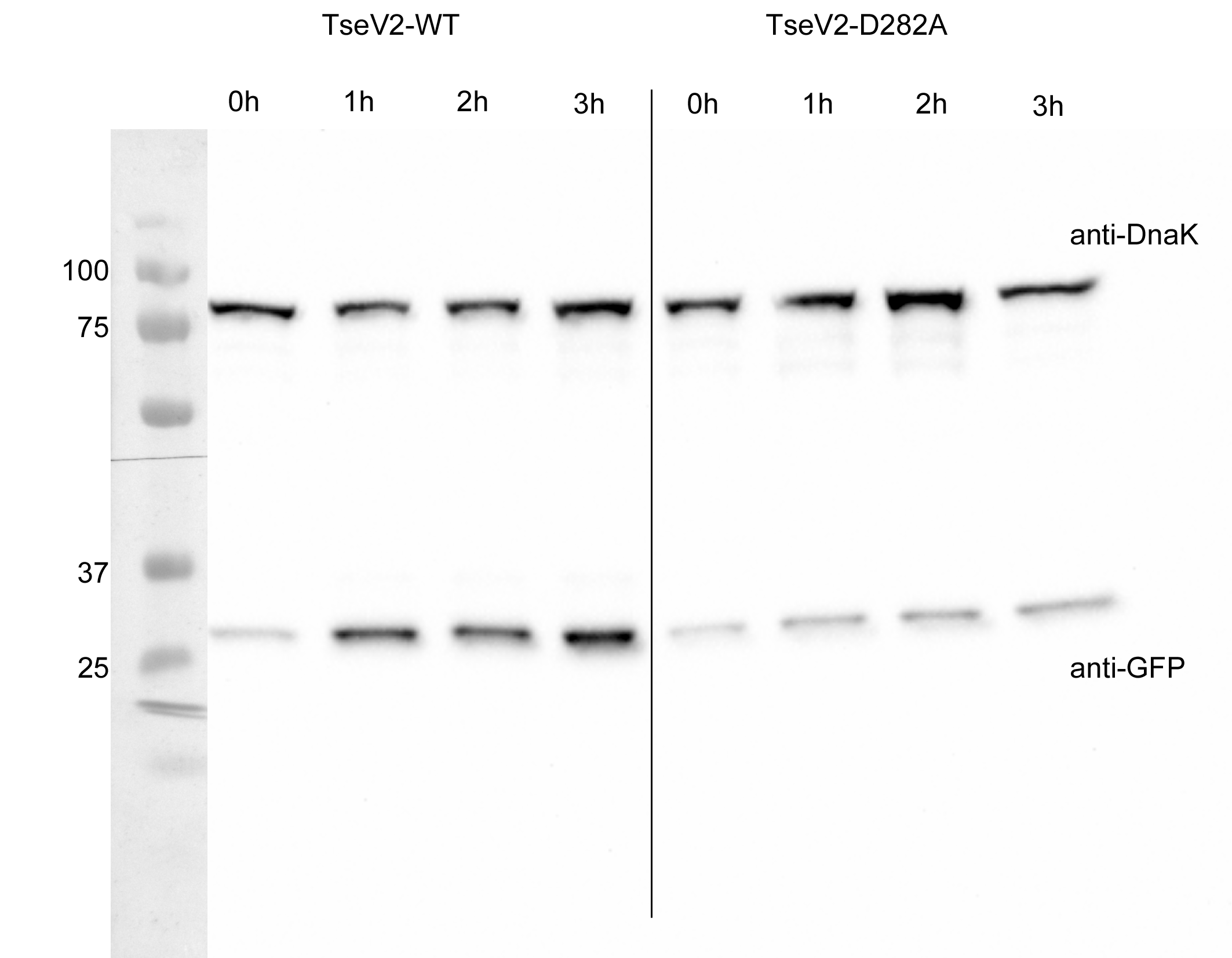

Supplement: Figure 4—figure supplement 1—source data 1. [file elife-82437-fig4-figsupp1-data1.zip › Figure 4- figure supplement 1- source data 1/TseV2 labels.tif]

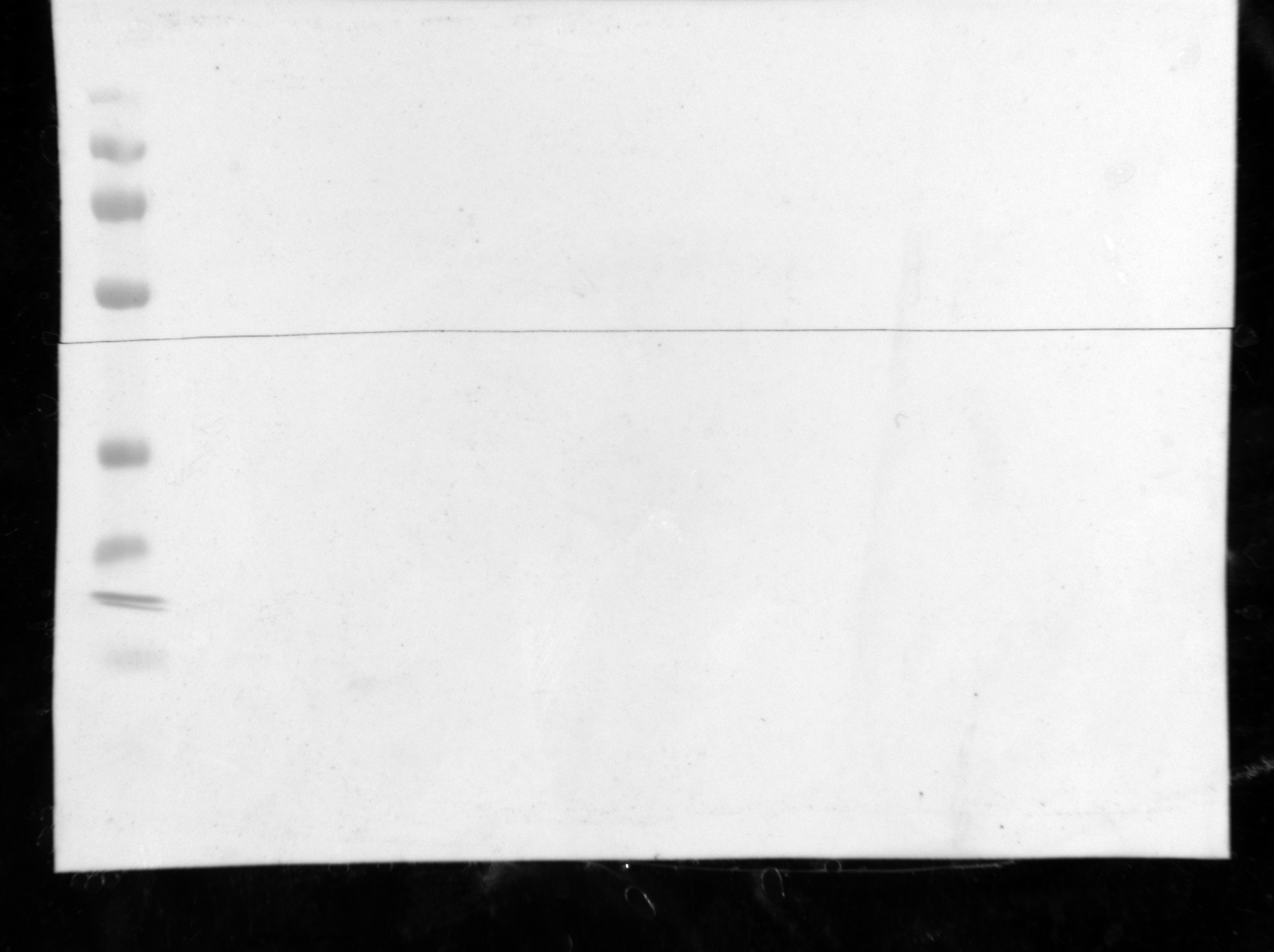

Supplement: Figure 4—figure supplement 1—source data 1. [file elife-82437-fig4-figsupp1-data1.zip › Figure 4- figure supplement 1- source data 1/TseV2 ladder.tif]

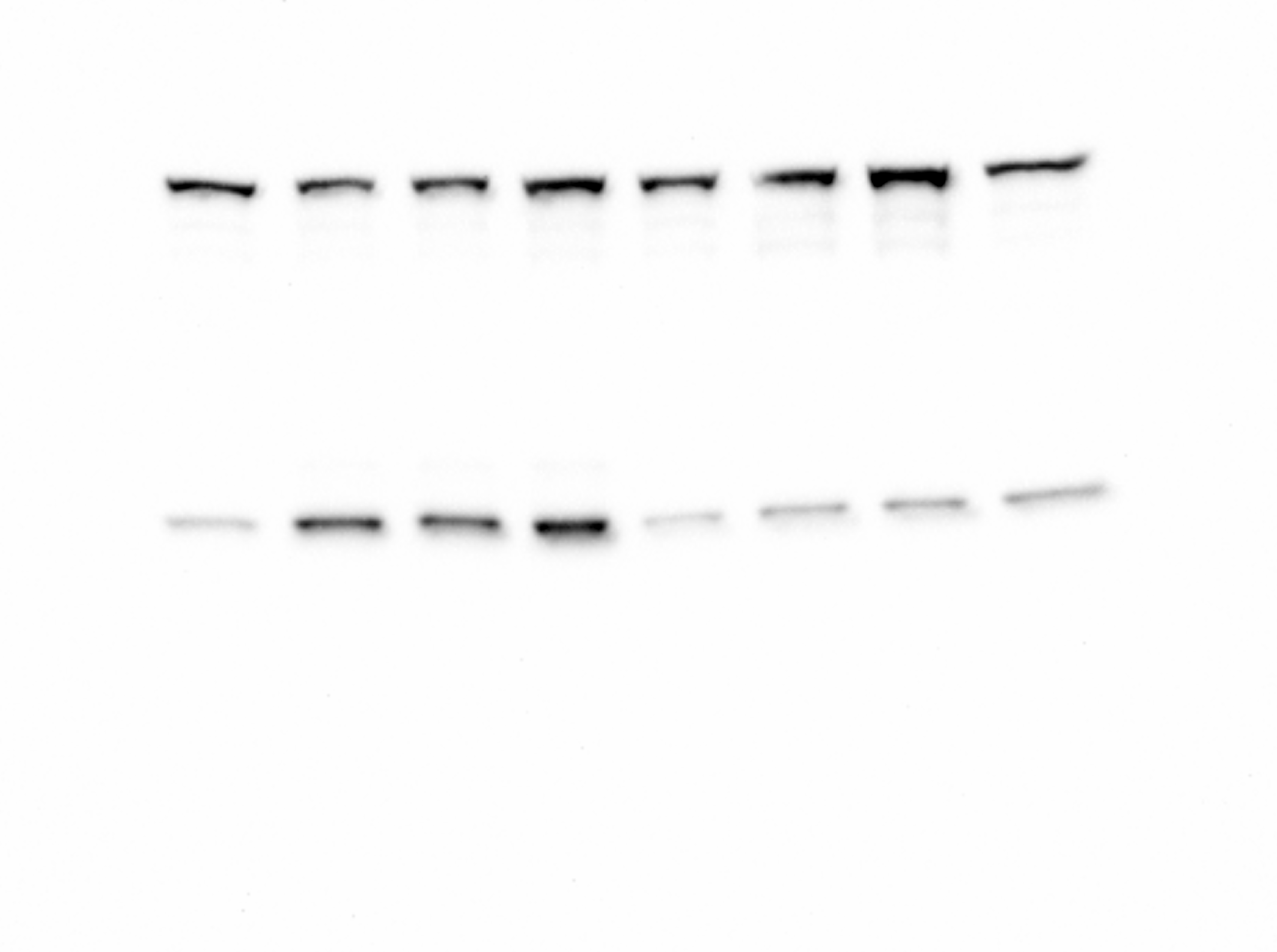

Supplement: Figure 4—figure supplement 1—source data 1. [file elife-82437-fig4-figsupp1-data1.zip › Figure 4- figure supplement 1- source data 1/TseV2 WB.tif]

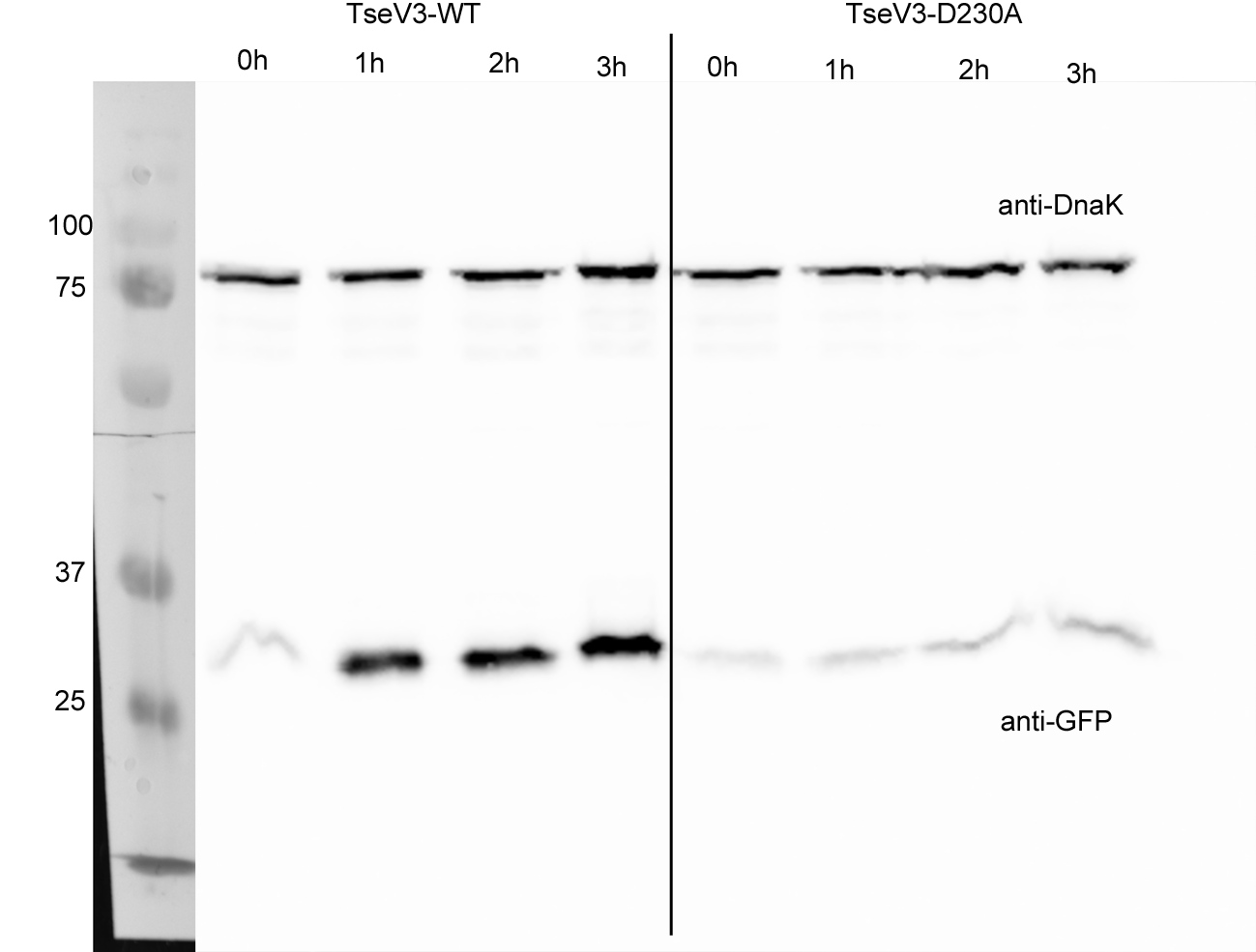

Supplement: Figure 4—figure supplement 1—source data 1. [file elife-82437-fig4-figsupp1-data1.zip › Figure 4- figure supplement 1- source data 1/TseV3 labels.tif]

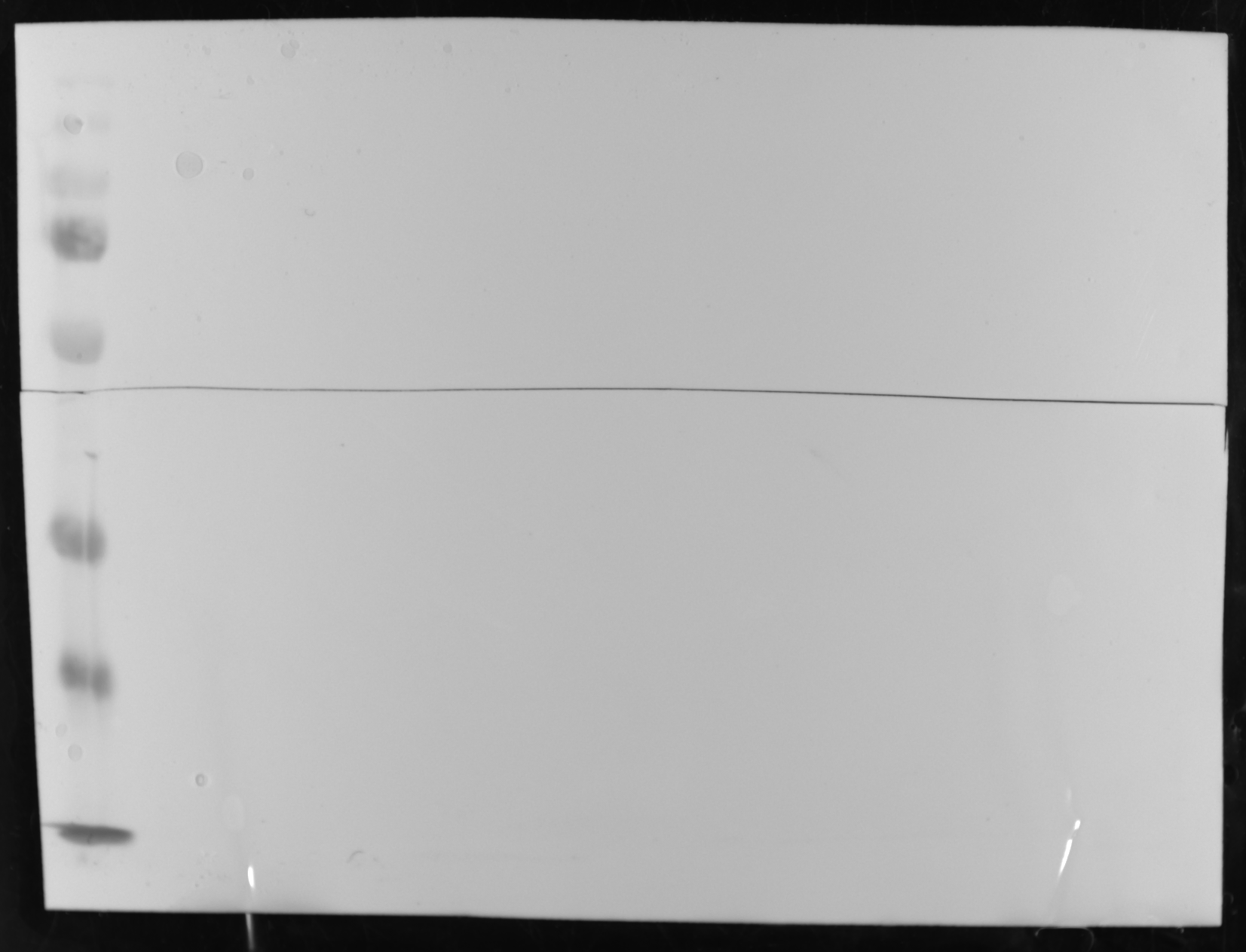

Supplement: Figure 4—figure supplement 1—source data 1. [file elife-82437-fig4-figsupp1-data1.zip › Figure 4- figure supplement 1- source data 1/TseV3 ladder.Tif]

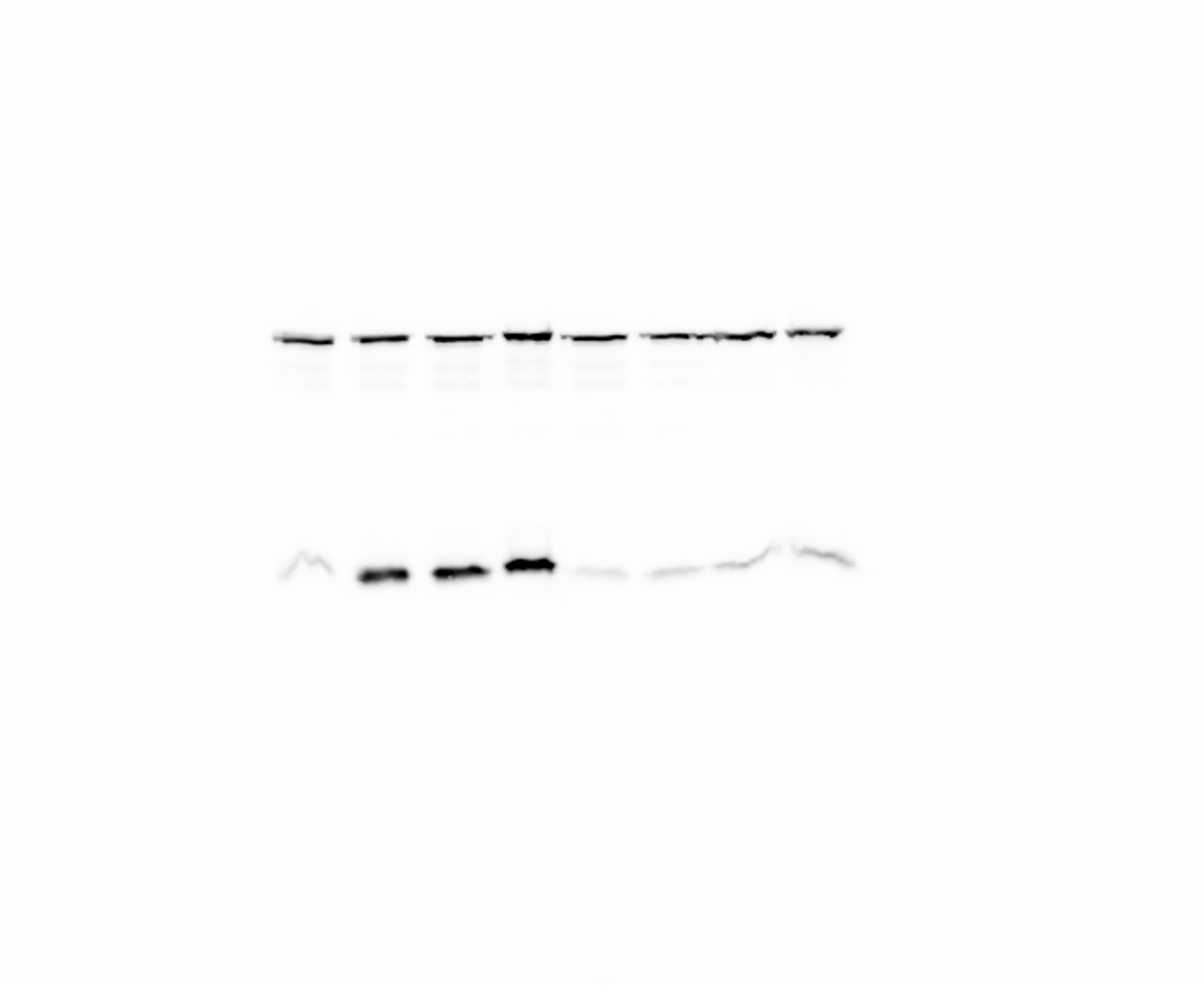

Supplement: Figure 4—figure supplement 1—source data 1. [file elife-82437-fig4-figsupp1-data1.zip › Figure 4- figure supplement 1- source data 1/TseV3 WB.Tif]

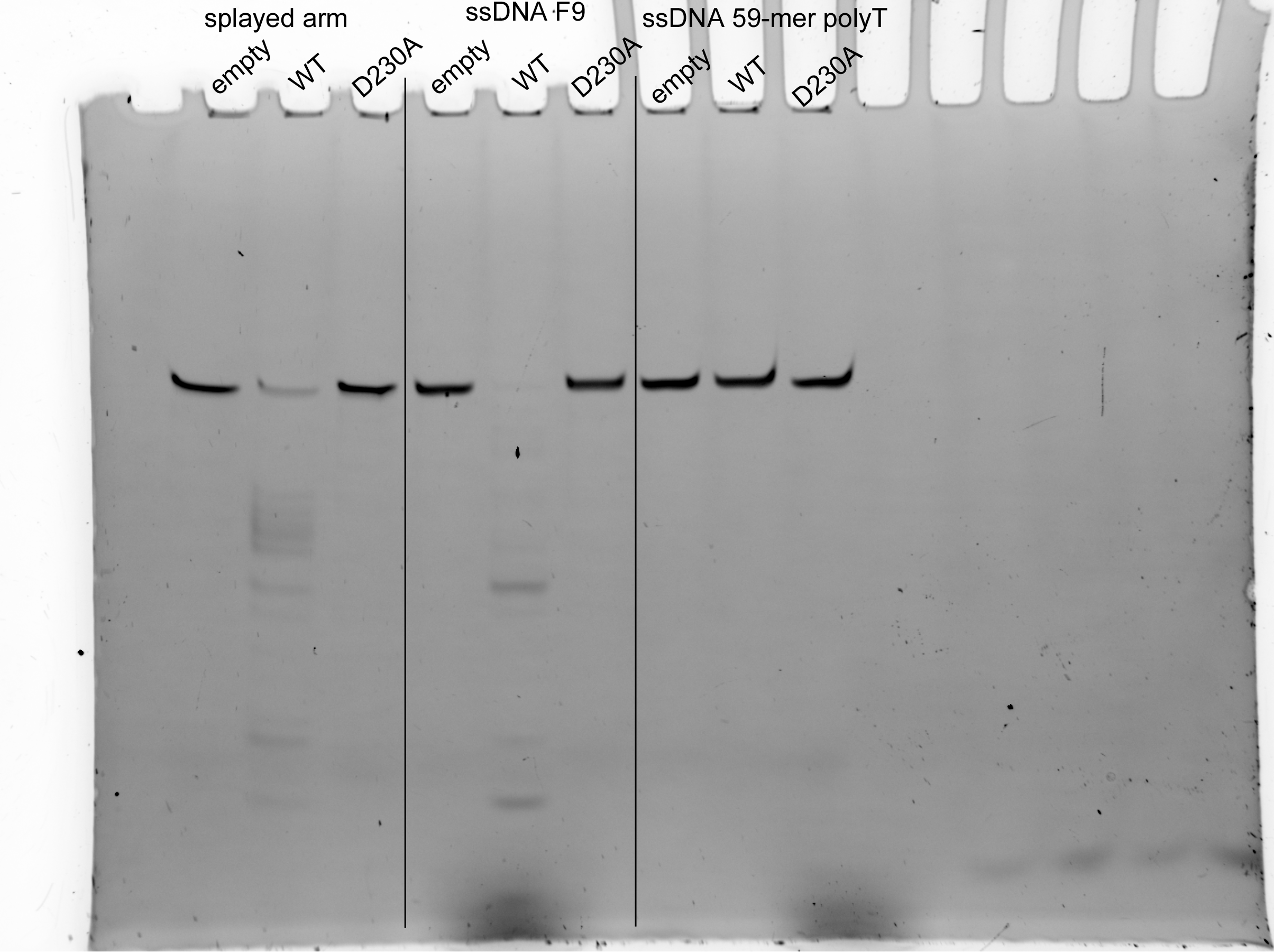

Supplement: Figure 5—source data 1. [file elife-82437-fig5-data1.zip › Figure 5- source data 1/ssDNA degradation by TseV3 labels.tif]

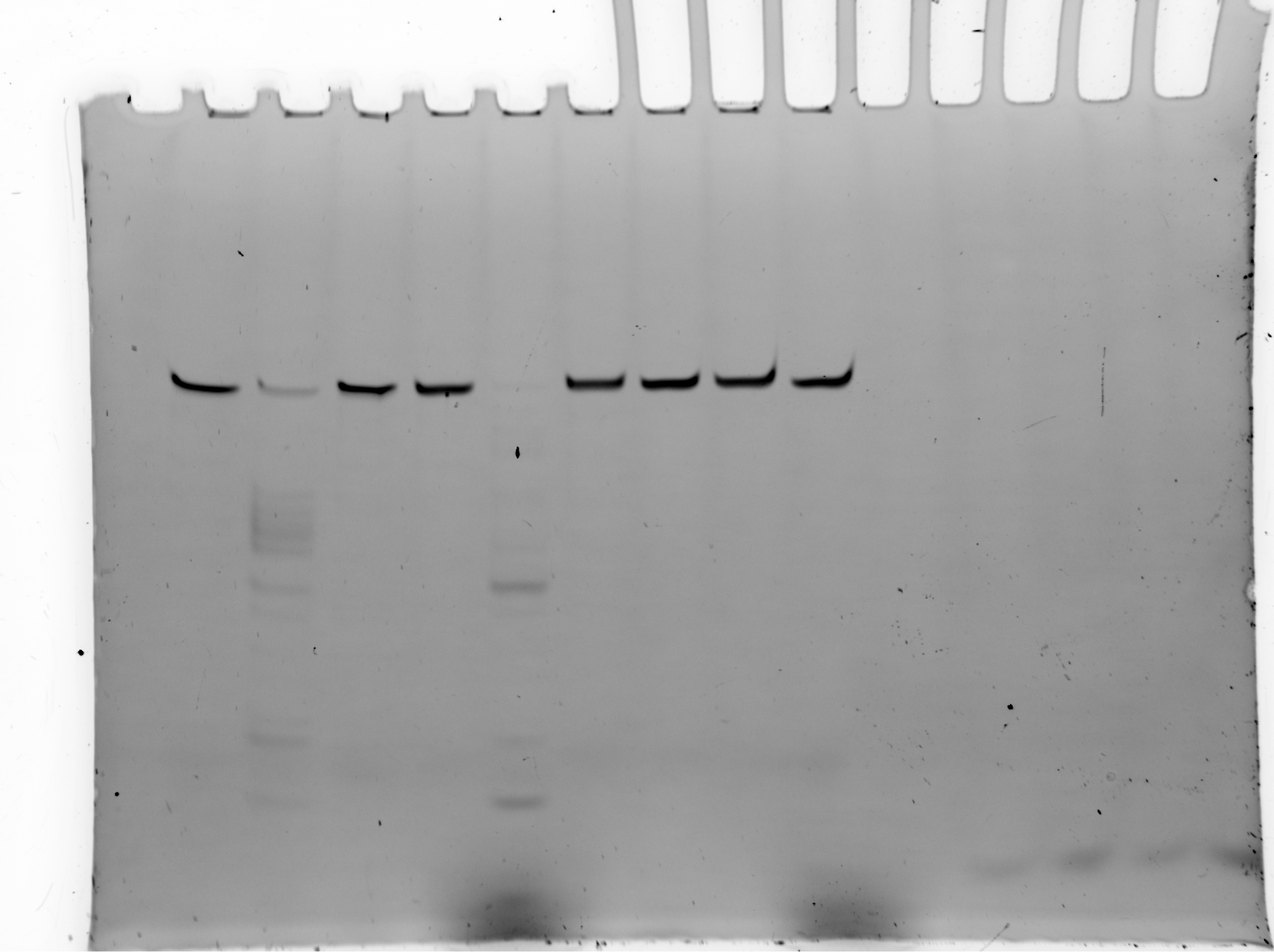

Supplement: Figure 5—source data 1. [file elife-82437-fig5-data1.zip › Figure 5- source data 1/ssDNA degradation by TseV3.tif]

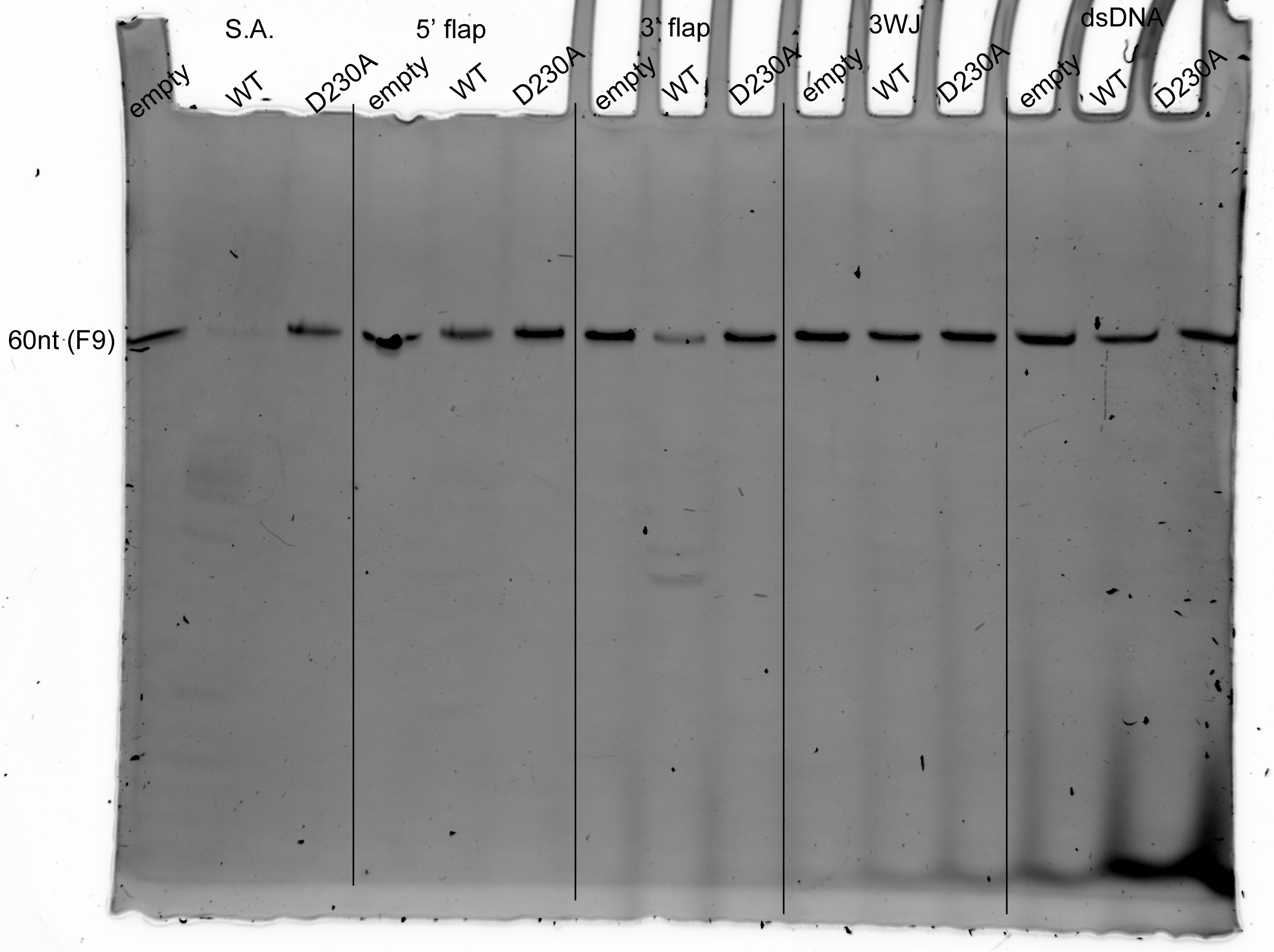

Supplement: Figure 5—source data 1. [file elife-82437-fig5-data1.zip › Figure 5- source data 1/Substrates degradation by TseV3 labels.tif]

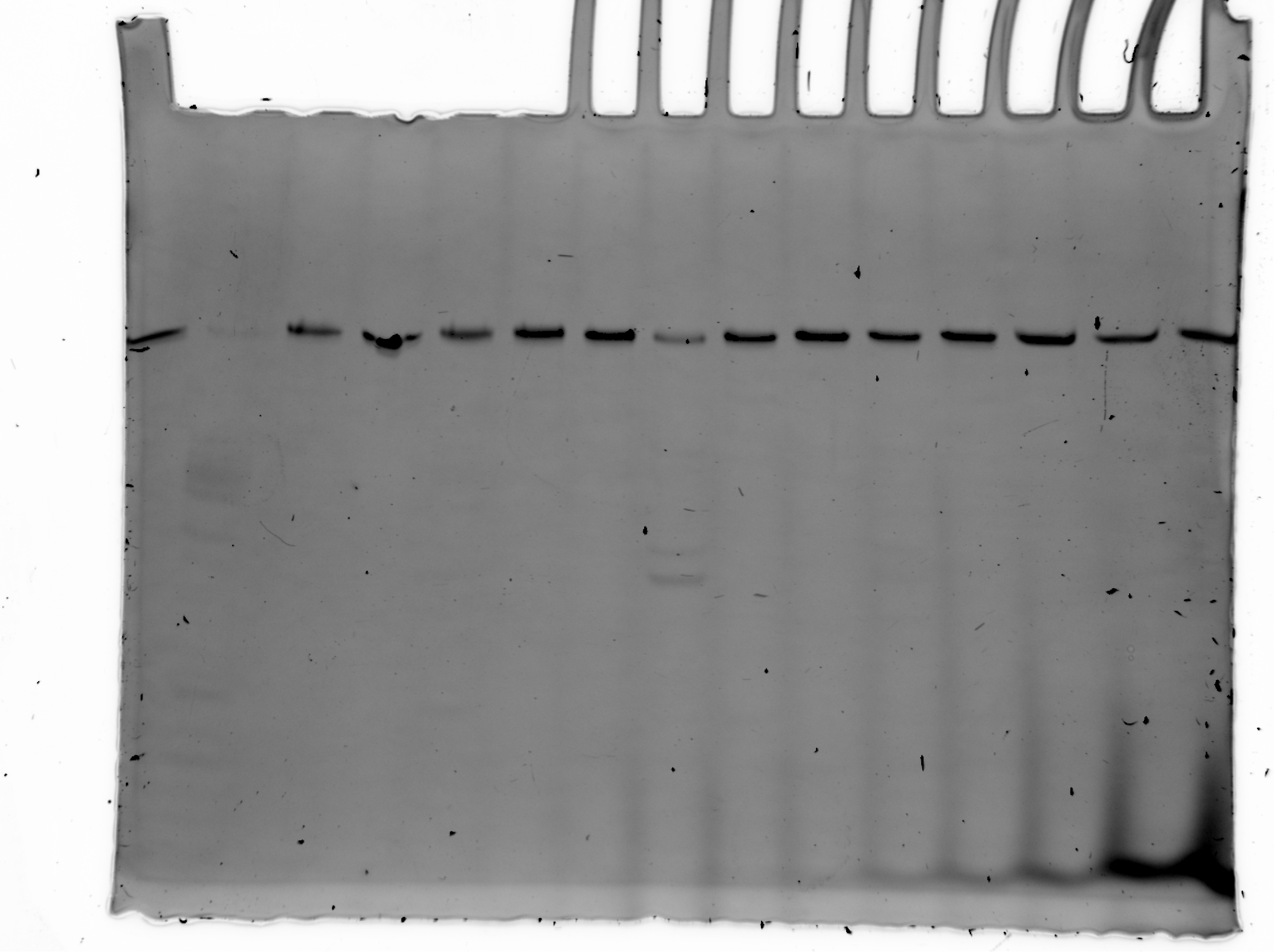

Supplement: Figure 5—source data 1. [file elife-82437-fig5-data1.zip › Figure 5- source data 1/Substrates degradation by TseV3.tif]

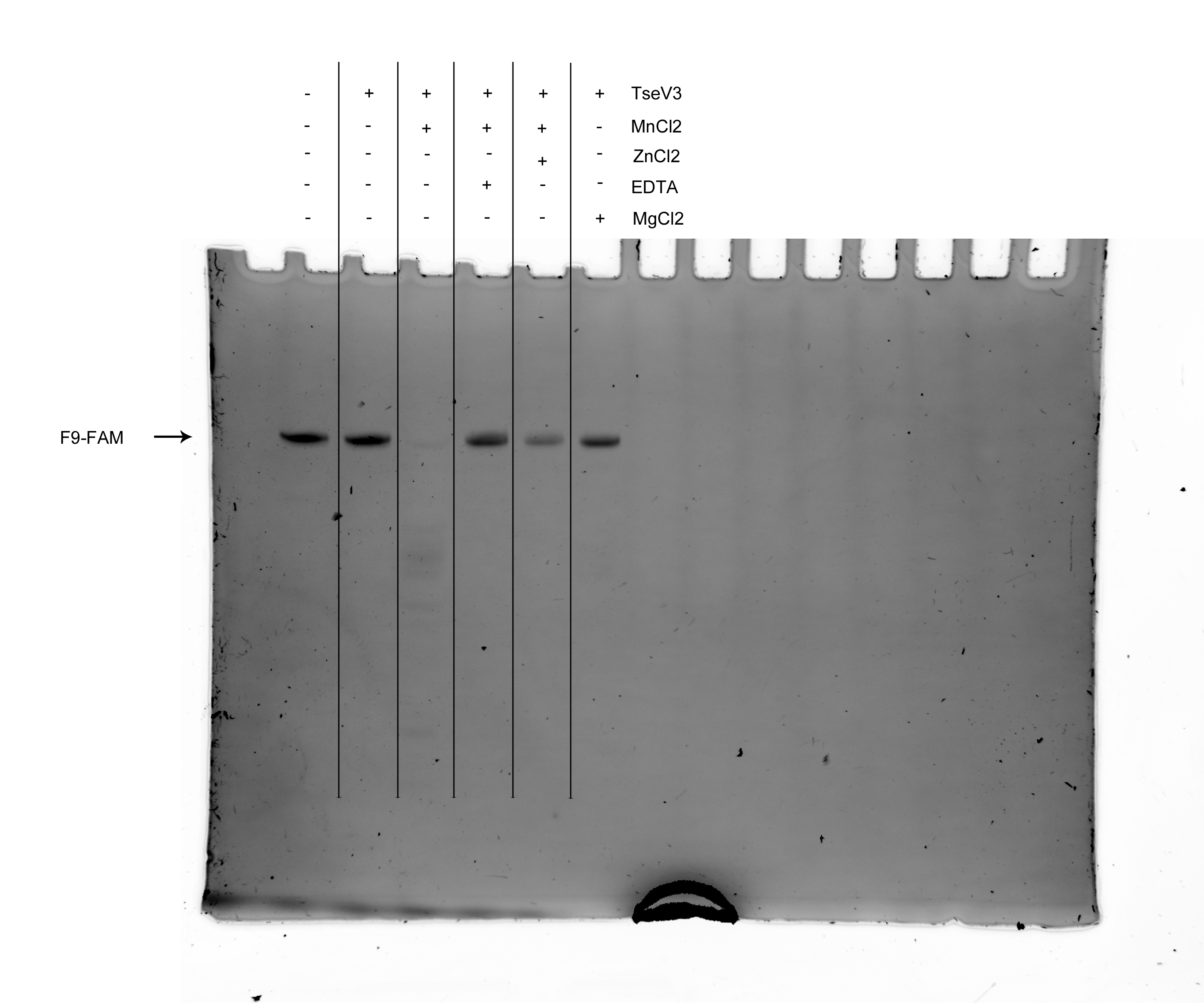

Supplement: Figure 5—source data 2. [file elife-82437-fig5-data2.zip › Figure 5- source data 2/Co-factors test with TseV3 labels.tif]

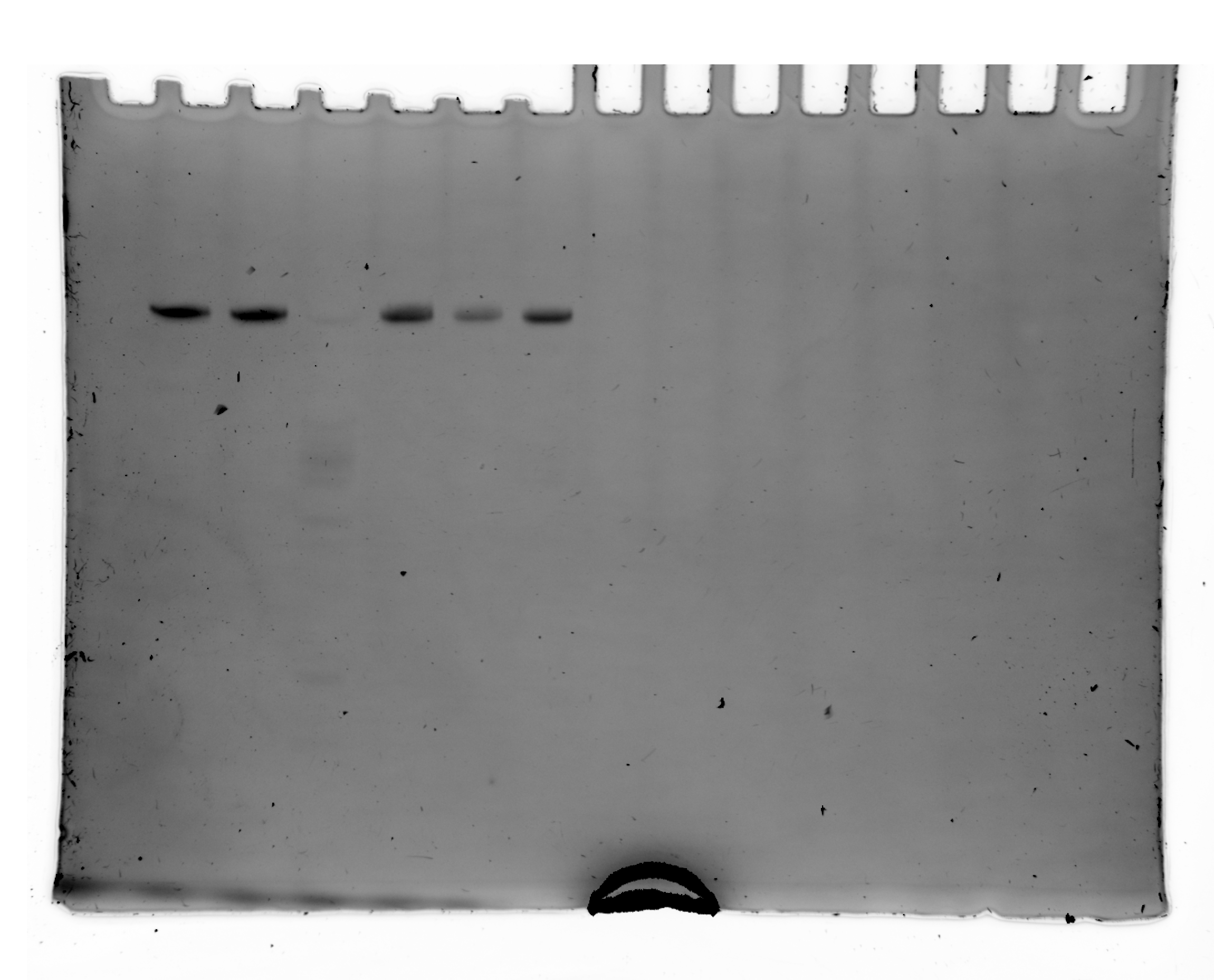

Supplement: Figure 5—source data 2. [file elife-82437-fig5-data2.zip › Figure 5- source data 2/Co-factors test with TseV3 original.tif]

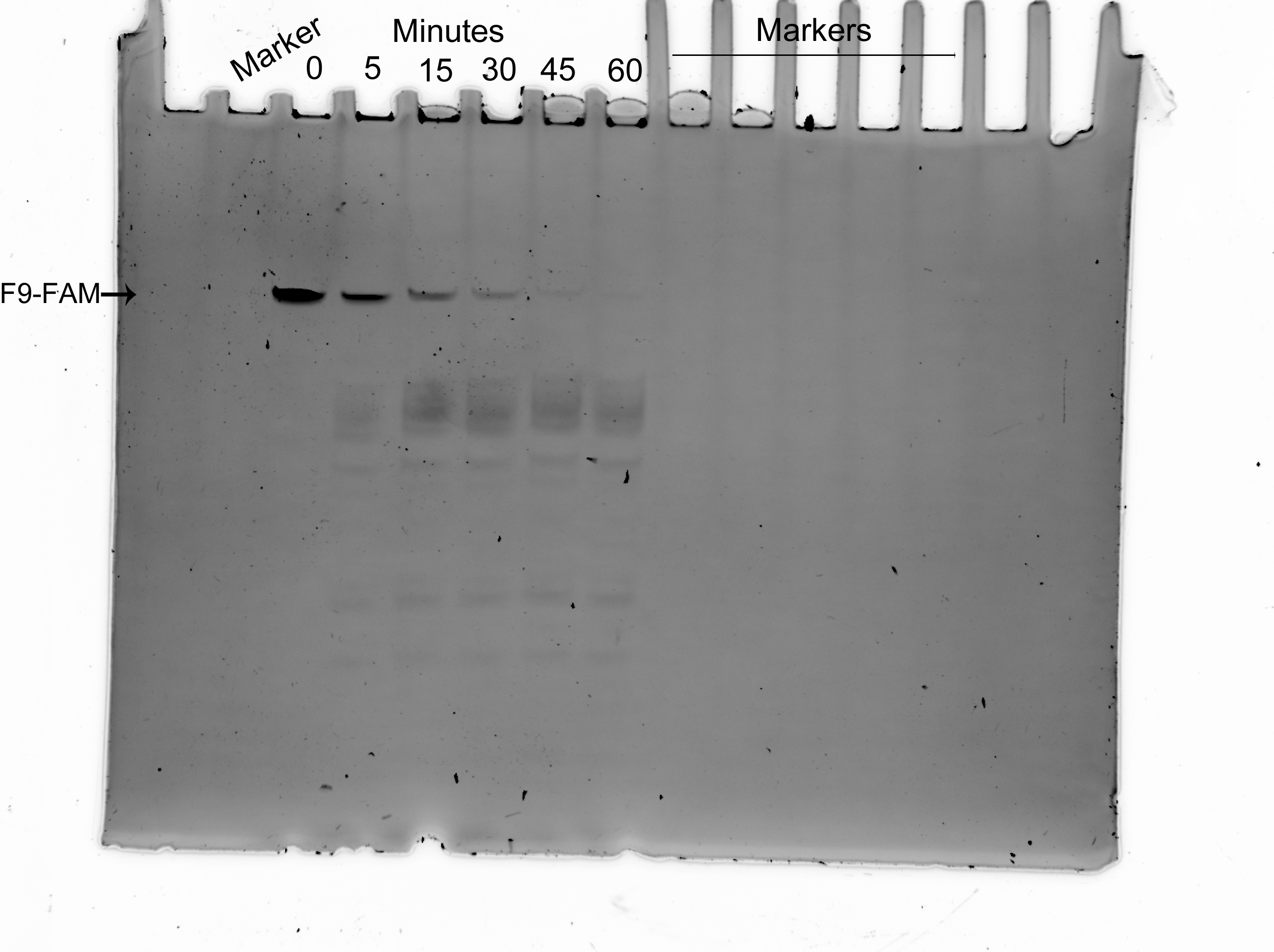

Supplement: Figure 5—source data 3. [file elife-82437-fig5-data3.zip › Figure 5- source data 3/Time course degradation with TseV3 labels.tif]

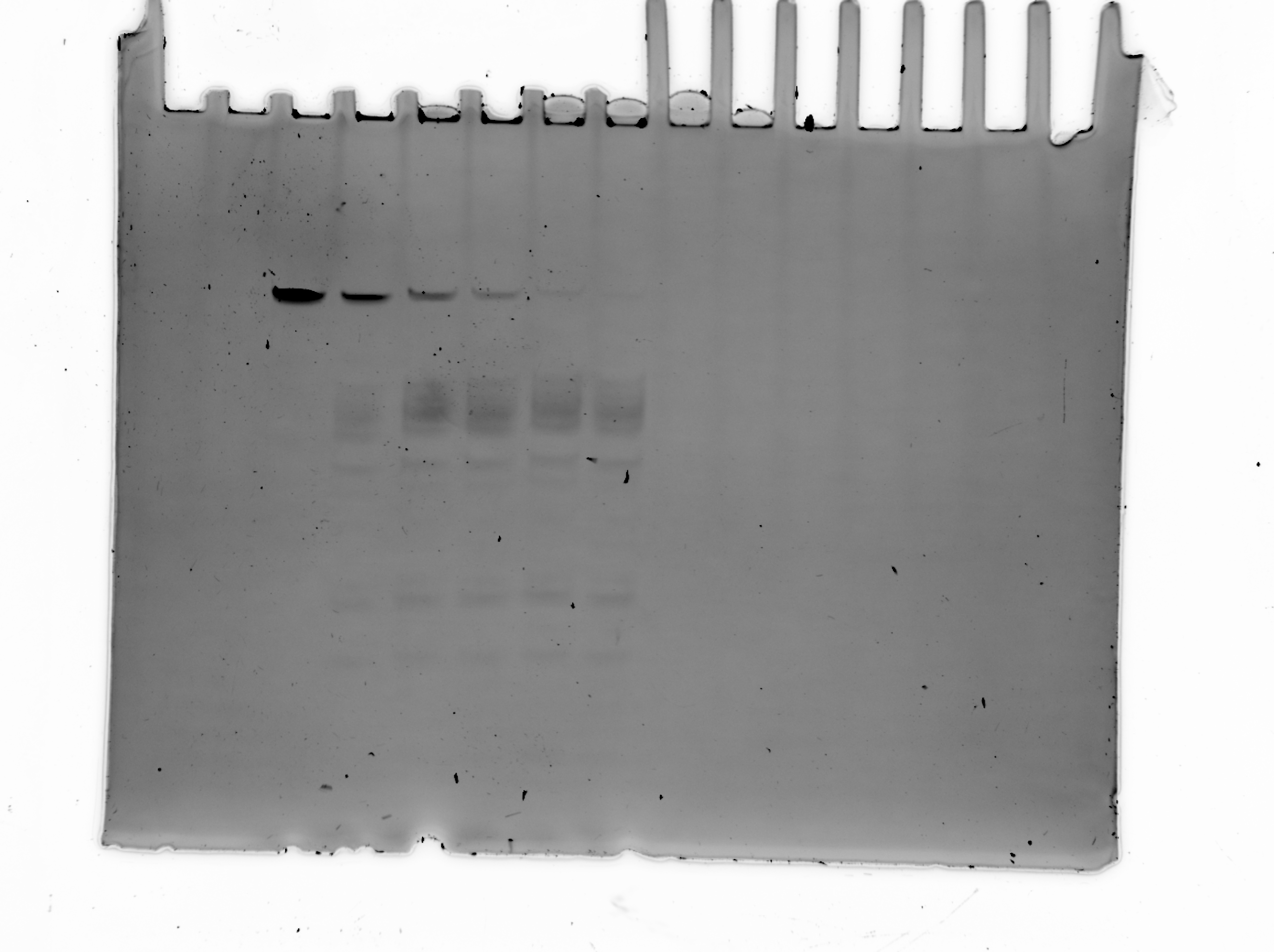

Supplement: Figure 5—source data 3. [file elife-82437-fig5-data3.zip › Figure 5- source data 3/Time course degradation with TseV3 original.tif]

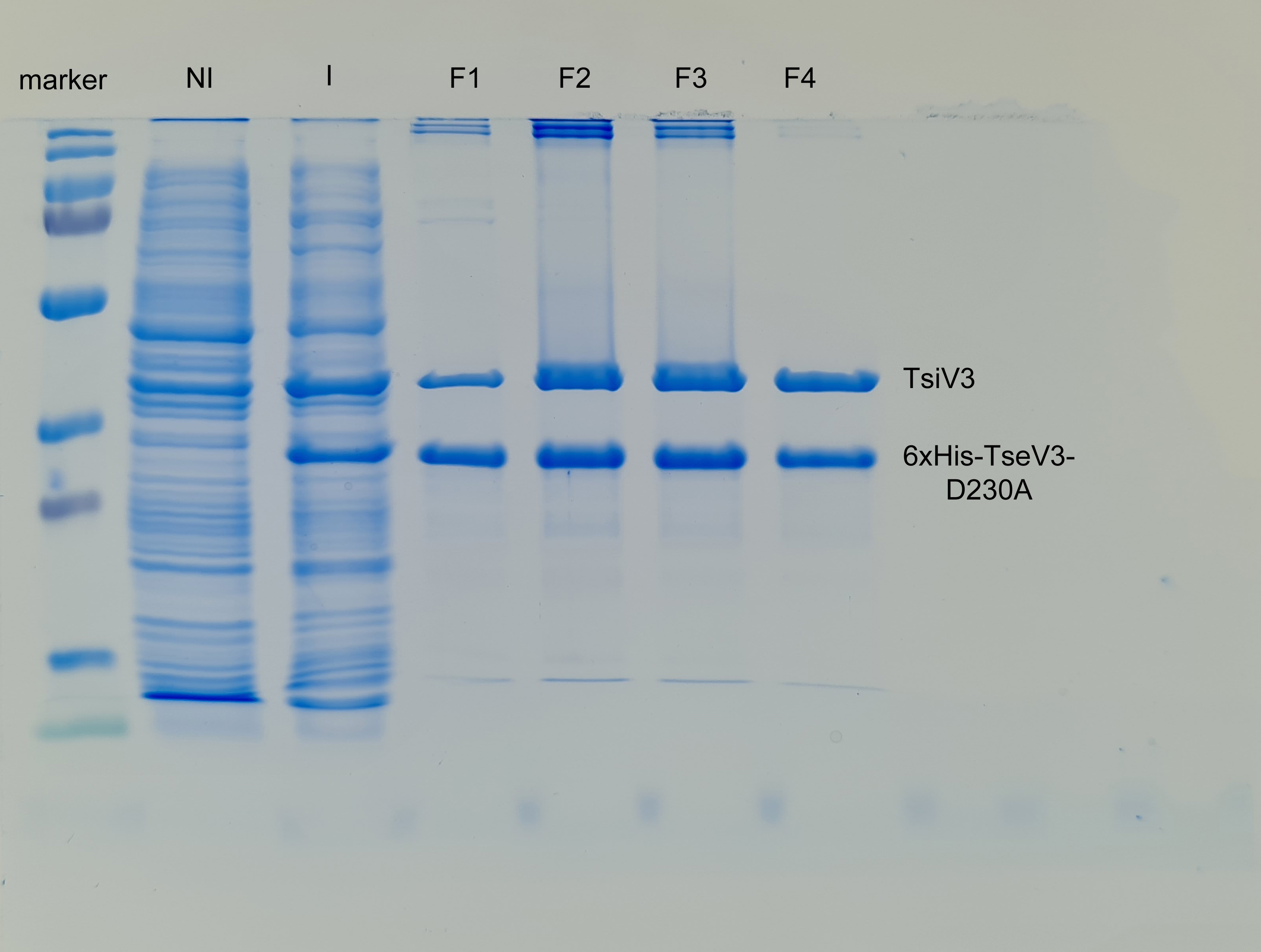

Supplement: Figure 5—figure supplement 1—source data 1. [file elife-82437-fig5-figsupp1-data1.zip › Figure 5- supplement 1 -source data 1/D230A/TseV3-D230A 1st affinity labels.jpg]

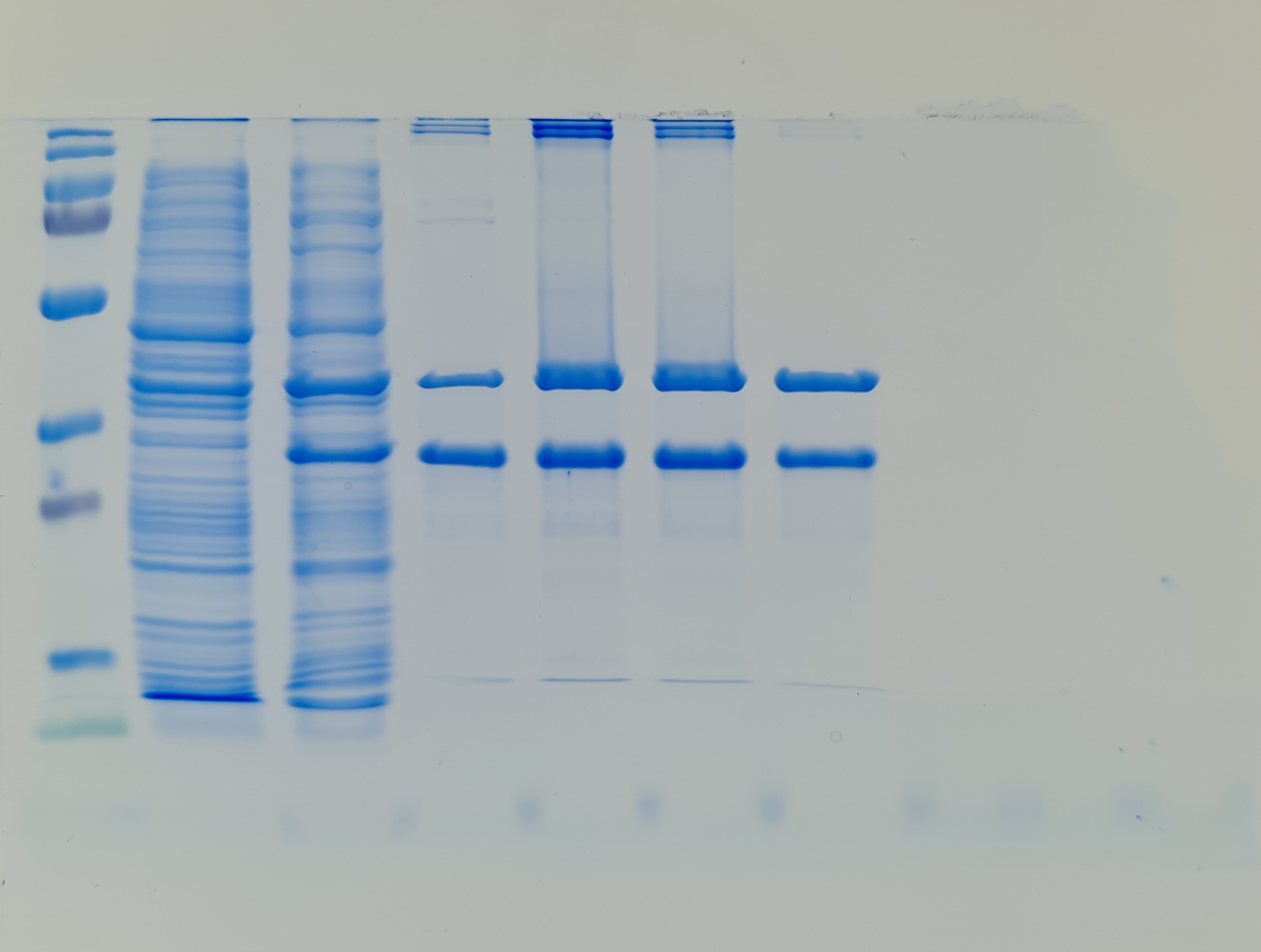

Supplement: Figure 5—figure supplement 1—source data 1. [file elife-82437-fig5-figsupp1-data1.zip › Figure 5- supplement 1 -source data 1/D230A/TseV3-D230A 1st affinity.tif]

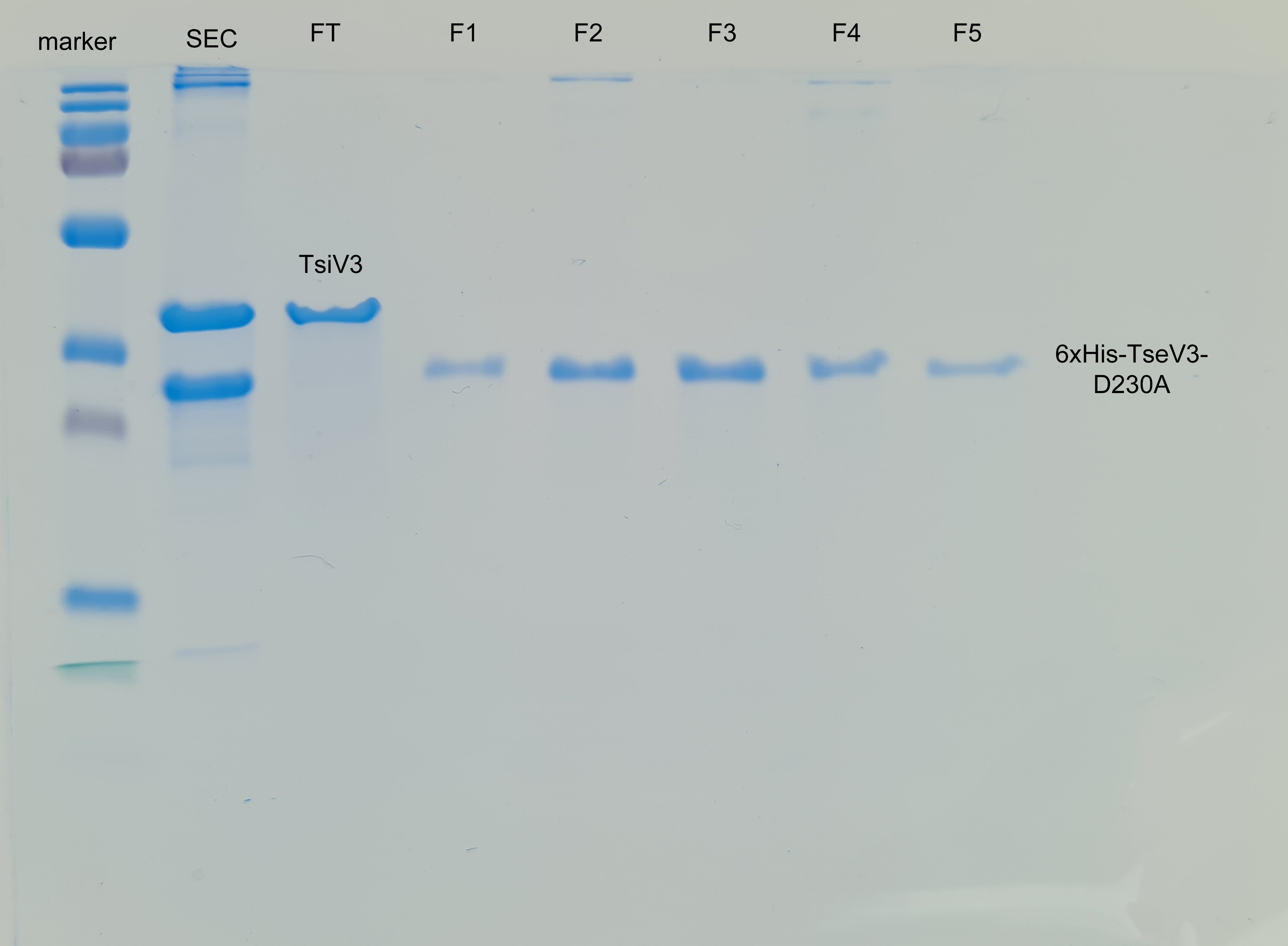

Supplement: Figure 5—figure supplement 1—source data 1. [file elife-82437-fig5-figsupp1-data1.zip › Figure 5- supplement 1 -source data 1/D230A/TseV3-D230A 2nd affinity pos urea denaturation labels.jpg]

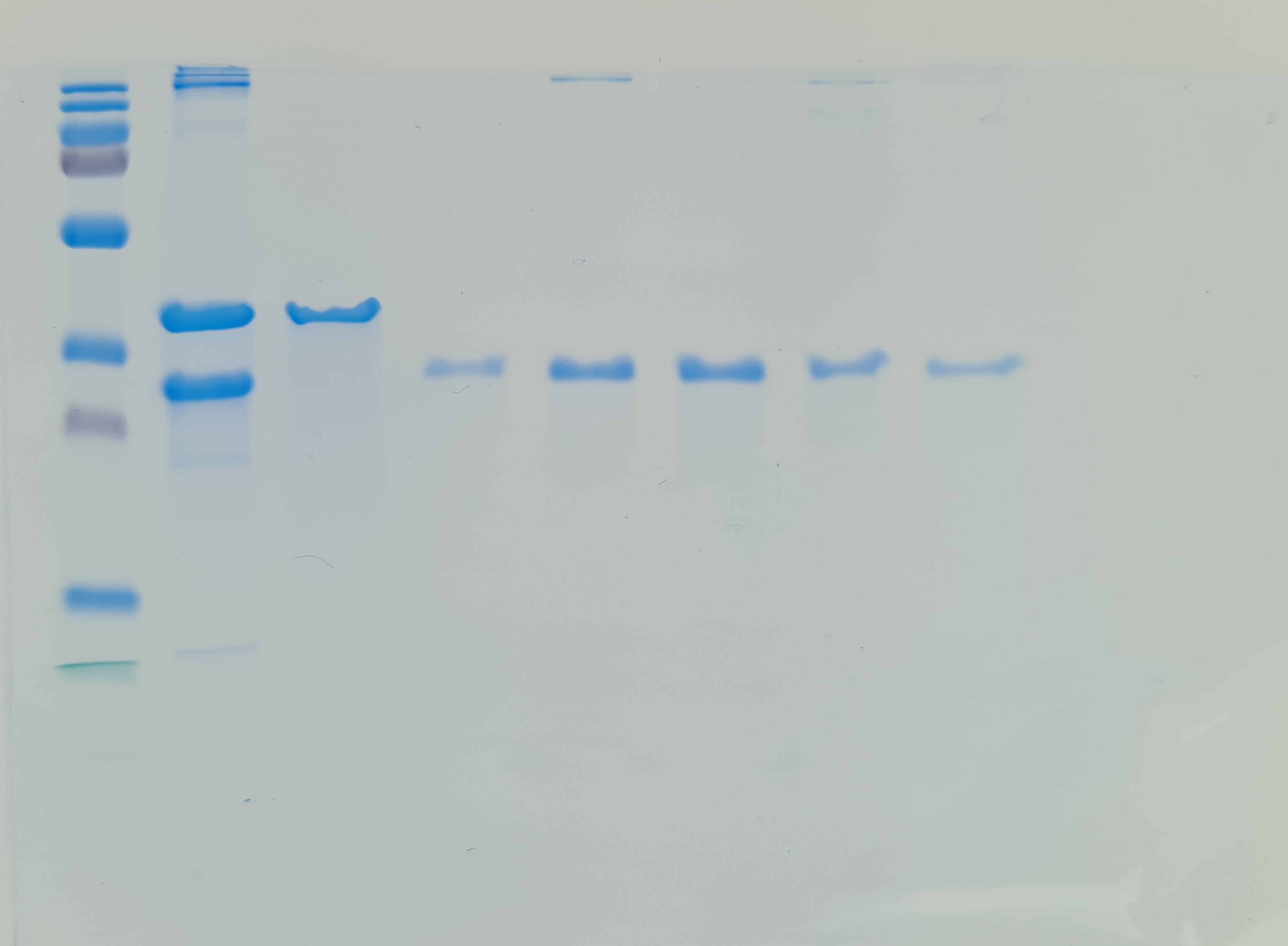

Supplement: Figure 5—figure supplement 1—source data 1. [file elife-82437-fig5-figsupp1-data1.zip › Figure 5- supplement 1 -source data 1/D230A/TseV3-D230A 2nd affinity pos urea denaturation.tif]

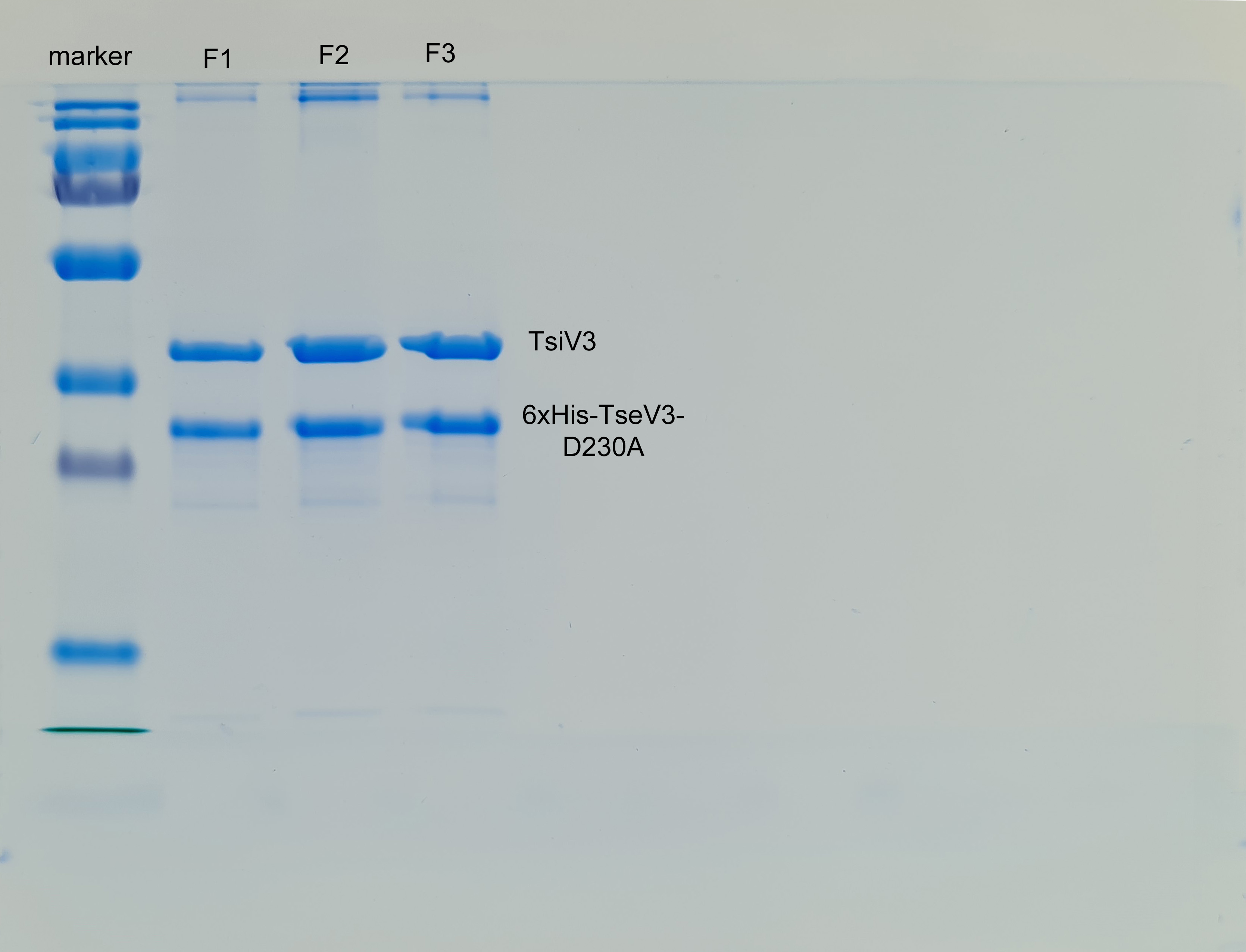

Supplement: Figure 5—figure supplement 1—source data 1. [file elife-82437-fig5-figsupp1-data1.zip › Figure 5- supplement 1 -source data 1/D230A/TseV3-D230A SEC labels.jpg]

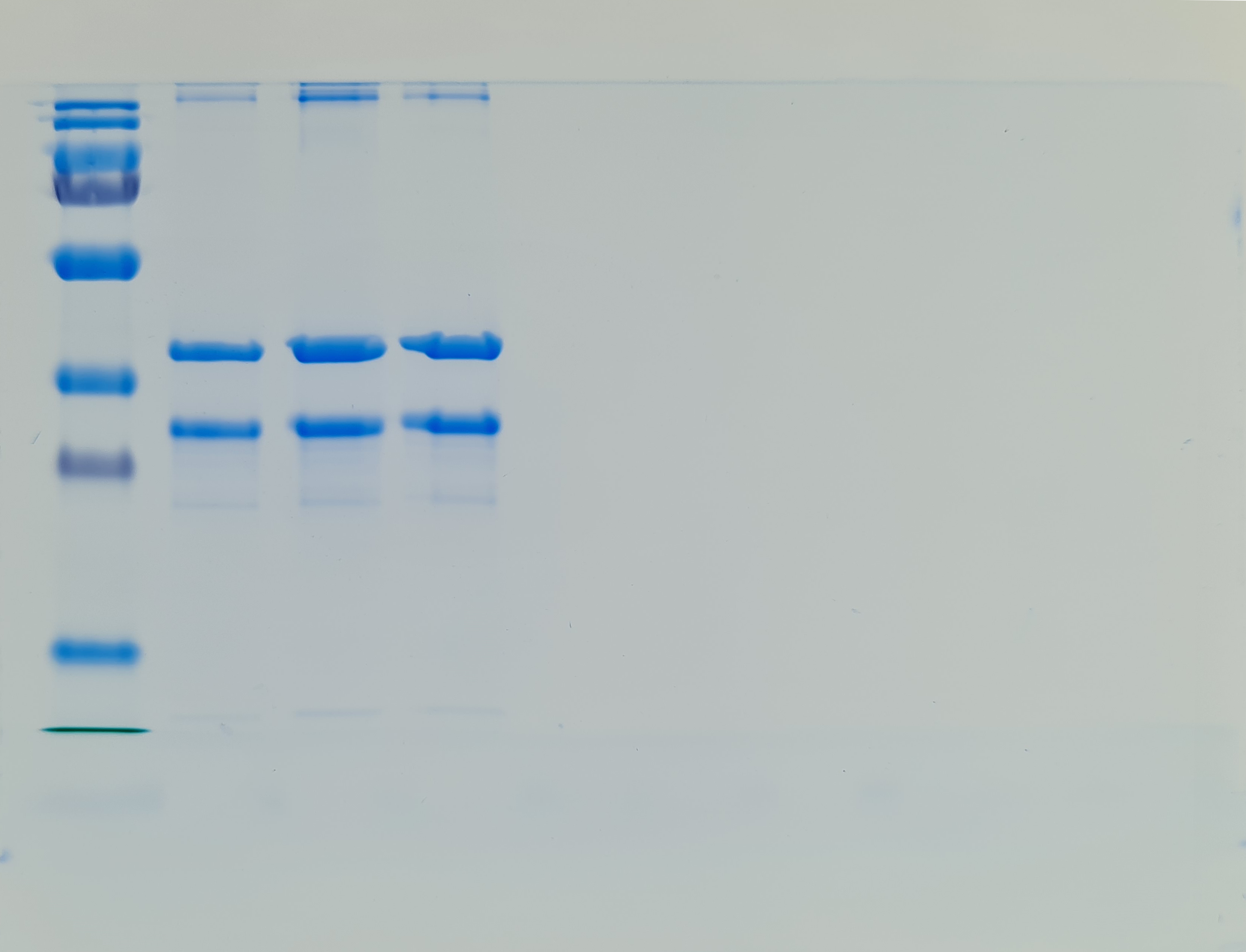

Supplement: Figure 5—figure supplement 1—source data 1. [file elife-82437-fig5-figsupp1-data1.zip › Figure 5- supplement 1 -source data 1/D230A/TseV3-D230A SEC.tif]

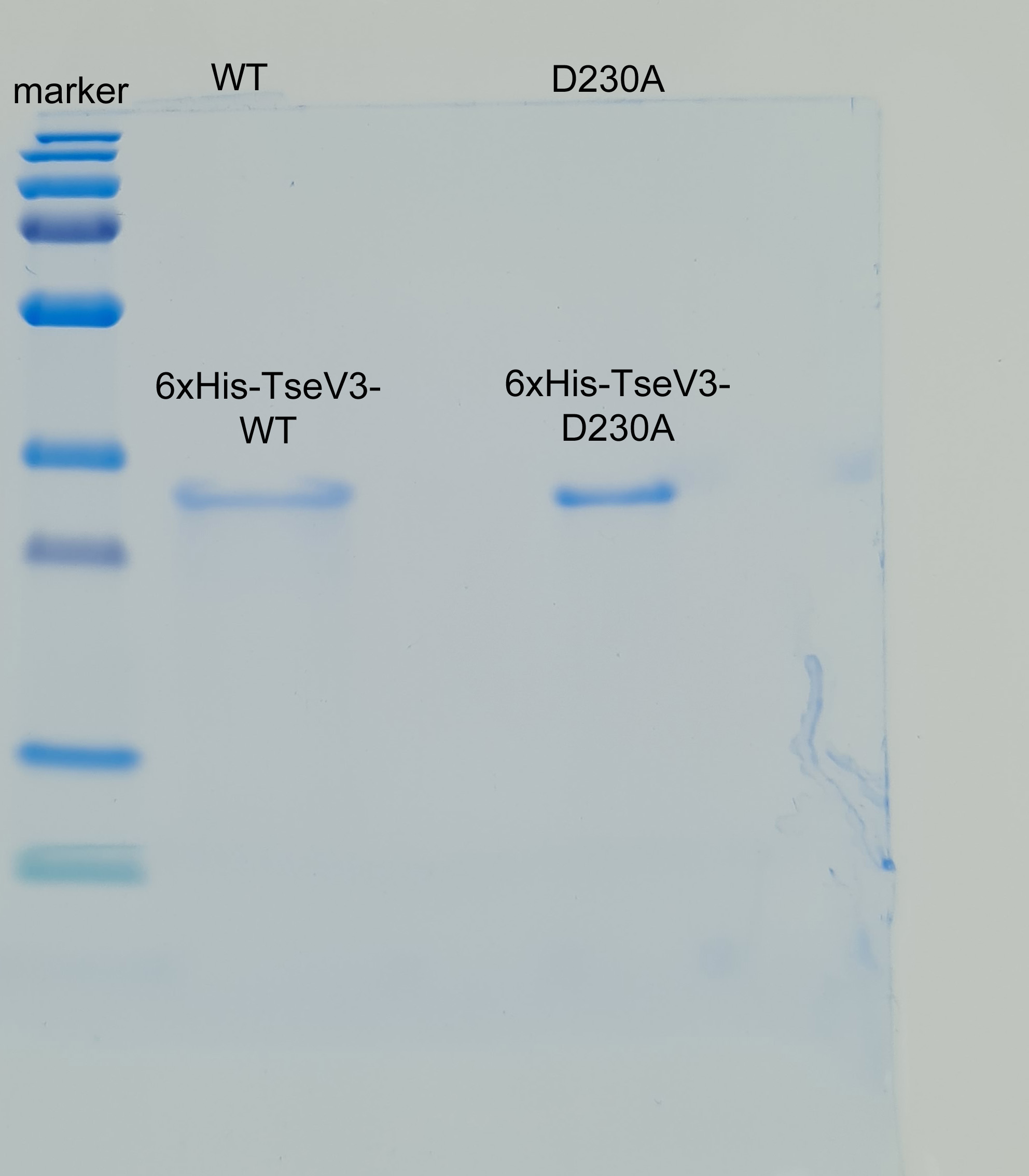

Supplement: Figure 5—figure supplement 1—source data 1. [file elife-82437-fig5-figsupp1-data1.zip › Figure 5- supplement 1 -source data 1/WT/TseV3 WT_D230A refolded labels cópia.jpg]

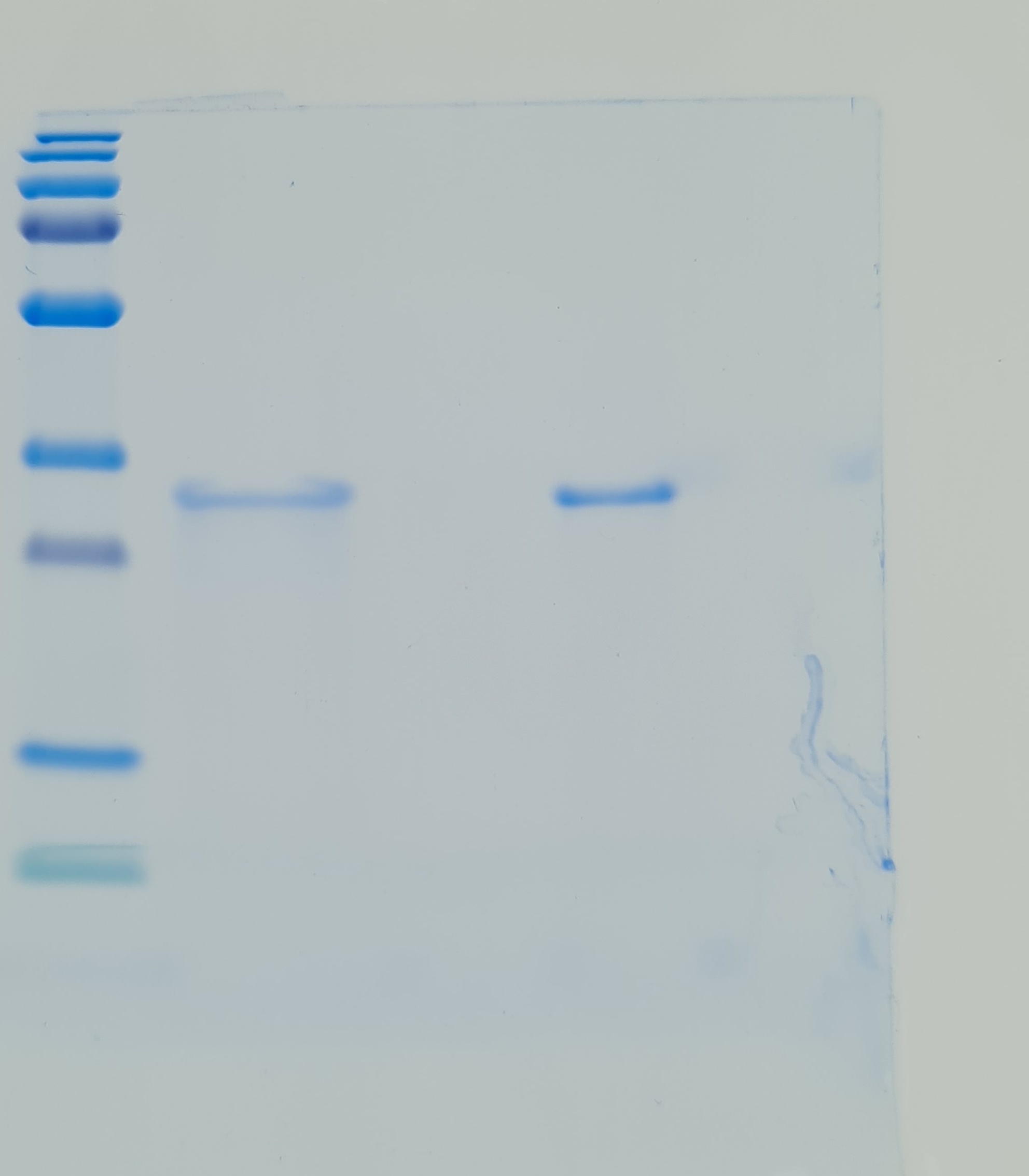

Supplement: Figure 5—figure supplement 1—source data 1. [file elife-82437-fig5-figsupp1-data1.zip › Figure 5- supplement 1 -source data 1/WT/TseV3 WT_D230A refolded.tif]

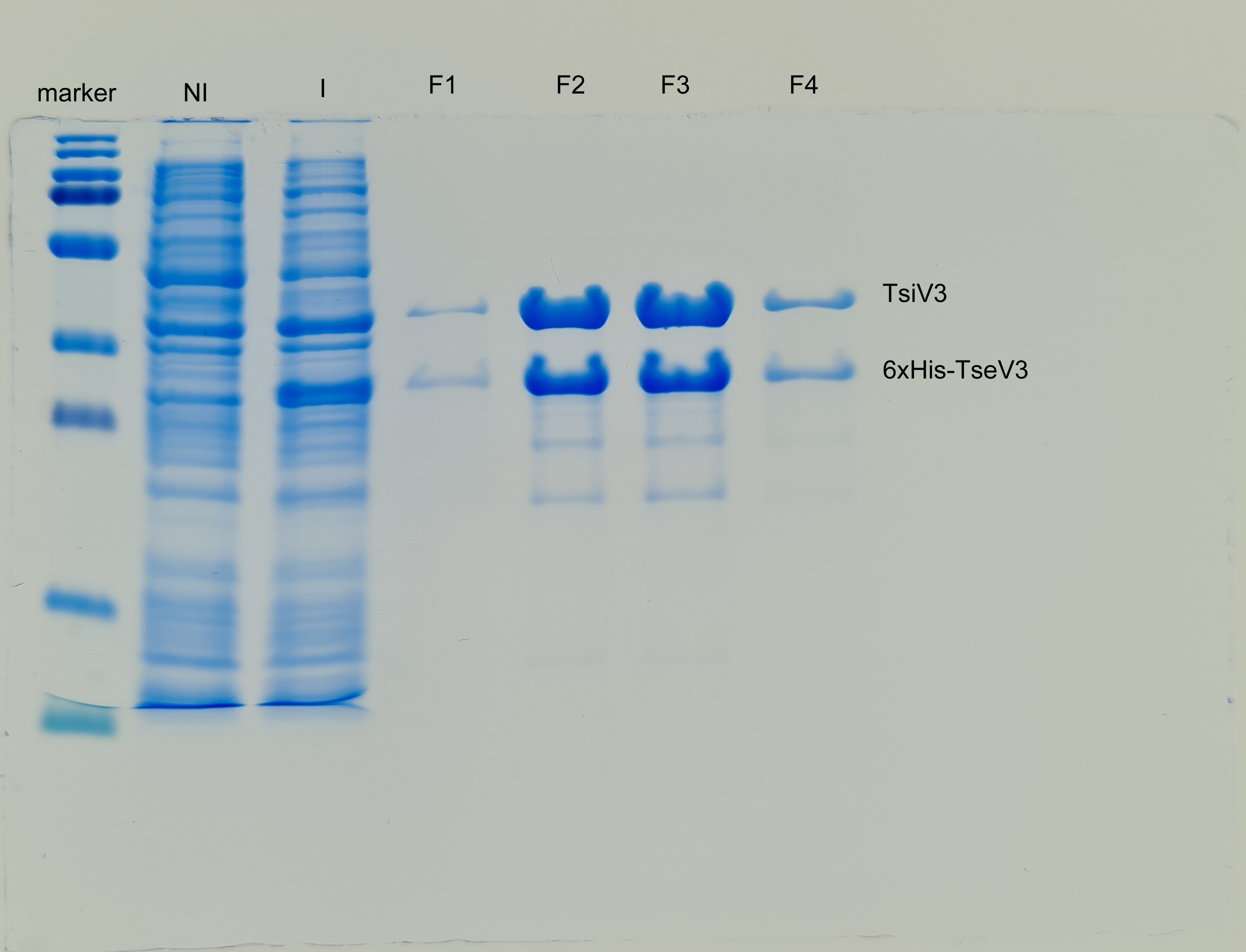

Supplement: Figure 5—figure supplement 1—source data 1. [file elife-82437-fig5-figsupp1-data1.zip › Figure 5- supplement 1 -source data 1/WT/TseV3-WT 1st affinity labels.jpg]

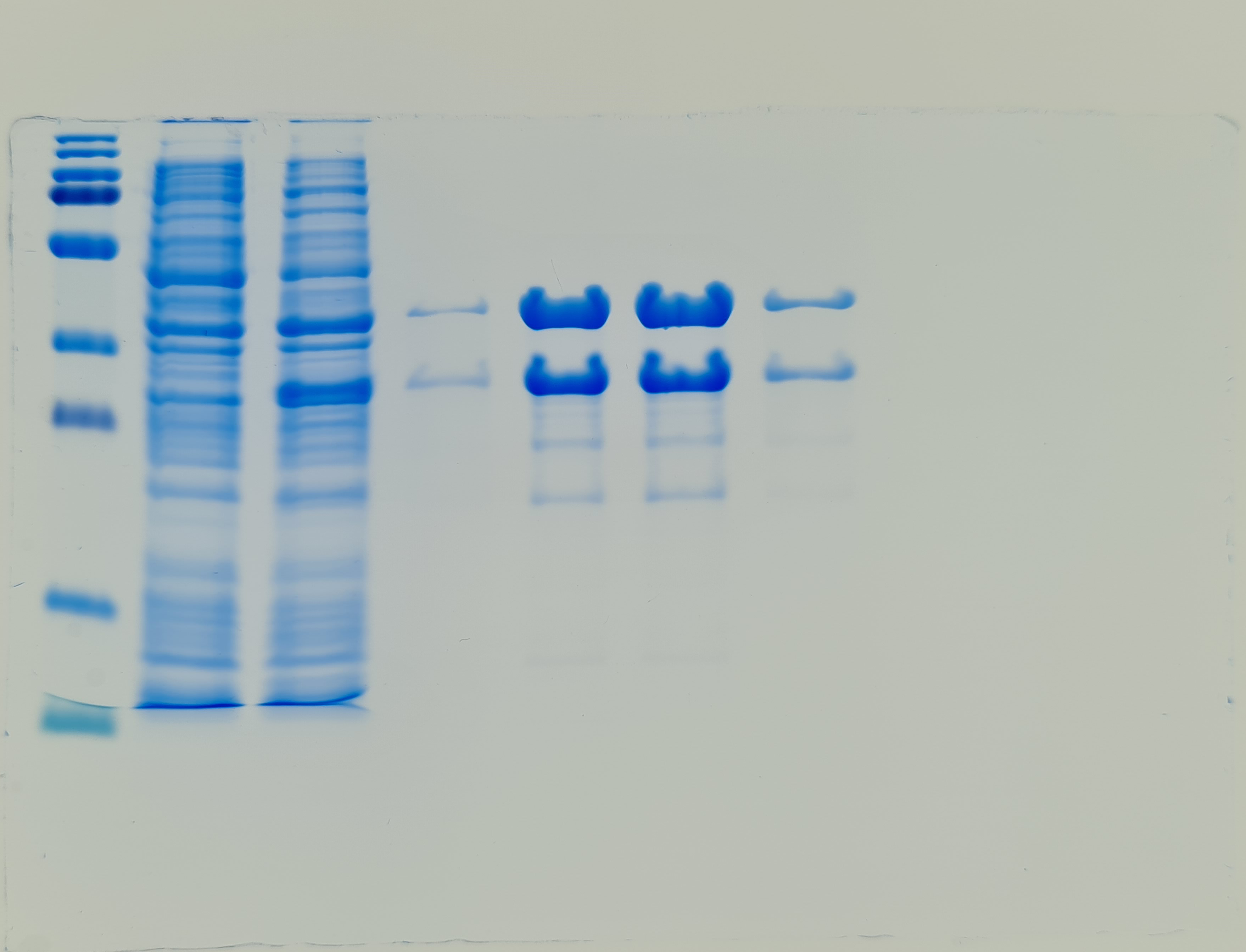

Supplement: Figure 5—figure supplement 1—source data 1. [file elife-82437-fig5-figsupp1-data1.zip › Figure 5- supplement 1 -source data 1/WT/TseV3-WT 1st affinity.tif]

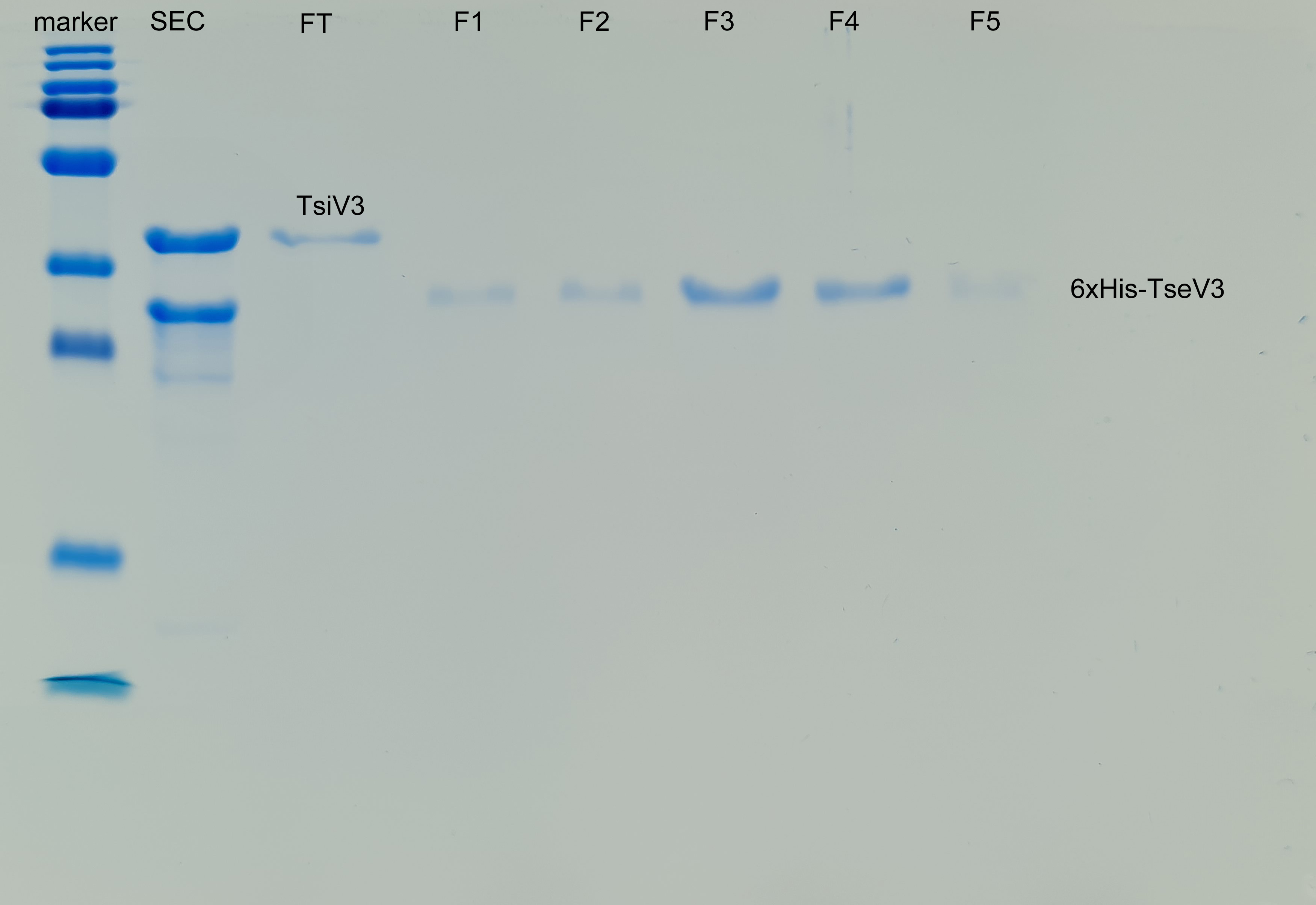

Supplement: Figure 5—figure supplement 1—source data 1. [file elife-82437-fig5-figsupp1-data1.zip › Figure 5- supplement 1 -source data 1/WT/TseV3-WT 2nd affinity pos urea denaturation labels.jpg]

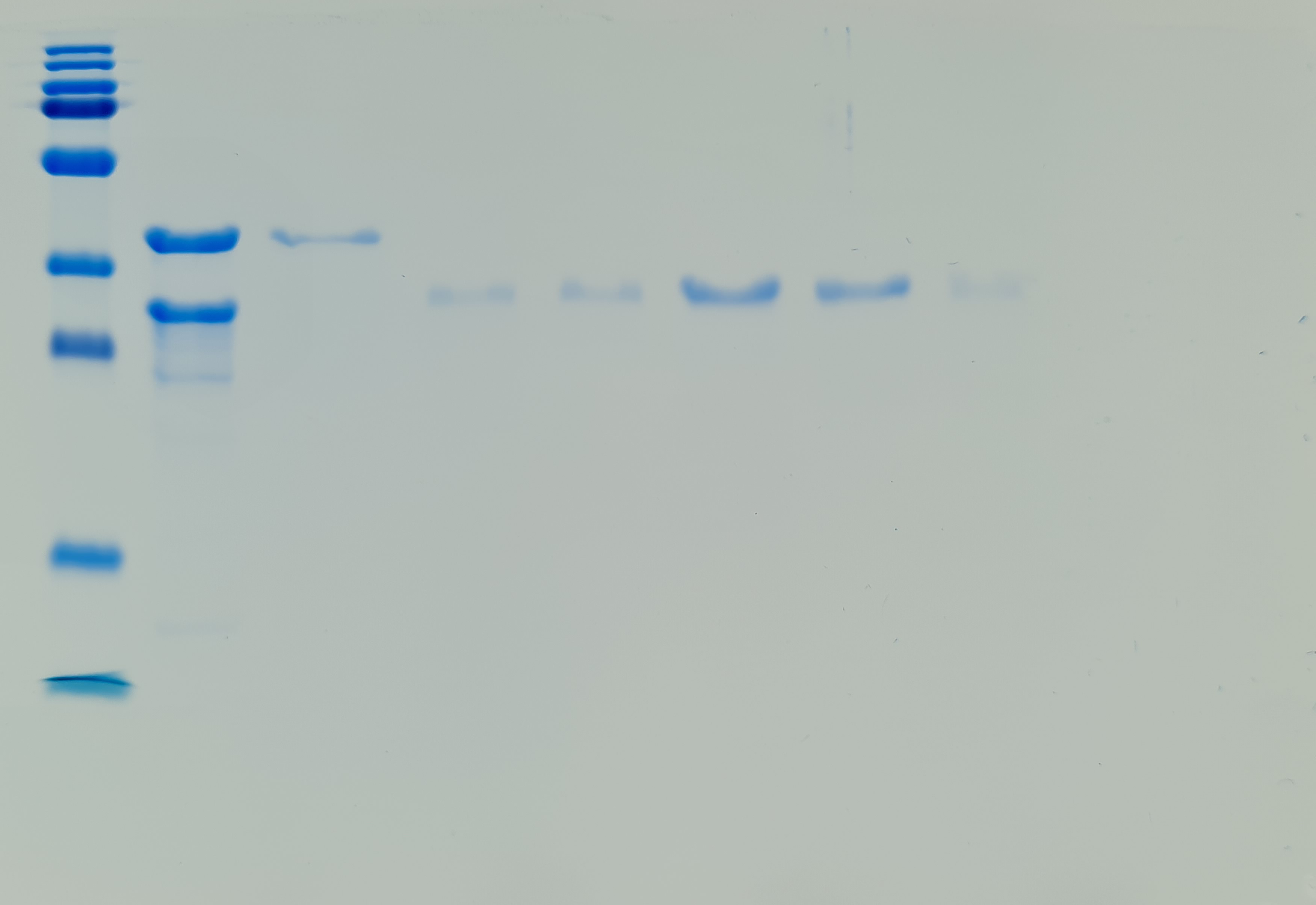

Supplement: Figure 5—figure supplement 1—source data 1. [file elife-82437-fig5-figsupp1-data1.zip › Figure 5- supplement 1 -source data 1/WT/TseV3-WT 2nd affinity pos urea denaturation.tif]

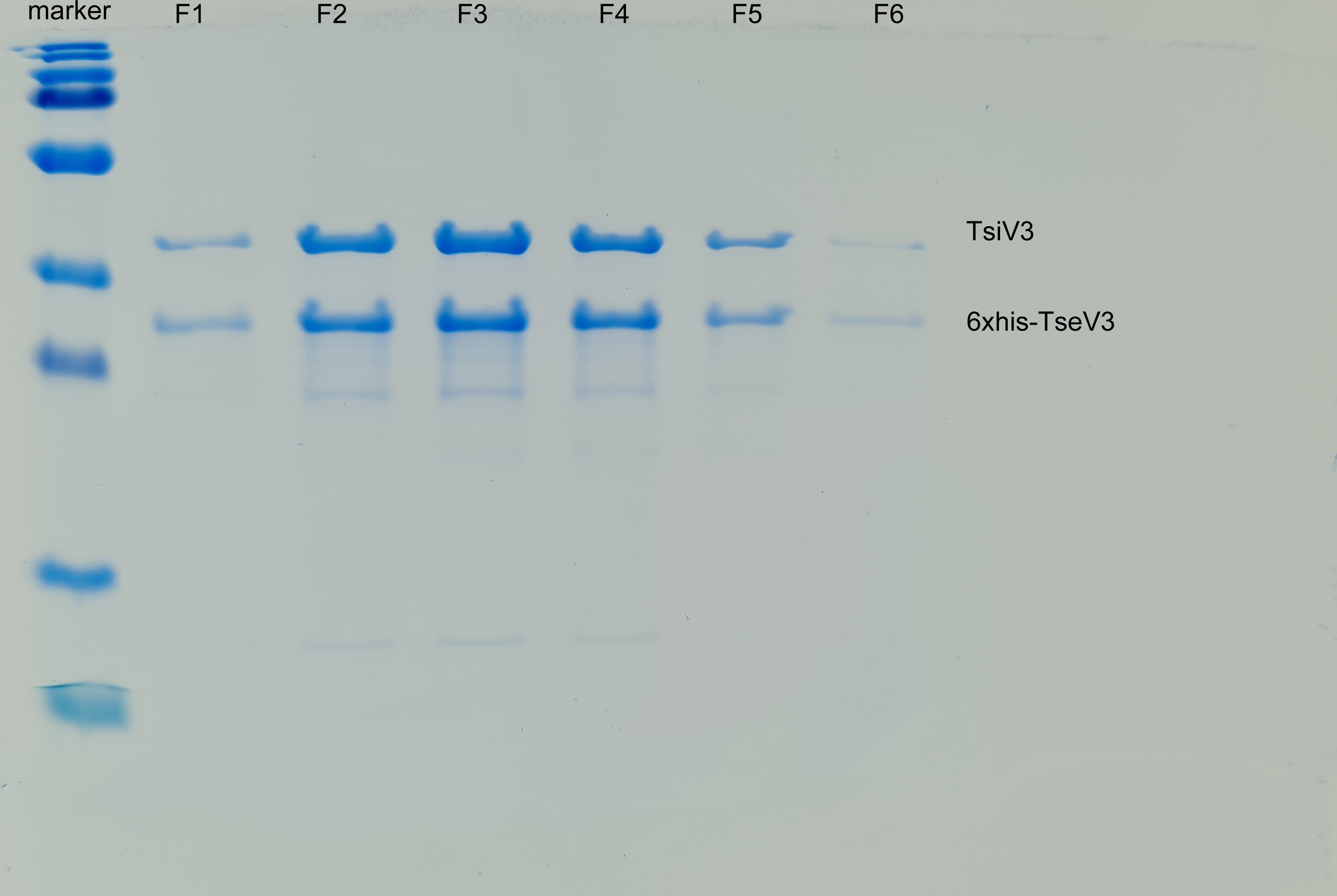

Supplement: Figure 5—figure supplement 1—source data 1. [file elife-82437-fig5-figsupp1-data1.zip › Figure 5- supplement 1 -source data 1/WT/TseV3-WT SEC labels.jpg]

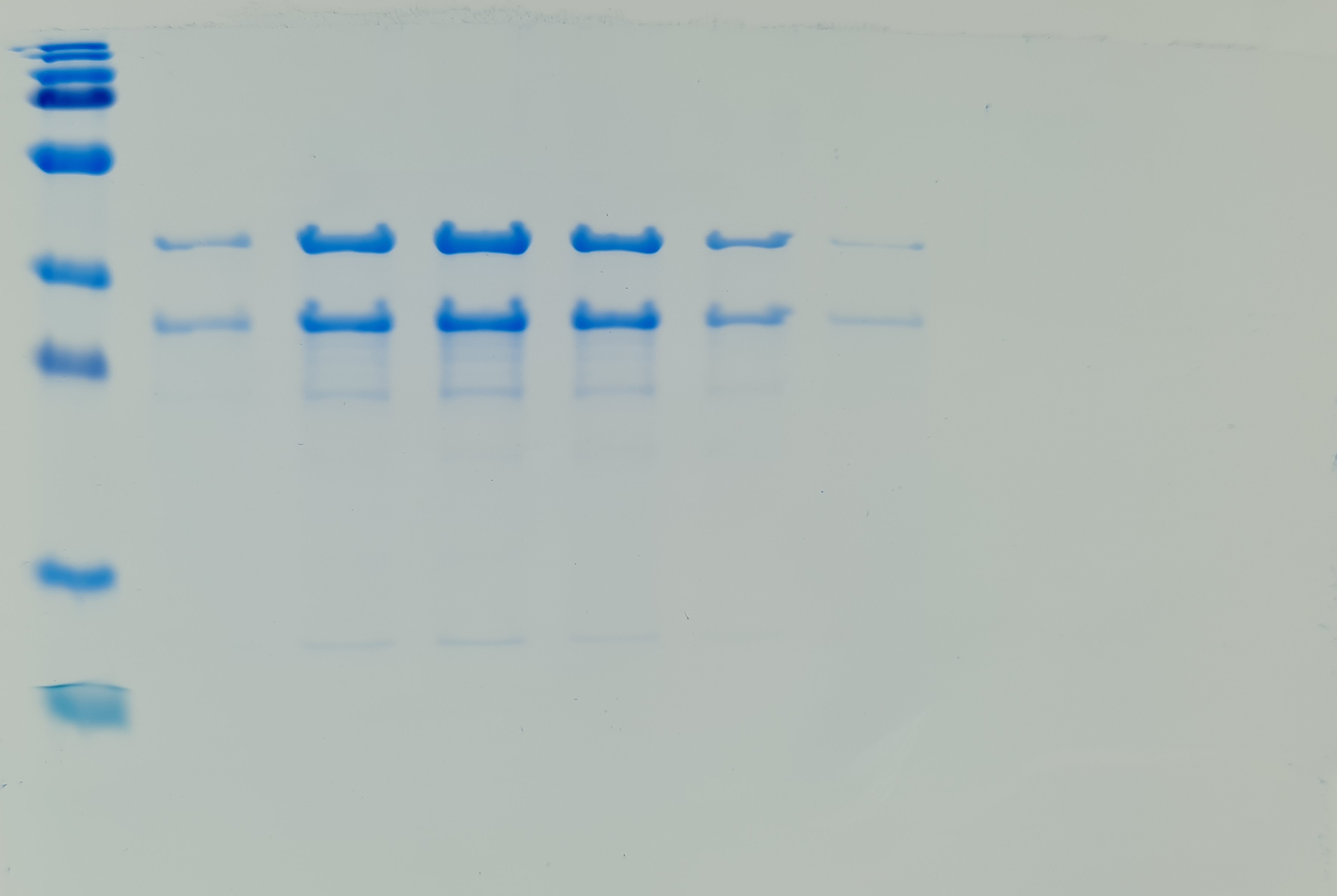

Supplement: Figure 5—figure supplement 1—source data 1. [file elife-82437-fig5-figsupp1-data1.zip › Figure 5- supplement 1 -source data 1/WT/TseV3-WT SEC.tif]

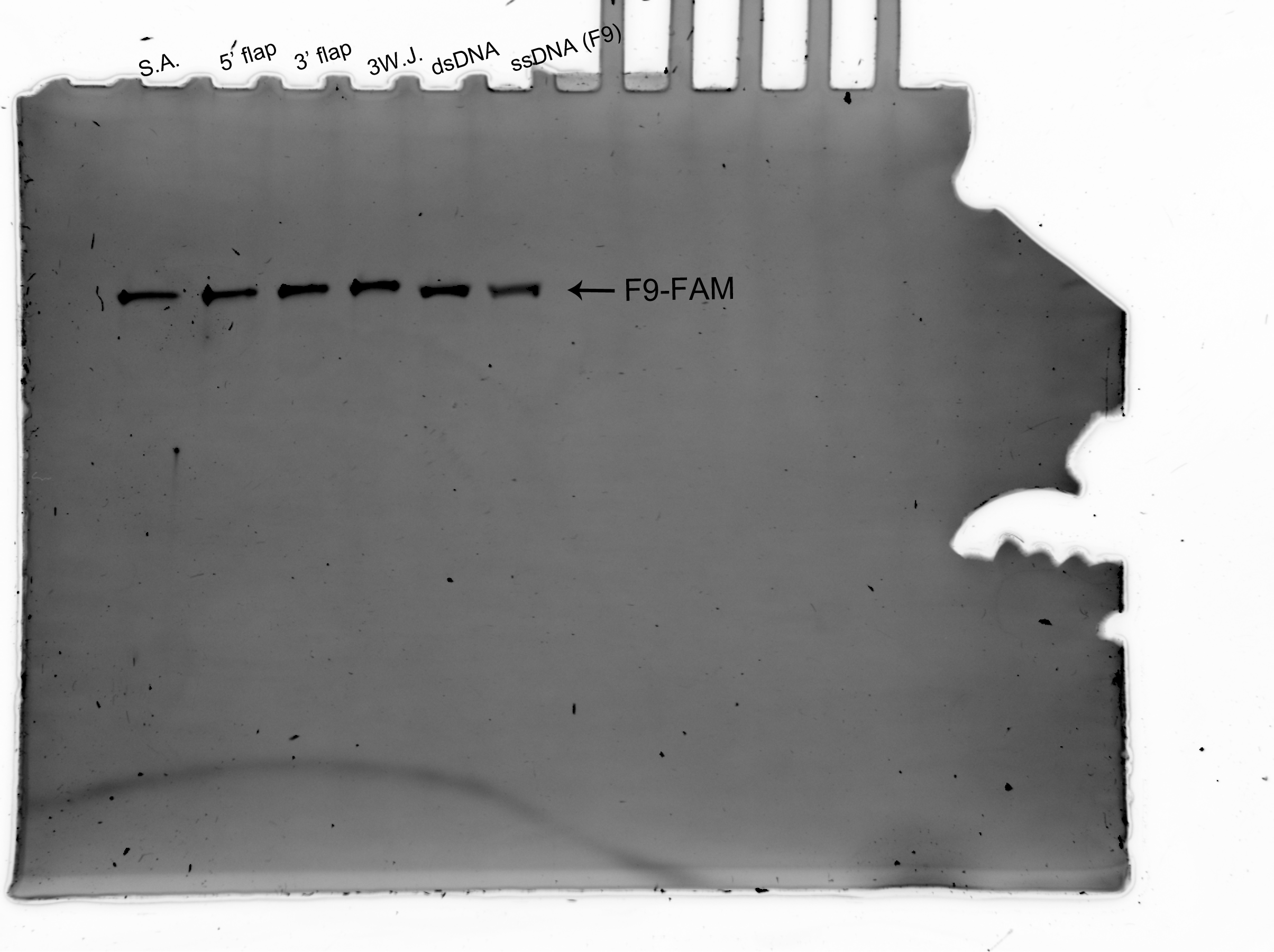

Supplement: Figure 5—figure supplement 1—source data 2. [file elife-82437-fig5-figsupp1-data2.zip › Figure 5- supplement 1- source data 2/Denaturing PAGE with substrates labels.tif]

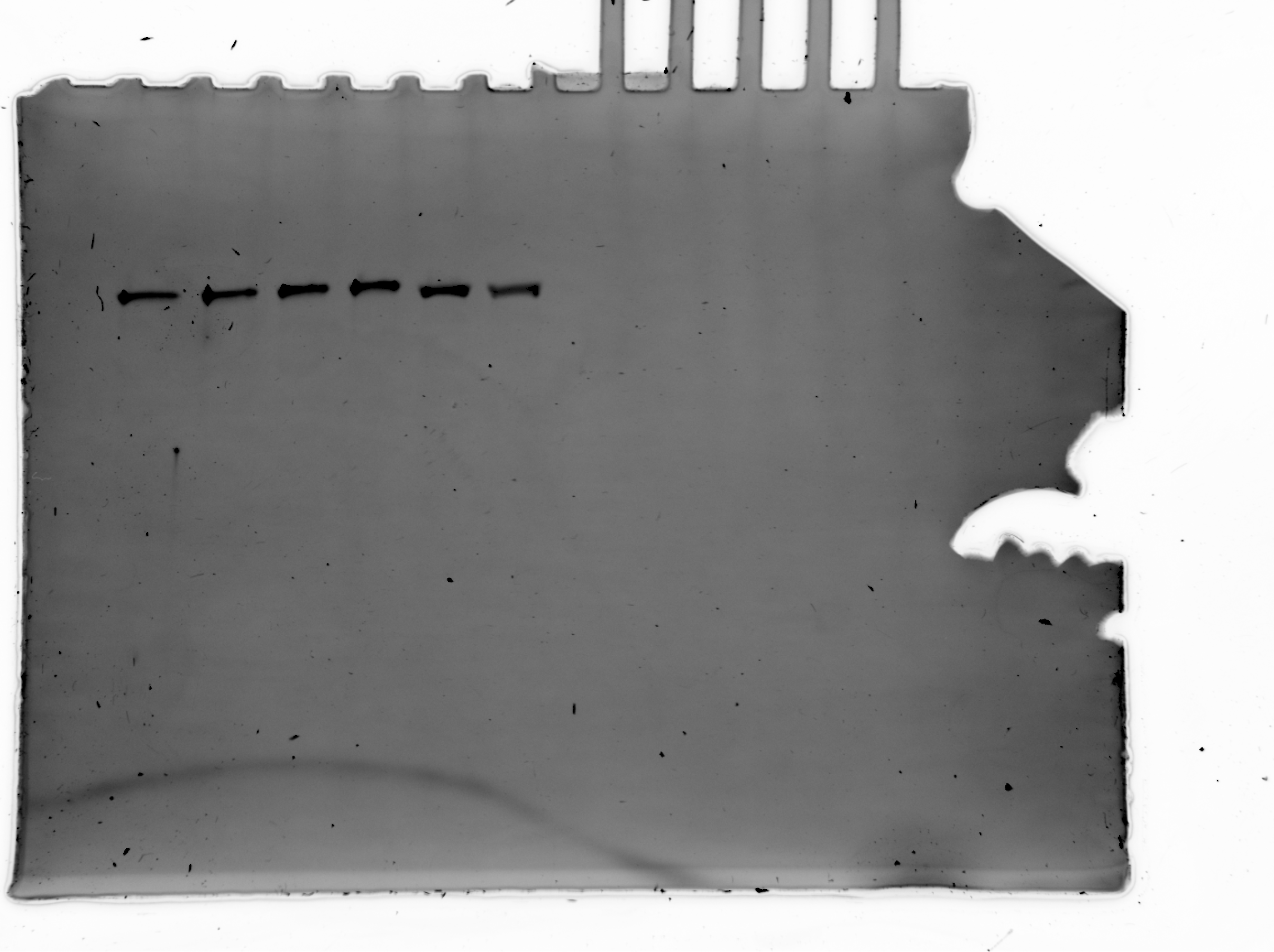

Supplement: Figure 5—figure supplement 1—source data 2. [file elife-82437-fig5-figsupp1-data2.zip › Figure 5- supplement 1- source data 2/Denaturing PAGE with substrates.tif]

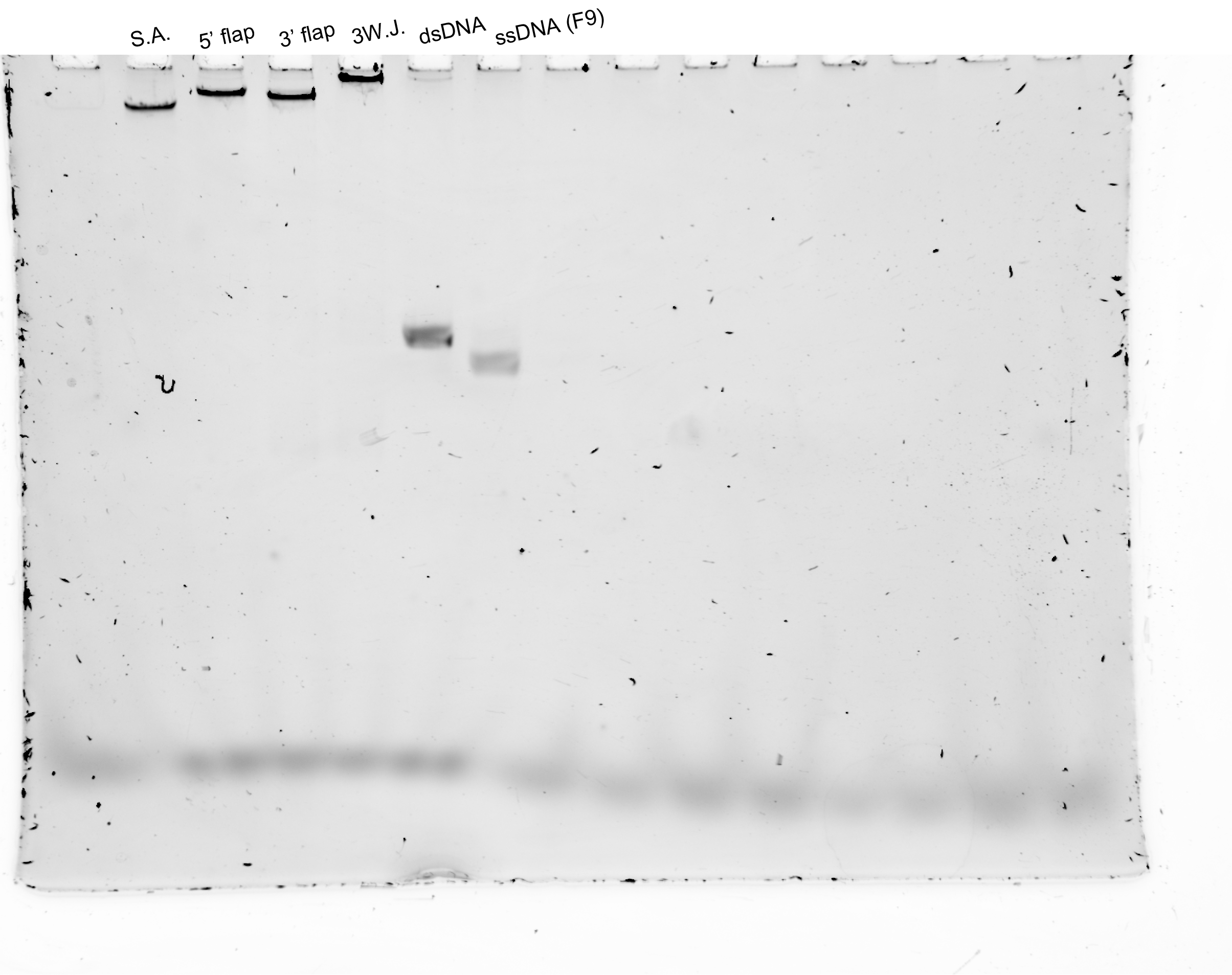

Supplement: Figure 5—figure supplement 1—source data 2. [file elife-82437-fig5-figsupp1-data2.zip › Figure 5- supplement 1- source data 2/Native PAGE with substrates labels.tif]

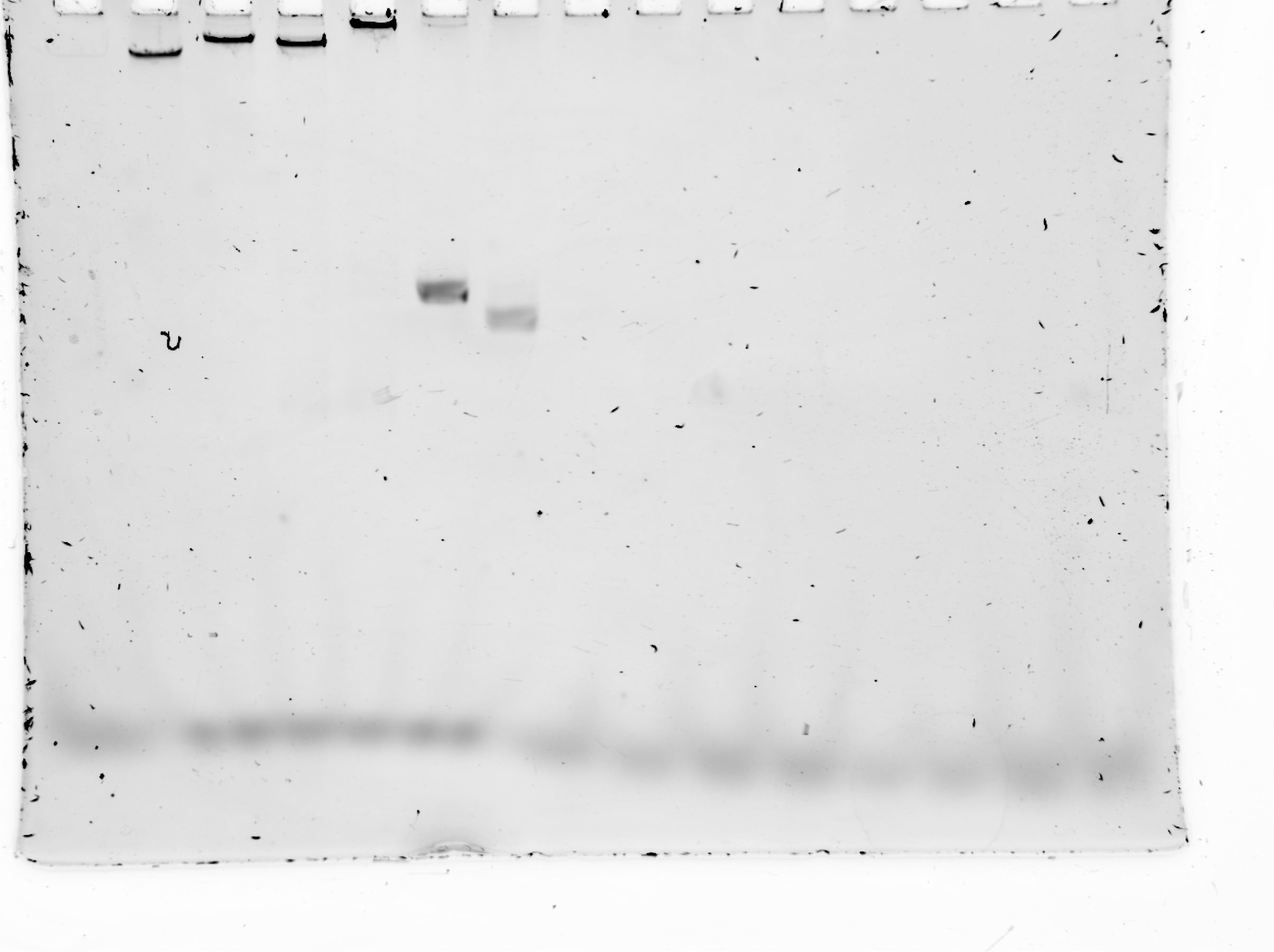

Supplement: Figure 5—figure supplement 1—source data 2. [file elife-82437-fig5-figsupp1-data2.zip › Figure 5- supplement 1- source data 2/Native PAGE with substrates.tif]

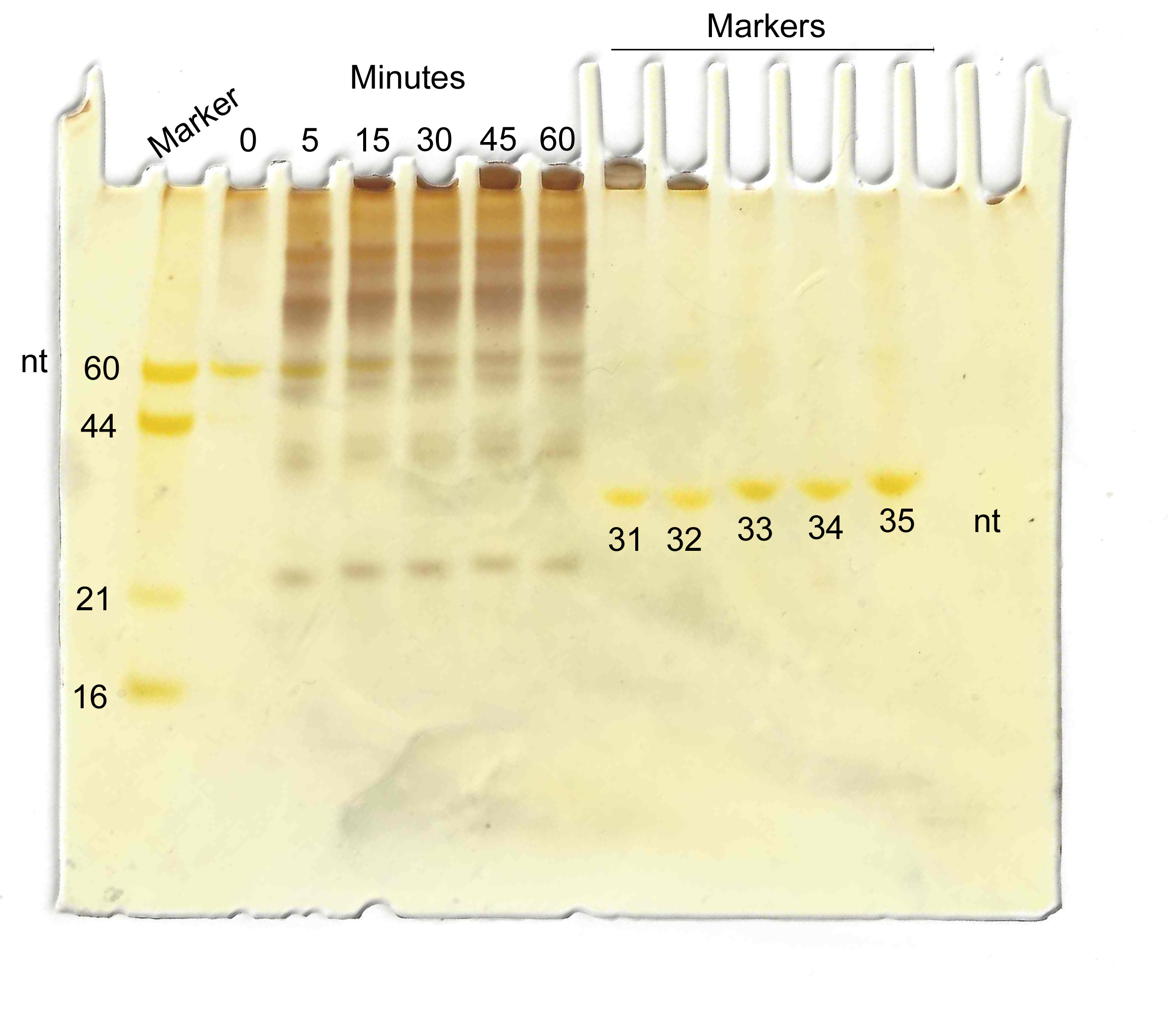

Supplement: Figure 5—figure supplement 1—source data 3. [file elife-82437-fig5-figsupp1-data3.zip › Figure 5- supplement 1- source data 3/Silver staining of time course degradation labels.tif]

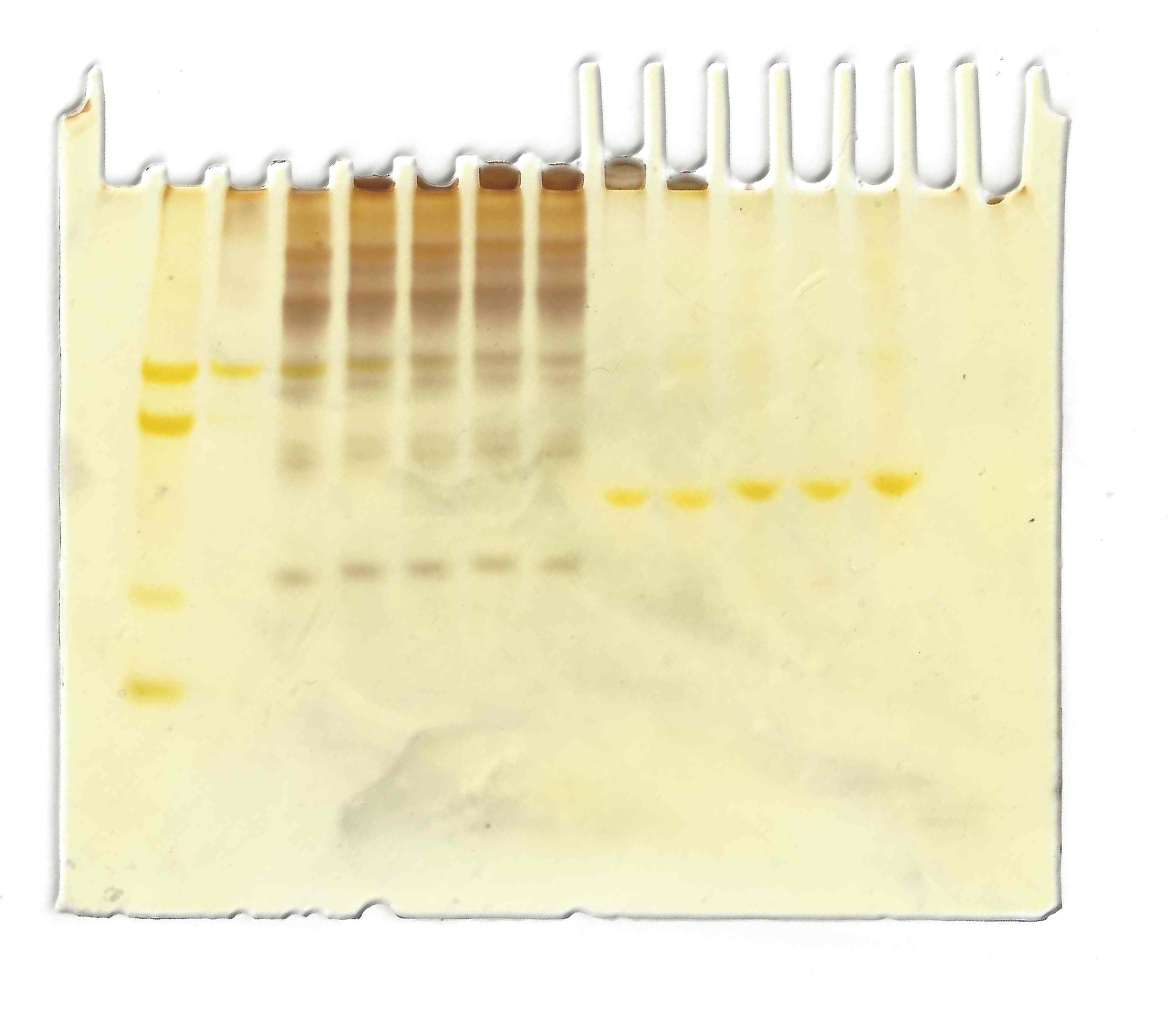

Supplement: Figure 5—figure supplement 1—source data 3. [file elife-82437-fig5-figsupp1-data3.zip › Figure 5- supplement 1- source data 3/Silver staining of time course degradation original.tif]

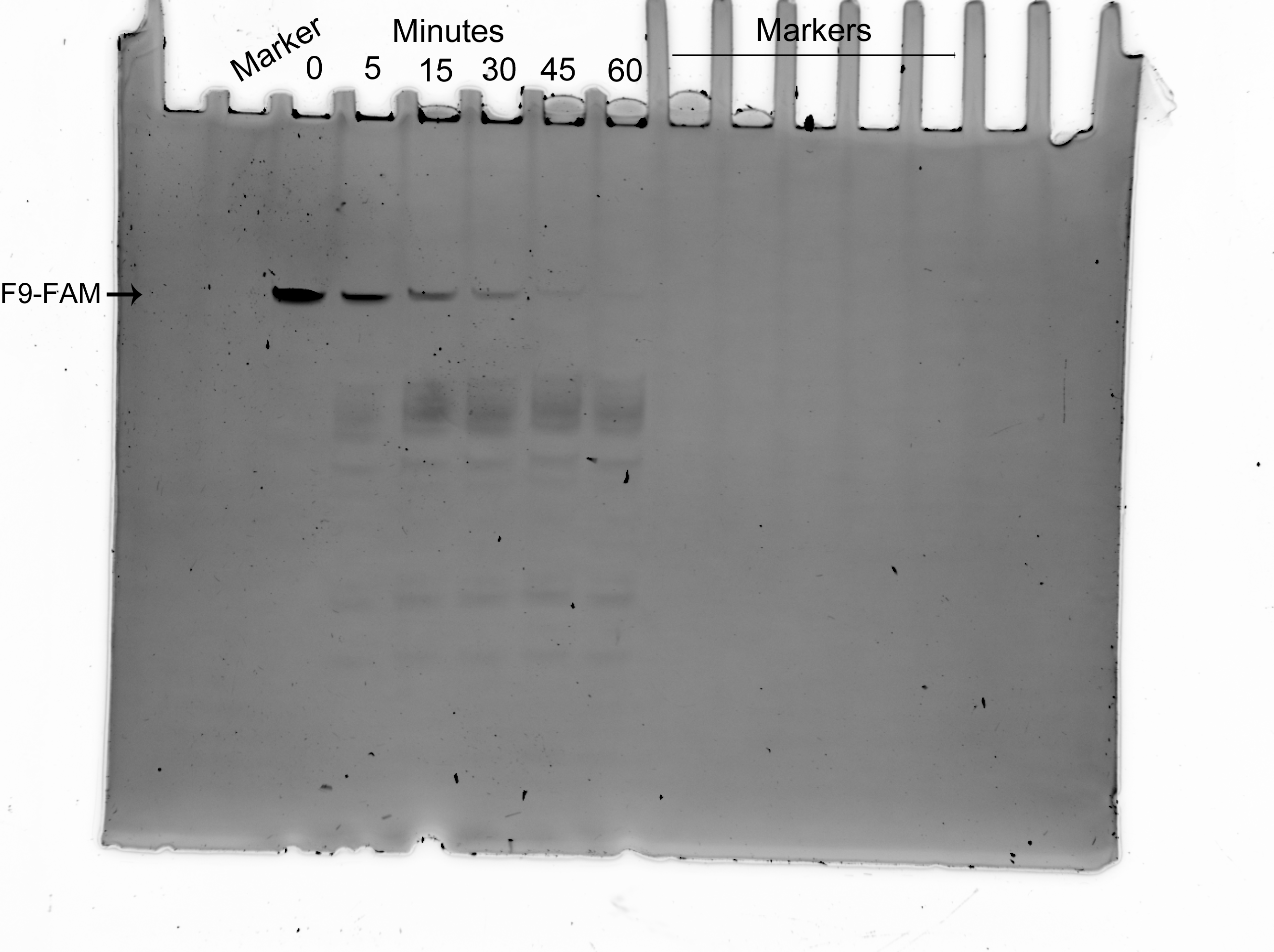

Supplement: Figure 5—figure supplement 1—source data 3. [file elife-82437-fig5-figsupp1-data3.zip › Figure 5- supplement 1- source data 3/Time course degradation with TseV3 labels.tif]

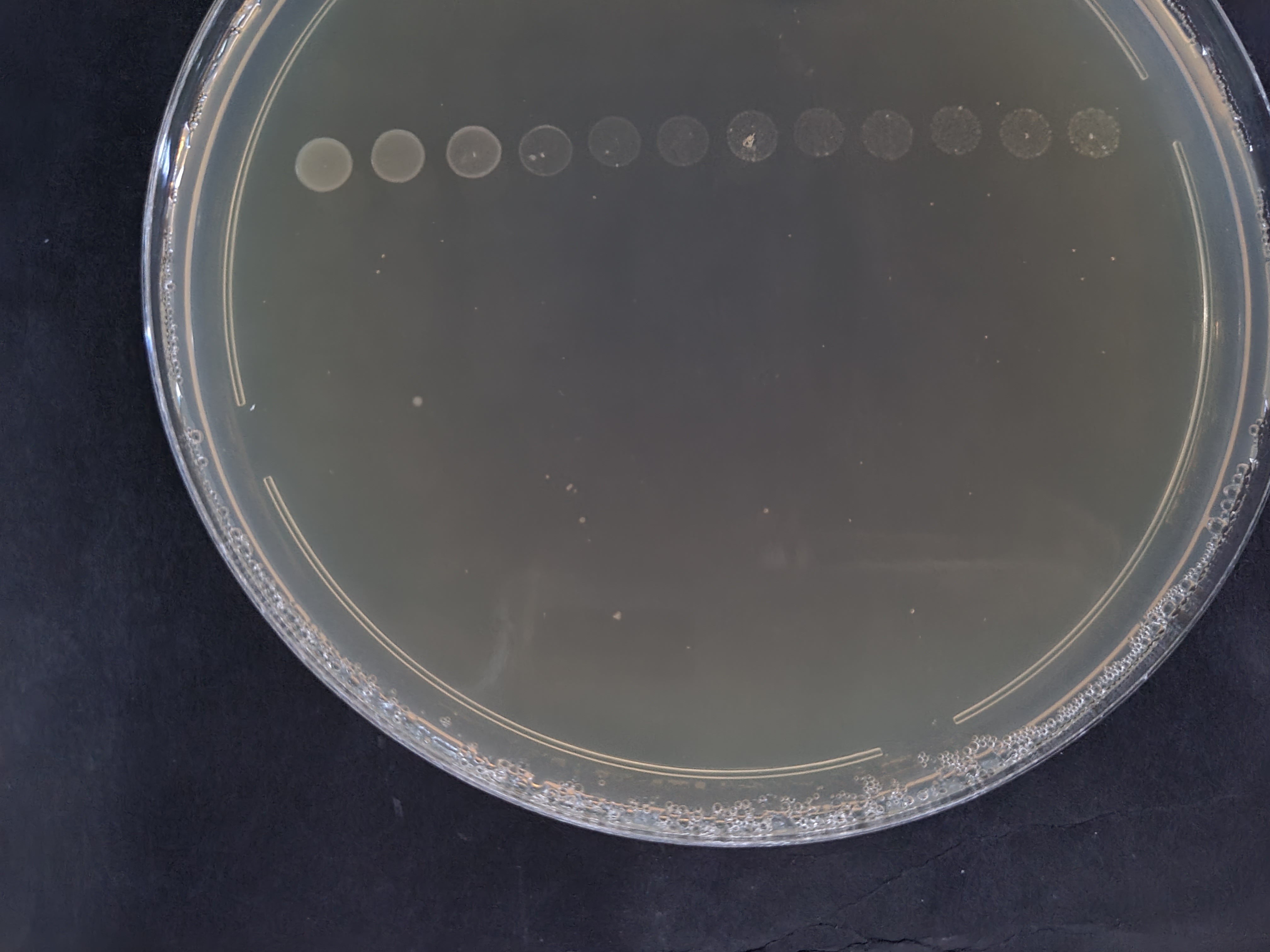

Supplement: Figure 6—source data 1. [file elife-82437-fig6-data1.zip › Figure 6- source data 1/Complex point mutation arab_iptg.jpg]

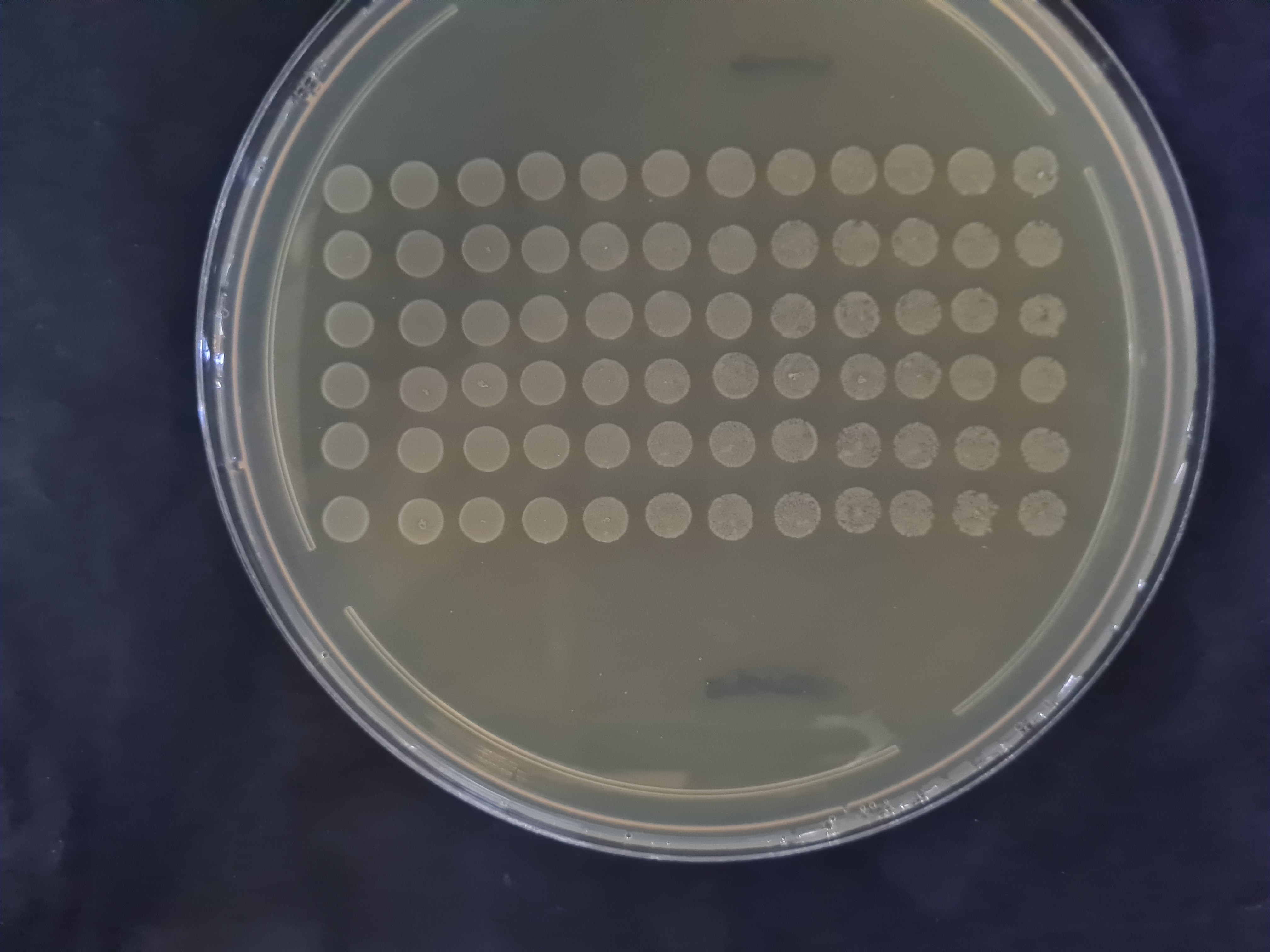

Supplement: Figure 6—source data 1. [file elife-82437-fig6-data1.zip › Figure 6- source data 1/Complex point mutation glucose.jpg]

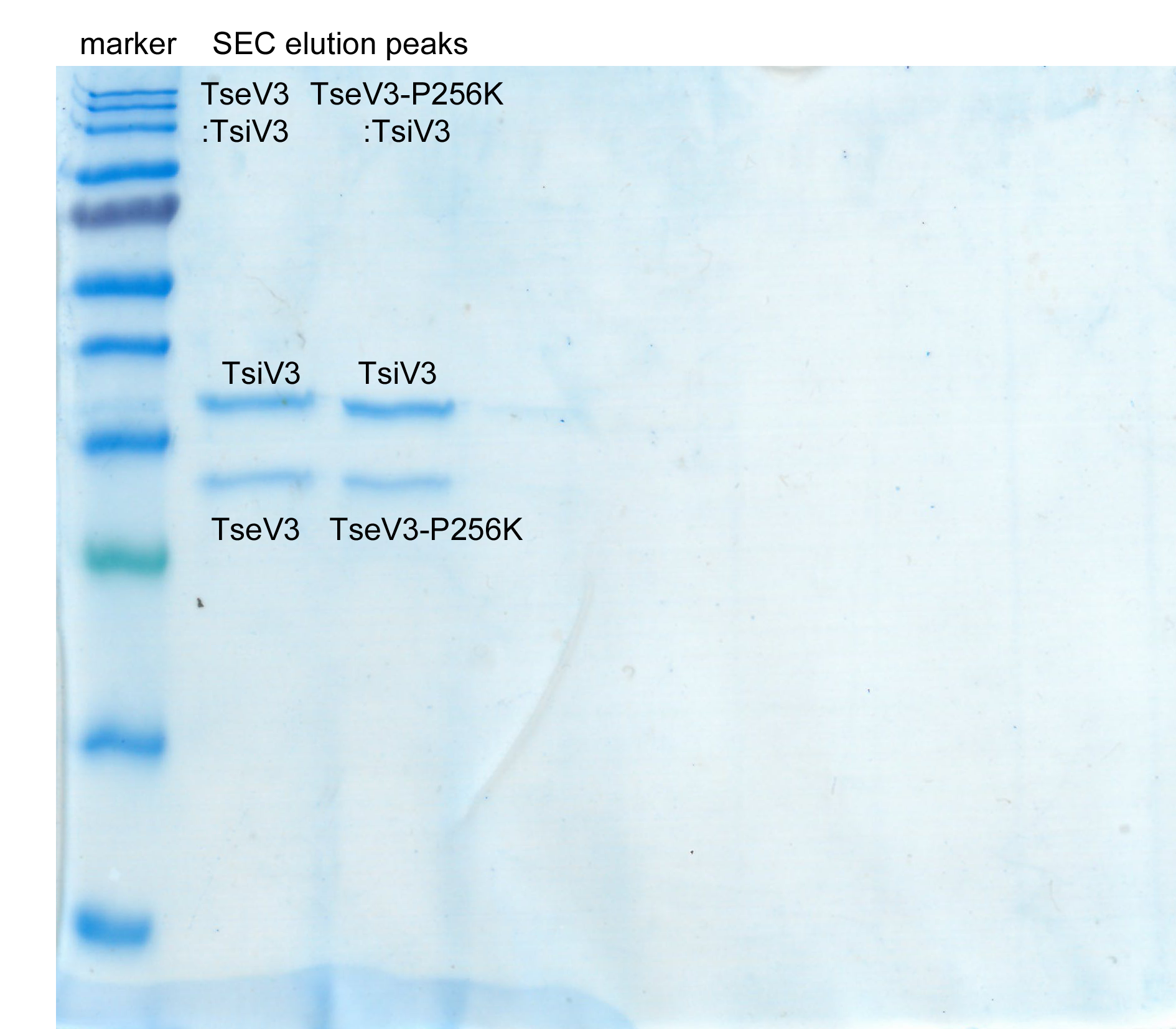

Supplement: Figure 6—figure supplement 1—source data 1. [file elife-82437-fig6-figsupp1-data1.zip › Figure 6- figure supplement 1- source data 1/SEC TseV3+TsiV3_TseV3-P256K+TsiV3 Gels labels.tif]

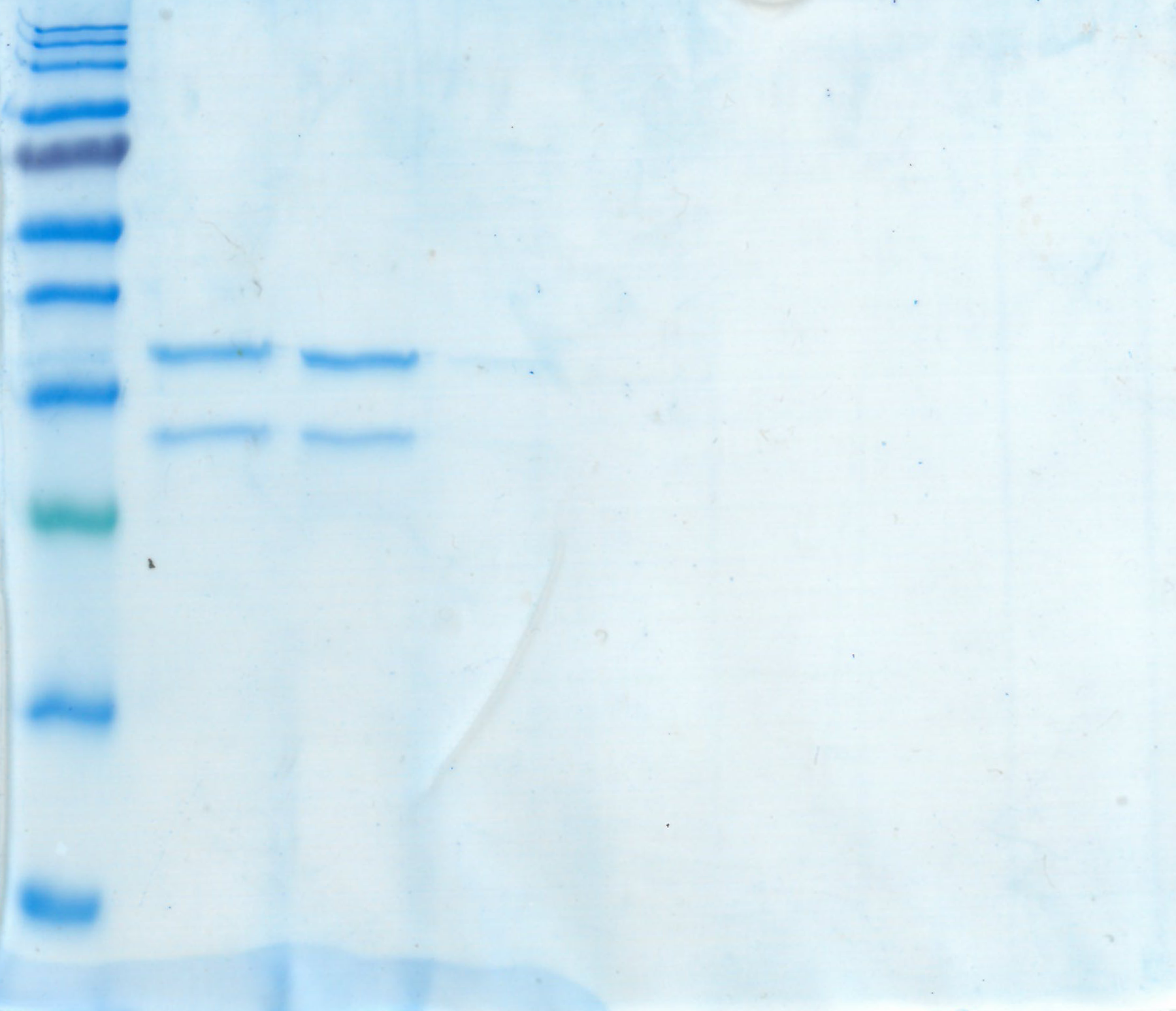

Supplement: Figure 6—figure supplement 1—source data 1. [file elife-82437-fig6-figsupp1-data1.zip › Figure 6- figure supplement 1- source data 1/SEC TseV3+TsiV3_TseV3-P256K+TsiV3 Gels.tif]

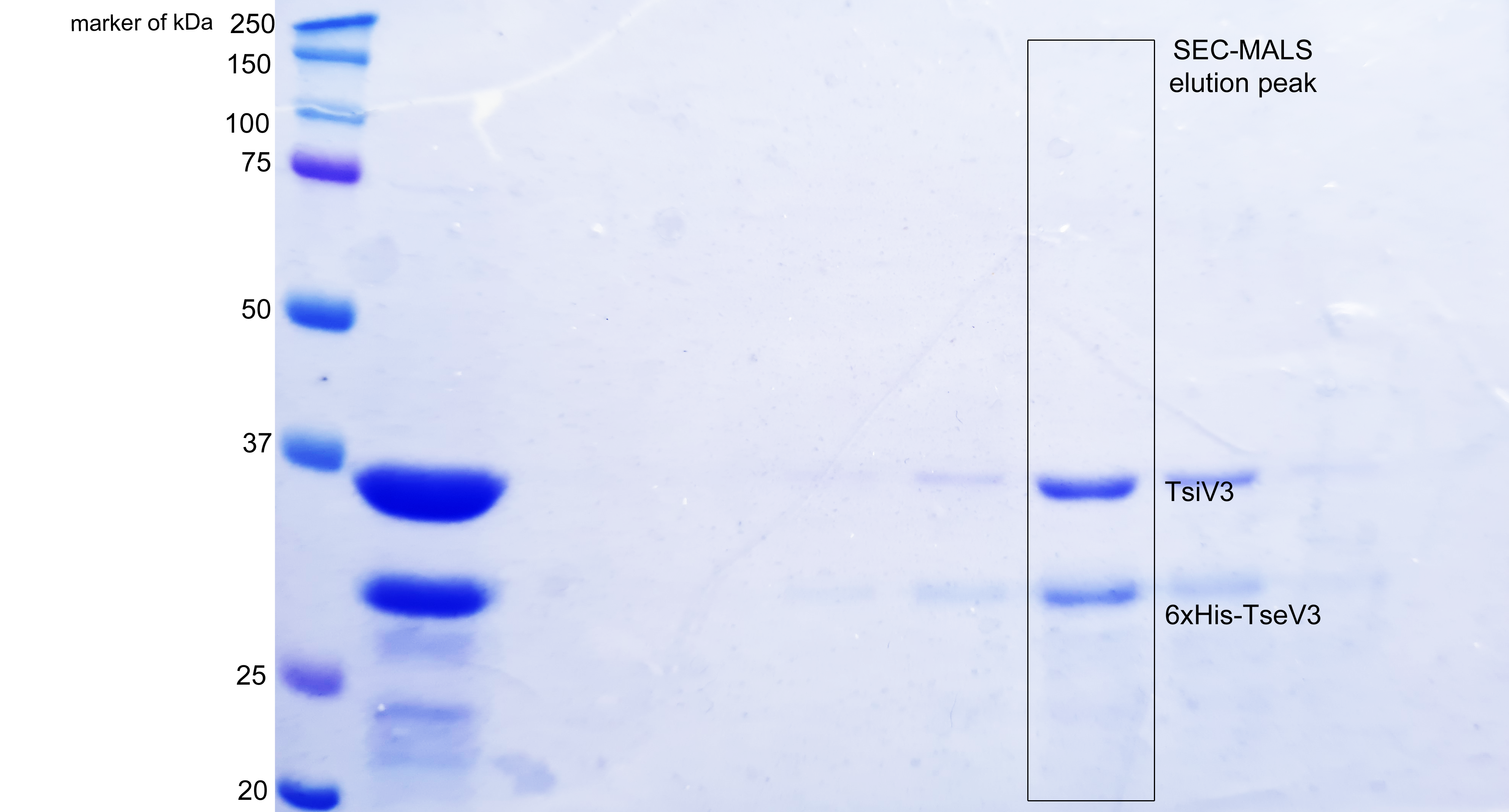

Supplement: Figure 6—figure supplement 1—source data 1. [file elife-82437-fig6-figsupp1-data1.zip › Figure 6- figure supplement 1- source data 1/SEC-Mals Gel labels.tif]

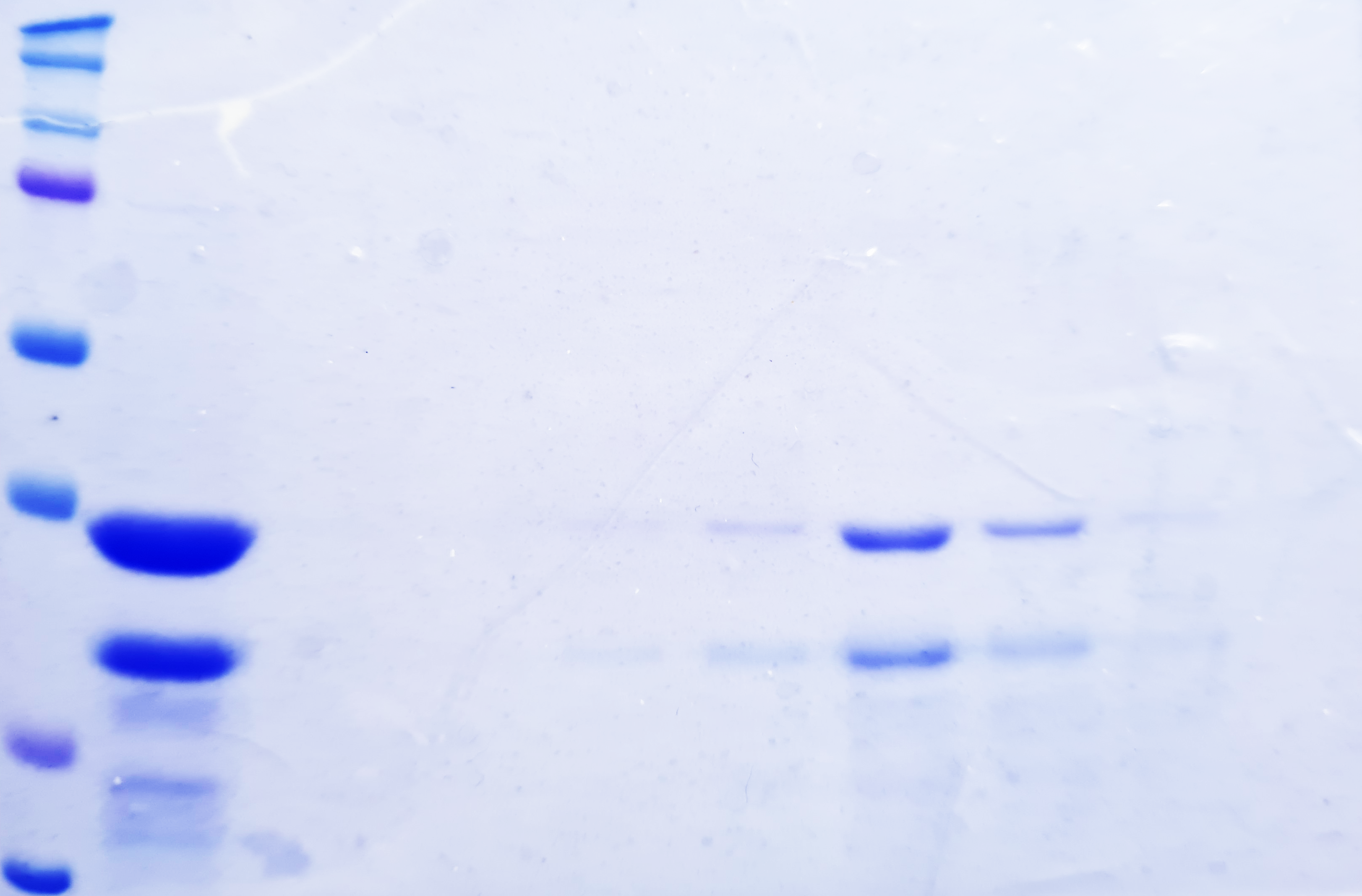

Supplement: Figure 6—figure supplement 1—source data 1. [file elife-82437-fig6-figsupp1-data1.zip › Figure 6- figure supplement 1- source data 1/SEC-Mals Gel original.tif]

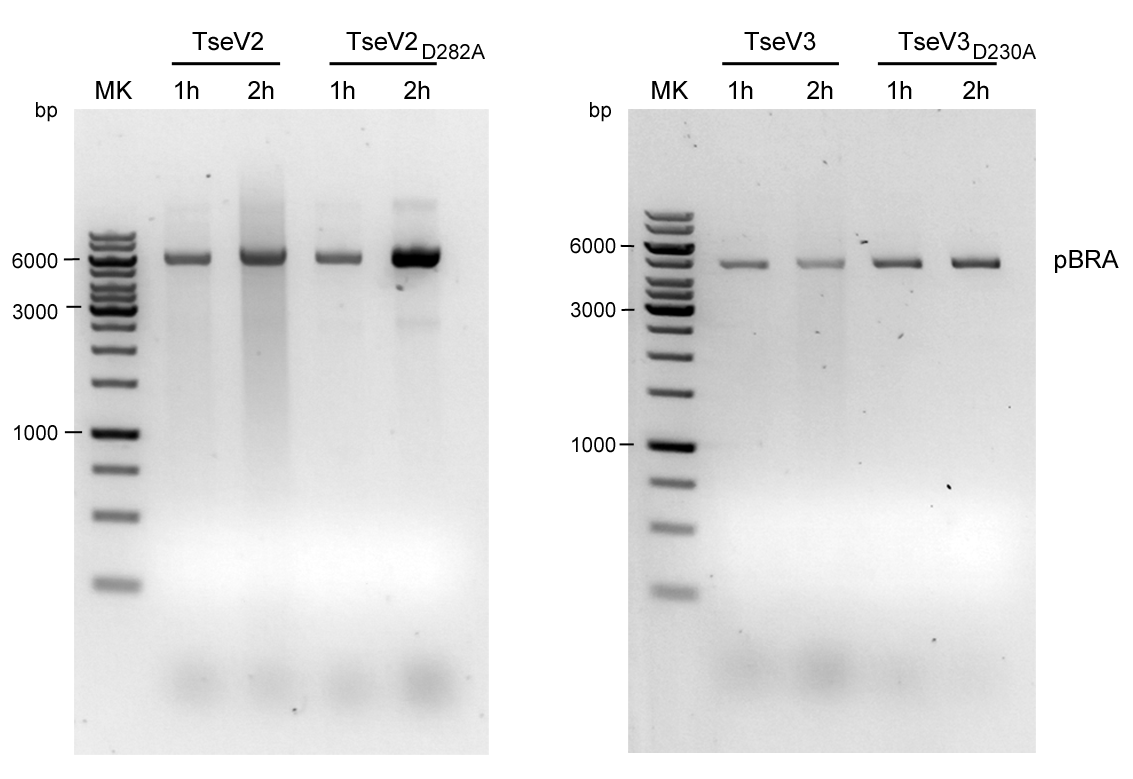

Supplement: Supplementary file 4. [file elife-82437-supp4.tif]

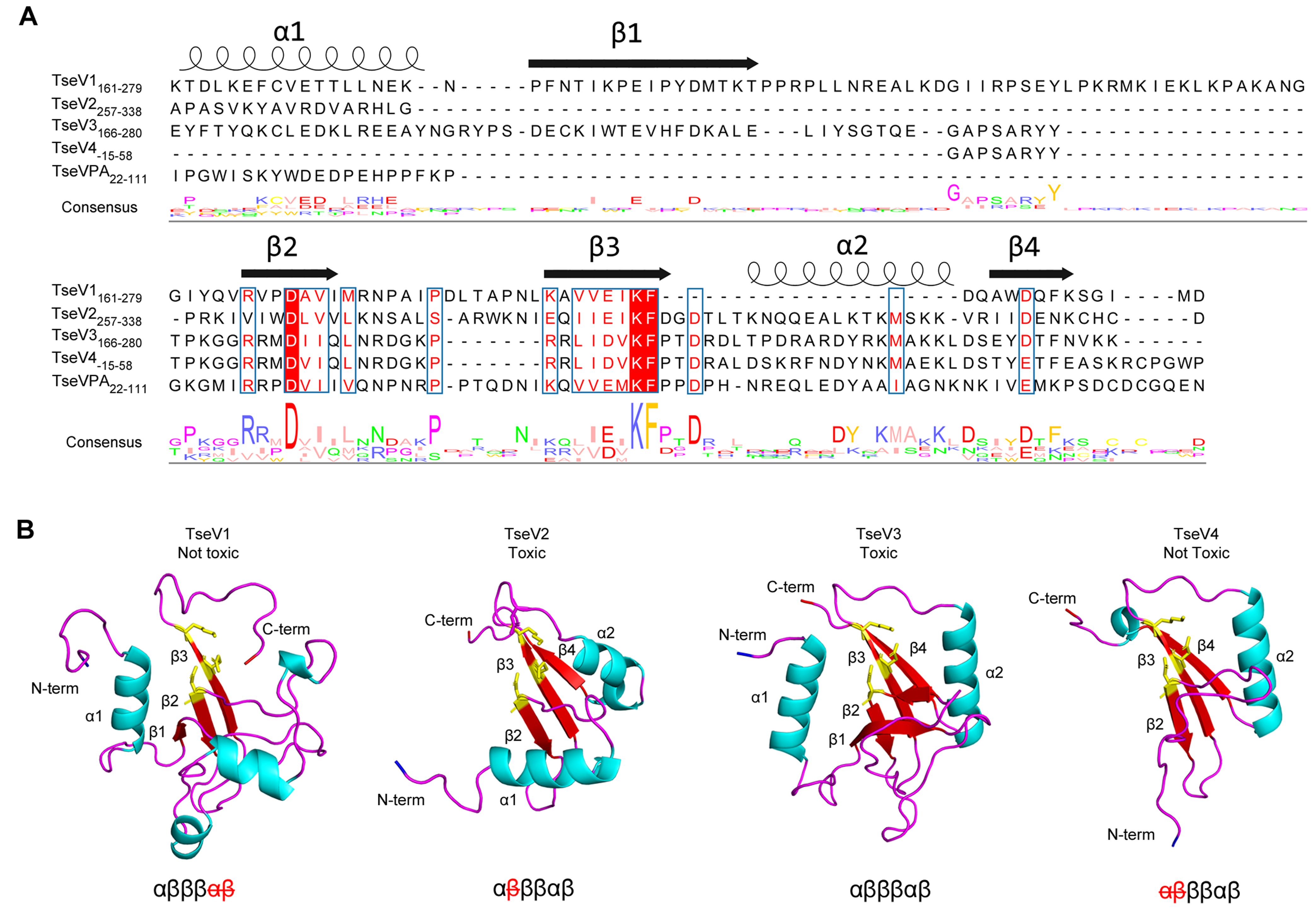

Supplement: Supplementary file 5. — (A) Manual amino acid sequence alignment of TseV14 and P. aeruginosa TseV (PA0822) based on secondary structures. The secondary structures are indicated above the alignments with α-helixes represented by spirals and β-sheets by arrows. The conserved catalytic residues are highlighted in red with the logo underneath the alignments. TseV4 contains another start codon located upstream of the annotated one. (B) TseV1–4 structures predicted by the AlphaFold (Jumper et al., 2021). Underneath is the conserved PD-(D/E)xK enzymatic core with the absent structures marked in dashed red. [file elife-82437-supp5.tif]
